# One-Pot Synthesis of Hydroxylated Alkaloids from Sugars via a Pictet–Spengler-Type Reaction

**DOI:** 10.3390/molecules29235709

**Published:** 2024-12-03

**Authors:** Likai Zhou, Na Ma, Jilai Wu, Weilin Yang, Lijing Feng, Song Xie, Lili Wang, Hua Chen

**Affiliations:** 1 Key Laboratory of Chemical Biology of Hebei Province, College of Chemistry and Material Science, Hebei University, Baoding 071002, China; zlk2wll@126.com (L.Z.);; 2 Functional Polymer Materials R&D and Engineering Application Technology Innovation Center of Hebei, College of Chemical Engineering and Biotechnology, Xingtai University, Xingtai 054001, China; 3 Asset Management Co., Ltd., Hebei University, Baoding 071002, China; 4 Comprehensive Experimental Center, Hebei University, Baoding 071002, China

**Keywords:** hydroxylated alkaloids, fused multicyclic iminosugars, Pictet–Spengler reaction, antitumor activity

## Abstract

An efficient and convenient strategy has been successfully developed for the preparation of novel hydroxylated alkaloid derivatives (also called fused multicyclic iminosugars) from *p*-toluenesulfonylated sugars through a Pictet–Spengler-type mechanism. This method is highly stereoselective, does not require metal catalysts, and capable of conducting gram level reactions (with a 53% yield). Some of such iminosugars had an intermediate antiproliferative effect on HCT116 tumor cells.

## 1. Introduction

**Figure 1:**
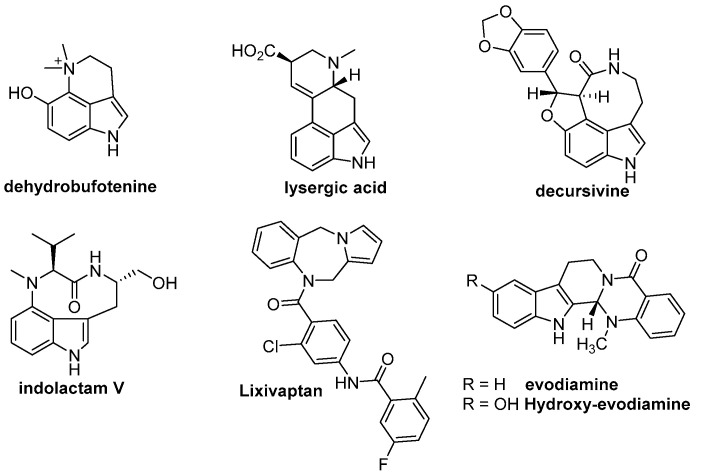
Alkaloids with good biological activities.

Indole or pyrrole, as the key structural skeleton, has been widely used in various therapeutic drugs, such as antitumor, antihypertensive, antiproliferative, antiviral, analgesic, anti-inflammatory, and antibacterial drugs, due to its alkaloid character. Currently, there are over a hundred indole or pyrrole drugs on the market [1,2]; the preparation [3,4] and modification of these [5,6,7,8] have been hot research topics in the field of organic synthesis. Fusing an additional ring at the 3, 4-positions of indole is a common strategy in the structural modification of indole, and well-known products of this process include dehydrobufotenine [9,10], lysergic acid [11,12], decursivine [13,14,15], and indolactam V [16,17,18] (Figure 1). Indolactam V not only can selectively activate protein kinase C (PKC) but can also induce the differentiation of human embryonic stem cells (ESCs) into pancreatic cells. Lixivaptan is a typical pyrrole alkaloid in the development of innovative non-peptide oxytocin and vasopressin small-molecule agonists and antagonists [19] (Figure 1). Additionally, fused indole alkaloids, such as evodiamine, have excellent antiproliferative activities in tumor cells, while the hydroxylated type, 10-hydroxyevodiamine [20], as a clinical chemotherapy drug, shows better antitumor activity than that of evodiamine, which suggests that the introduction of hydroxy groups into fused alkaloids can improve their biological activities, with the exception of their bioavailability.

**Scheme 1:**
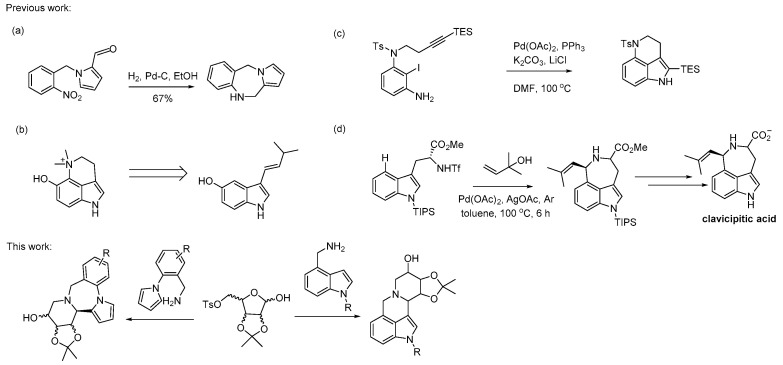
Different strategies for the synthesis of alkaloids. (**a**) O’Brien’ work [26]; (**b**) Wang’s work [27]. (**c**) Jia’s work [28]; (**d**) References [29,30].

Due to the excellent biological activities of indole and pyrrole derivatives, many research groups have developed different synthetic methods for such alkaloids [21,22,23,24,25]. O’Brien’s research group successfully constructed a pyrrole-like alkaloid by utilizing a tandem hydrogenation–condensation–hydrogenation sequence [26] (Scheme 1a). Wang’s research group achieved efficient synthesis of toad alkaloid dehydrobufotenine in eight steps with an overall yield of 8% from 5-methoxyindole [27] (Scheme 1b). Jia’s research group conducted a lot of outstanding work in the synthesis of alkaloids, including the synthesis of 3,4-fused tricyclic indoles using intra-molecular Larock indole synthesis [28] (Scheme 1c). In addition, through C-H activation, Pd-catalyzed direct alkylation of the tryptophan derivatives at the C-4 position was undertaken to prepare 4-substituted tryptophan, which could be used for the synthesis of various sesquiterpenoid indole alkaloids [29,30] (Scheme 1d).

However, the fusion of carbohydrates into indole or pyrrole alkaloids to construct new bioactive small molecules has rarely been reported. Considering the potent activity of hydroxylated alkaloids, herein, we would like to report the synthesis of novel indole- or pyrrole-type derivatives containing an iminosugar under metal-free catalytic conditions through a Pictet–Spengler-type reaction [31,32].

## 2. Results and Discussion

### 2.1. Optimization of the Reaction Conditions

Recently, our group has been dedicated to the study of the synthesis of fused multicyclic iminosugars [33,34]. An effective approach has been developed for synthesizing hydroxylated alkaloid **2a** (also called a fused multicyclic iminosugar) using 4-(aminomethyl)indole **1a** and *p*-toluenesulfonylated sugars as the raw materials. This reaction can successfully synthesize various five membered, six membered, or seven membered iminosugar indole alkaloids by heating in an oil bath at 120 ° C in an air atmosphere without a metal catalyst.

d-lyxose tosylate (**1aa**) and 1*H*-indol-4-ylmethanamine (**1a**) were selected as the model substrates to optimize the conditions (Table 1). Firstly, compound **1a** (1.5 equiv) was reacted with d-lyxose tosylate (**1aa**) in the presence of the metal catalysts Yb(OTf)_3_, Sc(OTf)_3_, and AlCl_3_ for 5 h, respectively, all of which were able to generate target product **2a**, albeit at yields of 30–38% (entries 1–3). Subsequent attempts to conduct the reaction under metal-free reaction conditions, by employing TFA or 1N HCl as the acid catalysts, did not yield improvements (entries 4–5).

Without any catalyst, the expected product **2a** could also be generated smoothly, and the yield slightly improved (entry 6). Varying the amount of **1a** under 80 °C reaction conditions, it was found that **2a** was achieved at a moderate yield of 58% when 1.2 equiv of **1a** was used (entries 7–8). When the reaction was performed at increasing reaction temperatures, the yield gradually increased and reached the highest amount of 66% at 120 °C (entries 9–10). No more attempts were made above 120 °C. Finally, when toluene, DMSO, and THF were employed as different reaction solvents, the reactions produced target compound **2a** at low yields (entries 11–13). In addition, the reaction could also be successfully carried out under the optimal conditions in a nitrogen atmosphere (entry 14).

### 2.2. Synthesis of the Iminosugar Alkaloids

**Figure 2:**
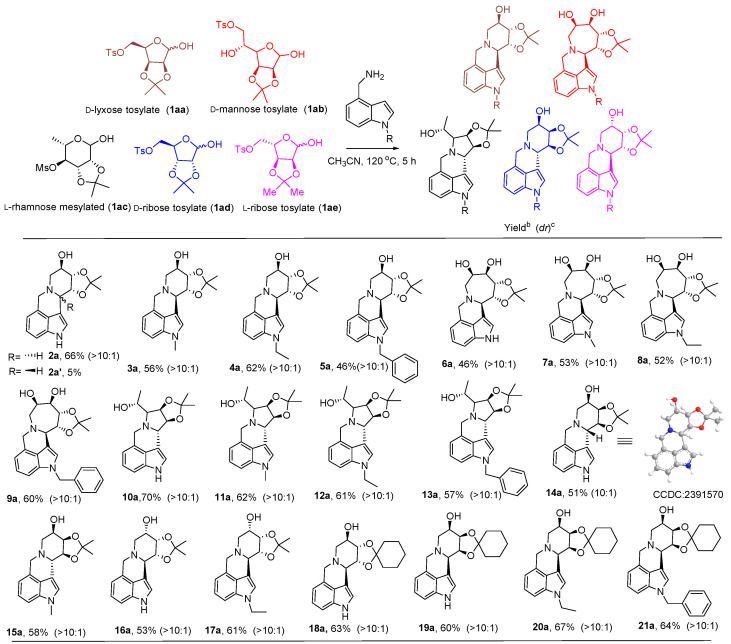
Synthesis of the iminosugar indole alkaloids. Reaction conditions: d/l-lyxose/ribose tosylate, d-mannose tosylate, or l-rhamnose sulfonate (0.2 mmol, 1.0 equiv), aminomethyl-indole (1.2 equiv), CH_3_CN (2.0 mL), air, 120 °C, 5 h, oil bath heating; ^b^ isolated yields; ^c^ determined using ^1^H NMR; (*dr*): the *dr* was determined by ^1^H NMR of the mixture.

Under the optimized reaction conditions, the substrate tolerance was studied, and a series of hydroxylated 3,4-position-fused indole alkaloids were prepared (Figure 2). By using different *N*-substituents, such as methyl, ethyl, benzyl, and different Ts-glycosides, specific products can be obtained. By reacting d-lyxose tosylate (**1aa**) or D-ribose tosylate (**1ad**) or L-ribose tosylate (**1ae**) with 3-methylaminoindole, products **2a**–**5a** and **14a**–**17a** were obtained at a moderate yield of 46–66%, respectively. The reaction of D-mannose tosylate (**1ab**) and indoleamine generated seven-membered-iminosugar-fused indole derivatives **6a**–**9a**. When L-rhamnose mesylate (**1ac**) was used as the substrate, the reactions resulted in the formation of five-membered-iminosugar-fused indole alkaloids **10a**–**13a**. Finally, the reaction was carried out replacing the isopropyl group with the cyclohexyl group on the sugar, which yielded products **18a**–**21a**. NMR spectrum of **2a**–**21a** is shown in the Figures S1–S42 in the Supplementary Materials. To sum up, a series of five-, six-, and seven-membered-iminosugar-fused indole alkaloids with high diastereomeric ratios (above 10:1) was successfully one-pot-synthesized. The single-crystal data for compound **14a** (CCDC: 2391570, The single-crystal data Figure S90 in the Supplementary Materials) confirmed the structure.

**Figure 3:**
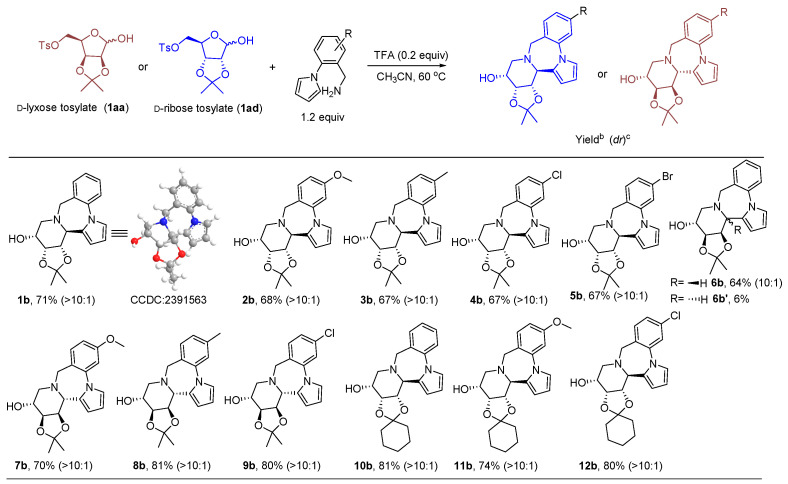
Synthesis of the iminosugar pyrrole benzodiazepine alkaloids. Reaction conditions: D-lyxose/ribose tosylate (0.2 mmol, 1.0 equiv), 2-(1-pyrrolyl)benzylamine (1.2 equiv), TFA (0.2 equiv), CH_3_CN (2.0 mL), air, 60 °C, 4 h, oil bath heating; ^b^ isolated yields. ^c^ determined using ^1^H NMR; (*dr*): the *dr* was determined by ^1^H NMR of the mixture.

After optimization of the reaction conditions, it was found that under the catalysis of trifluoroacetic acid, reactions of D-ribose tosylate or d-lyxose tosylate and 2-(1-pyrrolyl)benzylamine at 60 °C generated six-membered iminosugar benzopiperazine pyrrole alkaloids **1b**–**9b**. The single-crystal data for compound **1b** confirmed the structure (CCDC: 2391563, The single-crystal data Figure S91 in the Supplementary Materials). The expected products were also successfully obtained when the sugar was protected with a cyclohexyl group, **10b**–**12b** (Figure 3). NMR spectrum of **1b**–**12b** is shown in the Figures S43–S68 in the Supplementary Materials.

**Scheme 2:**
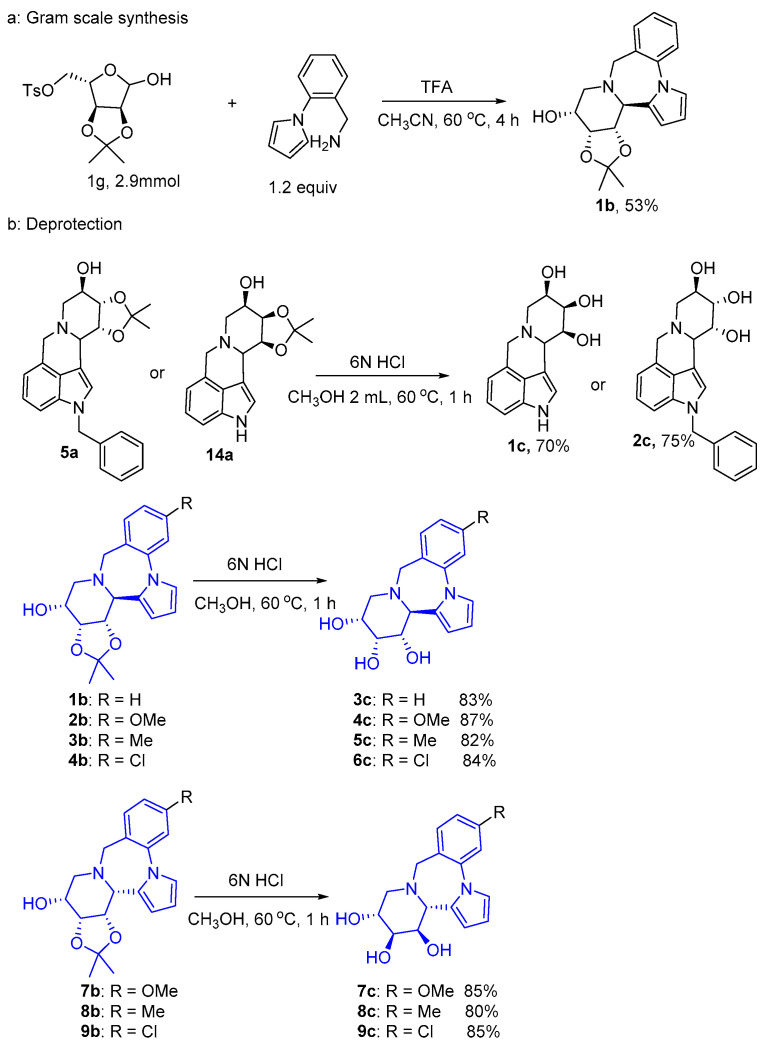
Further transformations of the derivatives.

When the reaction was scaled up to the gram level, product **1b** could be obtained at a 53% yield (Scheme 2a). Additionally, removing 2,3-*O*-isopropylidene in the presence of hydrochloric acid to generate corresponding products **1c**–**9c**), respectively (Scheme 2b). NMR spectrum of **1c-9c** is shown in the Figures S69–S86 in the Supplementary Materials.

**Scheme 3:**
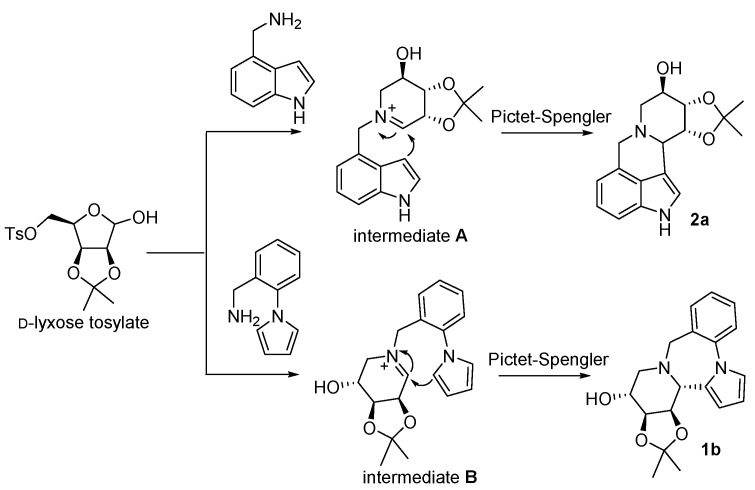
A possible mechanism for the synthesis of the alkaloids.

According to the above experimental results, a reasonable reaction mechanism is described (Scheme 3). Firstly, the reaction of d-lyxose tosylate and an amine yields a key intermediate iminium ion **A** or **B** [33,34]. Then, an intramolecular Pictet–Spengler-type [35] reaction will occur to form the final compound **2a** or **2b**, respectively, due to the high nucleophilic reactivities of indole or pyrrole.

Iminosugars **9a** and **6c** were preliminarily evaluated for their antitumor activities. The cytotoxicity of these compounds against HCT116 (human colon cancer cells) was examined using Cell Counting Kit-8 (CCK-8) assays after 48 h of drug treatment. The results showed that both compounds could inhibit the growth of the cells, and the inhibitory rates were above 45% at a concentration of 100 μM, which suggested such iminosugars had intermediate antiproliferative activities against HCT116 tumor cells.

## 3. Materials and Methods

### 3.1. General Information

The solvents were all analytical-grade, and the other reagents were purchased from Energy Chemical (Shanghai, China) and Bide Pharmatech Ltd (Shanghai, China). The ^1^H NMR spectra were measured on 600 MHz and 400 MHz Bruker AVANCE spectrometers (Fällanden, Switzerland). The ^13^C NMR spectra were recorded on Bruker 100 MHz spectrometers (Fällanden, Switzerland) with complete proton decoupling. Melting points were measured on glass slides on an SGW X-4 melting point apparatus (Shanghai Yidian Physical & Optical Instrument Co., Shanghai, China). Optical rotations were determined on an SGW-1 automatic polarimeter (Shanghai Yidian Physical & Optical Instrument Co., Shanghai, China). High-resolution mass spectrometry (HRMS) were conducted on an FTICR-MS (Ionspec 7.0T) mass spectrometer with electric spray ionization (ESI) (Bruker Daltonics, Billerica, MA, USA), manufactured by Ionspec Company in the United States. Thin-layer chromatography (TLC) was performed on pre-coated plates (GF254) with detection using UV light, and silica gel (200–300-mesh) was used for column chromatography (Qingdao Puke Spectrum Separation Material Co., Qingdao, China). X-ray diffraction data were gathered using a Bruker D8 VENTURE (Bruker, Bremen, Germany).

### 3.2. Extraction and Isolation

General experimental procedure: Ts-d-lyxose (69 mg, 0.2 mmol) and aminomethyl-indole (1.2 equiv) were added into a 20 mL flask, with 2.0 mL of CH_3_CN used as the solvent. Then, the solution was stirred at a temperature of 120 °C under an air atmosphere for 5 h. Upon completion, the mixture was cooled to room temperature, and the solvent was evaporated in vacuo. The crude product was purified using column chromatography (dichloromethane:methanol *v/v* = 30:1) to give **2a**–**5a** as a pale white solid. Under similar conditions, different *N*-substituents 3-aminoindoles, Ts-d-mannose, Ms-l-rhamnose, Ts-d-ribose, and Ts-l-ribose, were used as the raw materials for the reaction, and the corresponding products were obtained, respectively (**6a**–**17a**).

Ts-d-ribose (69 mg, 0.2 mmol), 2-(1-pyrrolyl)benzylamine (1.2 equiv), and trifluoroacetic acid (0.2 equiv) were added into a 20 mL flask, with 2.0 mL of CH_3_CN used as the solvent. Then, the solution was stirred at a temperature of 60 °C under an air atmosphere for 4 h. Upon completion, the mixture was cooled to room temperature, and the solvent was evaporated in vacuo. The crude product was purified using column chromatography (petroleum ether/ethyl acetate *v/v* = 1:1) to give **1b**–**10b** as a pale white solid.

Product **5a** (30 mg, 0.07 mmol) or **14a** and 6N HCl (5.0 equiv) were added into a 20 mL flask, with 2.0 mL of methanol used as the solvent. Then, the solution was stirred at a temperature of 60 °C under an air atmosphere for 2 h. Upon completion, the mixture was cooled to room temperature, and the solvent was evaporated in vacuo. The crude product was purified using silica gel column chromatography (dichloromethane:methanol *v/v* = 15:1) to give **1c** or **2c** as a pale white solid. Under similar conditions, compounds **1b**–**4b**, **7b**–**9b** were reacted with 1N HCl (5.0 equiv) to obtain compounds **3c**–**9c**.

Ts-d-ribose (1 g, 2.9 mmol), 2-(1-pyrrylyl)benzylamine (1.2 equiv), and TFA (0.2 equiv) were added into a 50 mL flask, using 5.0 mL of CH_3_CN as the solvent. Then, the solution was stirred at a temperature of 60 °C under an air atmosphere for 4 h. Upon completion, the mixture was cooled to room temperature, and the solvent was evaporated in vacuo. The crude product was purified using silica gel column chromatography (petroleum ether/ethyl acetate *v/v* = 1:1) to obtain a 501 mg light yellow solid 1b with a yield of 53%.

### 3.3. Antitumor Activity

Cell Counting Kit-8 (CCK-8, APExBlO Technology LLC, Houston, TX, USA) assays were used to measure the cell viability. Cells were seeded into a 96-well plate at a density of 3000–5000 cells/well with 100 μL of complete culture medium. The tested compounds were added to the wells at different concentrations, and the plates were incubated at 37 °C for 48 h. Then, 10 μL of CCK-8 reagent was added to each well of the plate, which was kept in the dark for 2 h. The absorbance value at 450 nm was measured using a microplate reader to evaluate the cell viability. Calculate the survival rate according to the public notice (1) provided in the instruction manual

survival rate = (ODtreated − ODblank)/(ODcontrol − ODblank) × 100%(1)

## 4. Conclusions

In summary, we have developed a novel method for the one-pot synthesis of iminosugar piperidone indole alkaloids and benzopiperazine pyrrole alkaloids, featuring simple operation and a stable structure which offers an efficient pathway for the construction of valuable alkaloid derivatives. Meanwhile, the absolute structure of the required product was confirmed using single-crystal X-ray diffraction.

## Figures and Tables

**Table 1:** Optimization of reaction conditions for compound **2a**.

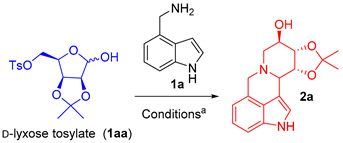					
Entry	1a (equiv)	Catalyst (0.2 equiv)	Solvent (2.0 mL)	Temp (°C)	Yield ^b^ (%)
1	1.5	Yb(OTf)_3_	CH_3_CN	80	30
2	1.5	Sc(OTf)_3_	CH_3_CN	80	35
3	1.5	AlCl_3_	CH_3_CN	80	38
4	1.5	CF_3_COOH	CH_3_CN	80	37
5	1.5	1N HCl	CH_3_CN	80	24
6	1.5	-	CH_3_CN	80	42
7	2.0	-	CH_3_CN	80	46
8	1.2	-	CH_3_CN	80	58
9	1.2	-	CH_3_CN	100	60
10	1.2	-	CH_3_CN	120	66
11	1.2	-	Toluene	120	53
12	1.2	-	DMSO	120	31
13	1.2	-	THF	120	43
14 ^c^	1.2	-	CH_3_CN	120	63

^a^ Reaction conditions: d-lyxose tosylate (0.2 mmol, 1.0 equiv); reaction time: 5 h; oil bath heating; ^b^ isolated yield; ^c^ N_2_ atmosphere.

## Data Availability

The data are contained within the article and Supplementary Materials.

## Supplementary Materials

The Supporting Information is available free of charge at https://www.mdpi.com/article/10.3390/molecules29235709/s1, including the experimental methodologies, spectral analysis results, copies of the 1H and 13C NMR findings, as well as crystallographic information for the novel compounds. (PDF).
