# Supplementary material for: One-Pot Synthesis of Hydroxylated Alkaloids from Sugars via a Pictet–Spengler-Type Reaction
Source: Molecules. 2024 Dec 3;29(23):5709. doi: 10.3390/molecules29235709 (PMC11643748; doi:10.3390/molecules29235709)
Supplement: Supplementary file 1 [file molecules-29-05709-s001.zip › molecules-3305773-supplementary.pdf]

Supplementary Information

One pot synthesis of structurally diverse alkaloids using sugars via Pictet Spengler reaction

Likai Zhou, Na Ma, Jilai Wu*, Weilin Yang, Lijing Feng, Song Xie, Lili Wang and Hua Chen*

key Laboratory of Chemical Biology of Hebei Province, College of Chemistry and Material Science, Hebei University, Baoding Hebei, 071002, P. R. China; Email: hua-todd@163.com

Contents

1. **Experimental Section--S2**

2. **NMR of the compounds---S4**

3. **Crystal Information---S26**

4. **NMR spectra of the compunds--S29**

1. Experimental Section:

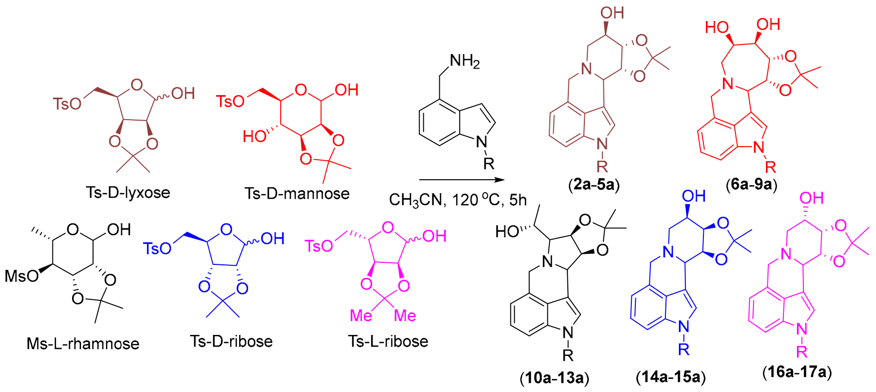

Scheme S1 Synthesis of the Indole alkaloids.

General experimental procedure: Ts-D-lyxose (69 mg, 0.2 mmol), indolemethylamines (1.2 equiv.) were added into a 20 mL flask, 2.0 mL CH_3CN as the solvent. Then the solution was stirred at the temperature of 120 °C under air atmosphere for 5h. Upon completion, the mixture was cooled to room temperature, the solvent was evaporated in vacuo. The crude product was purified by column chromatography (dichloromethane : methanol V/V = 30:1) to give **2a-5a** as a pale white solid. Under similar conditions, different Ts-D-mannose Ms-L-rhamnose Ts-D-ribose Ts-L-ribose were used as raw materials for the reaction, and the corresponding products were obtained respectively (**6a-17a**) (Scheme S1).

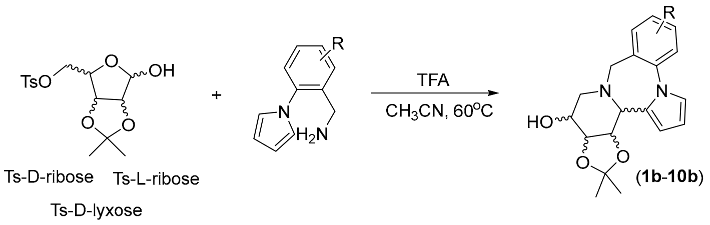

Scheme S2 Synthesis of the pyrrole alkaloids.

General experimental procedure: General experimental procedure: Ts-D-ribose (69 mg, 0.2 mmol), pyrrylyl-benzylamine (1.2 equiv.) and trifluoroacetic acid (0.2 equiv.) were added into a 20 mL flask, 2.0 mL CH_3CN as the solvent. Then the solution was stirred at the temperature of 60 °C under air atmosphere for 4 h. Upon completion, the mixture was

cooled to room temperature, the solvent was evaporated in vacuo. The crude product was purified by column chromatography (petroleum ether/ ethyl acetate *V/V* = 1:1) to give **1b-10b** as a pale white solid (Scheme S2).

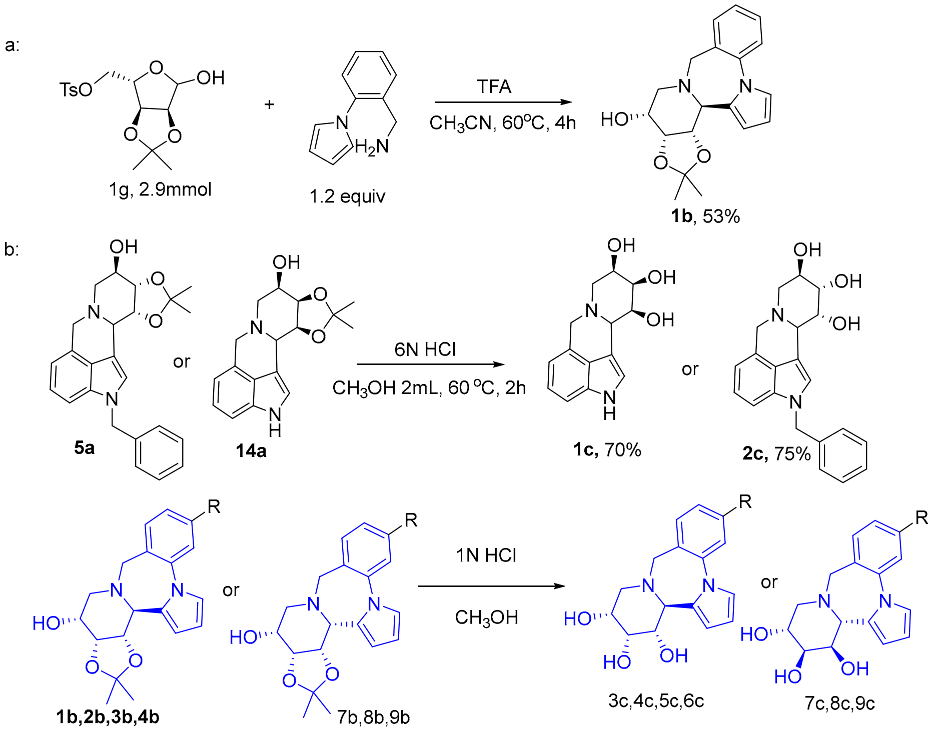

Scheme S3 Further transformations of alkaloids.

General experimental procedure: Ts-D-ribose (1g, 2.9 mmol), 2-(1-pyrrylyl)benzylamine (1.2 equiv.) and TFA (0.2 equiv.) were added into a 50 mL flask, 5.0 mL CH_3CN as the solvent. Then the solution was stirred at the temperature of 60 °C under air atmosphere for 4 h. Upon completion, the mixture was cooled to room temperature, the solvent was evaporated in vacuo. The crude product was purified by silica gel column chromatography (petroleum ether/ ethyl acetate *V/V* = 1:1) to obtain a light white solid **1b** 501 mg with a yield of 53% (Scheme S3 - a).

General experimental procedure: product **5a** (30 mg, 0.07 mmol) or **14a**, 6N HCl (5.0 equiv.) were added into a 20 mL flask, 2.0 mL methanol as the solvent. Then the solution was stirred at the temperature of 60 °C under air atmosphere for 2 h. Upon completion, the mixture was cooled to room temperature, and the solvent was evaporated in vacuo.

The crude product was purified by silica gel column chromatography (dichloromethane:methanol V/V = 15:1) to give **1c** or **2c** as a pale white solid. Under similar conditions, compound **1b-4b**, **7b-9b** reacts with 1N HCl (5.0 equiv.) to obtain compounds **3c-9c** (Scheme S3 - b).

2. NMR of the compounds

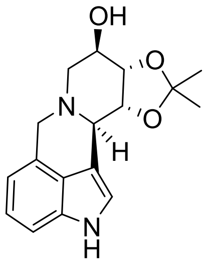

(3a*S*,4*R*,12c*R*)-2,2-dimethyl-3a,4,7,11,12b,12c-hexahydro-5*H*-[1,3]dioxolo[4',5':3,4]pyrido[1,2-*b*]pyrrolo[4,3,2-*de*]isoquinolin-4-ol (2a). Yellow solid, yield 66%, DCM:MeOH *V*/*V* = 30:1, m.p. 138.2 - 139.6 °C, $[\alpha]_D^{25}$ +36.0 (c 0.1, CH_3OH); 1H NMR (600 MHz, $CDCl_3$) δ 8.07 (s, 1H), 7.21 (d, *J* = 8.1 Hz, 1H), 7.15 (dd, *J* = 8.2, 6.9 Hz, 1H), 7.12 (t, *J* = 1.8 Hz, 1H), 6.86 (d, *J* = 6.9 Hz, 1H), 4.26 (ddd, *J* = 12.3, 7.8, 4.8 Hz, 2H), 4.10 (d, *J* = 2.7 Hz, 1H), 3.99 (d, *J* = 14.5 Hz, 1H), 3.90 (d, *J* = 14.4 Hz, 1H), 3.70 (dd, *J* = 7.8, 1.5 Hz, 1H), 3.09 – 2.89 (m, 3H), 1.59 (s, 3H), 1.46 (s, 3H). ^{13}C NMR (151 MHz, $CDCl_3$) δ 133.0, 128.0, 124.8, 123.0, 117.5, 114.1, 114.1, 109.8, 109.0, 78.5, 77.0, 76.6, 66.0, 60.7, 56.7, 55.2, 28.4, 26.7. MS (ESI): Calculated for $C_{17}H_{21}N_2O_3$ ($[M+H]^+$): 301.1547, found: 301.1552.

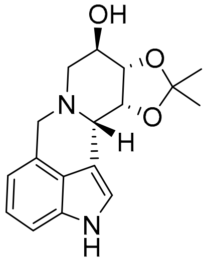

(3a*S*,4*R*,12b*S*,12c*R*)-2,2-dimethyl-3a,4,7,11,12b,12c-hexahydro-5*H*-[1,3]dioxolo[4',5':3,4]pyrido[1,2-*b*]pyrrolo[4,3,2-*de*]isoquinolin-4-ol (2a'). Yellow solid, yield 5%, DCM:MeOH *V*/*V* = 30:1, m.p. 128.9 - 129.1 °C, $[\alpha]_D^{25}$ +43.0 (c 0.1, CH_3OH); ^1H NMR (600 MHz, $CDCl_3$) δ 8.00 (s, 1H), 7.16 (d, *J* = 8.1 Hz, 1H), 7.11 (dd, *J* = 8.1, 7.0 Hz, 1H), 6.92 (t, *J* = 1.8 Hz, 1H), 6.83 (d, *J* = 6.9 Hz, 1H), 4.80 (dd, *J* = 5.1,

3.2 Hz, 1H), 4.13 (d, J = 14.5 Hz, 1H), 4.11 – 4.07 (m, 1H), 4.03 (dd, J = 7.8, 5.1 Hz, 1H), 3.91 (dd, J = 3.2, 1.5 Hz, 1H), 3.83 (d, J = 14.4 Hz, 1H), 3.22 (dd, J = 11.5, 4.7 Hz, 1H), 2.39 (t, J = 11.2 Hz, 1H), 1.50 (s, 3H), 1.46 (s, 3H). ^{13}C NMR (151 MHz, $CDCl_3$) δ 133.5, 128.4, 125.6, 123.0, 116.8, 114.3, 111.2, 109.6, 108.8, 80.5, 74.7, 70.7, 59.1, 58.0, 56.1, 28.6, 26.4. MS (ESI): Calculated for $C_{17}H_{21}N_2O_3$ ([M+H]$^+$): 301.1547, found: 301.1545.

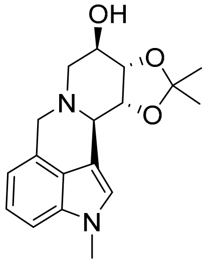

(3a*S*,4*R*,12c*R*)-2,2,11-trimethyl-3a,4,7,11,12b,12c-hexahydro-5*H*-[1,3]dioxolo[4',5':3,4]pyrido[1,2-*b*]pyrrolo[4,3,2-*de*]isoquinolin-4-ol (3a). Yellow solid, yield 56%, DCM:MeOH *V*/*V* = 30:1, m.p. 145.5 – 143.3 °C, $[\alpha]_D^{25}$ +27.0 (c 0.1, CH_3OH); ^{1}H NMR (400 MHz, $CDCl_3$) δ 7.15 (d, J = 6.4 Hz, 2H), 6.99 (d, J = 1.6 Hz, 1H), 6.82 (d, J = 5.9 Hz, 1H), 4.23 (d, J = 4.3 Hz, 2H), 4.08 (s, 1H), 4.00 – 3.93 (m, 1H), 3.88 (d, J = 14.5 Hz, 1H), 3.79 (d, J = 1.6 Hz, 3H), 3.70 – 3.65 (m, 1H), 3.03 – 2.94 (m, 2H), 1.58 (s, 3H), 1.45 (s, 3H). ^{13}C NMR (101 MHz, $CDCl_3$) δ 134.0, 128.1, 125.1, 122.6, 122.2, 113.5, 112.8, 109.8, 107.4, 78.6, 76.6, 66.0, 60.7, 56.8, 55.2, 32.9, 28.4, 26.7. MS (ESI): Calculated for $C_{18}H_{23}N_2O_3$ ([M+H]$^+$): 315.1703, found: 315.1700.

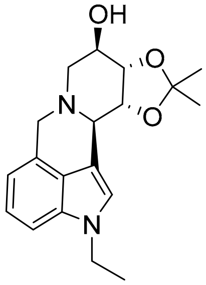

(3a*S*,4*R*,12c*R*)-11-ethyl-2,2-dimethyl-3a,4,7,11,12b,12c-hexahydro-5*H*-[1,3]dioxolo[4',5':3,4]pyrido[1,2-*b*]pyrrolo[4,3,2-*de*]isoquinolin-4-ol (4a). Yellow solid, yield 62%, DCM:MeOH *V*/*V* = 30:1, m.p. 114.2 - 115.4 °C, $[\alpha]_D^{25}$ +22.0 (c 0.1, CH_3OH); ^{1}H NMR (400 MHz, CD_3OD) δ 7.21 (d, J = 8.2 Hz, 1H), 7.16 – 7.09 (m, 2H), 6.80 (d, J = 6.9 Hz, 1H), 4.44 (dd, J = 6.4, 5.0 Hz, 1H), 4.21 (q, J = 7.2 Hz, 2H), 4.13 –

4.04 (m, 2H), 3.99 (dd, *J* = 13.7, 6.6 Hz, 3H), 2.78 (qd, *J* = 12.3, 4.3 Hz, 2H), 1.60 (s, 3H), 1.48 (s, 3H), 1.45 (t, *J* = 7.2 Hz, 3H). ^{13}C NMR (101 MHz, CD_3OD) δ 133.4, 127.4, 125.3, 122.2, 120.7, 113.3, 110.9, 108.9, 107.2, 77.5, 77.1, 67.1, 58.7, 55.1, 55.0, 47.7, 40.7, 27.3, 25.4, 14.9. MS (ESI): Calculated for $C_{19}H_{25}N_2O_3$ ($[M+H]^+$): 329.1860, found: 329.1864.

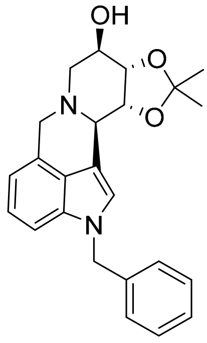

(3a*S*,4*R*,12c*R*)-11-benzyl-2,2-dimethyl-3a,4,7,11,12b,12c-hexahydro-5*H*-[1,3]dioxolo[4',5':3,4]pyrido[1,2-*b*]pyrrolo[4,3,2-*de*]isoquinolin-4-ol (5a). Yellow solid, yield 46%, DCM:MeOH *V*/*V* = 30:1, m.p. 123.1 - 123.7 ºC, $[\alpha]_D^{25}$ +63.0 (c 0.1, CH_3OH); ^1H NMR (400 MHz, CD_3OD) δ 7.33 – 7.25 (m, 3H), 7.19 – 7.07 (m, 5H), 6.81 (d, *J* = 6.7 Hz, 1H), 5.34 (s, 2H), 4.42 (dd, *J* = 6.6, 4.8 Hz, 1H), 4.15 – 3.95 (m, 5H), 2.87 – 2.71 (m, 2H), 1.59 (s, 3H), 1.46 (s, 3H). ^{13}C NMR (101 MHz, CD_3OD) δ 138.4, 134.0, 128.3, 127.5, 127.1, 126.7, 125.5, 122.4, 121.8, 113.6, 109.0, 107.7, 77.5, 77.1, 67.0, 58.8, 55.2, 54.9, 49.7, 27.3, 25.4. MS (ESI): Calculated for $C_{24}H_{27}N_2O_3$ ($[M+H]^+$): 391.2016, found: 391.2015.

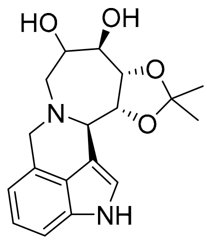

(3a*S*,4*R*,13c*R*)-2,2-dimethyl-3a,4,5,6,8,12,13b,13c-octahydro-[1,3]dioxolo[4',5':3,4]azepino[1,2-*b*]pyrrolo[4,3,2-*de*]isoquinoline-4,5-diol (6a). Yellow solid, yield 46%, DCM:MeOH *V*/*V* = 30:1, m.p. 136.2 - 137.5 ºC, $[\alpha]_D^{25}$ -39.0 (c 0.1, CH_3OH); ^1H NMR (400 MHz, $CDCl_3$) δ 8.14 (s, 1H), 7.29 (d, *J* = 9.1 Hz, 1H), 7.19 (t, *J* = 7.5 Hz, 1H), 7.16 (d, *J* = 1.8 Hz, 1H), 6.88 (d, *J* = 6.9 Hz, 1H), 4.53 (d, *J* = 16.0 Hz, 1H), 4.41 (d, *J* = 8.1 Hz, 1H), 4.35 (t, *J* = 9.3 Hz, 1H), 3.92 – 3.74 (m, 4H), 3.55 – 3.43

(m, 1H), 2.98 (dd, J = 10.7, 6.8 Hz, 1H), 2.77 (d, J = 15.1 Hz, 1H), 2.63 (dd, J = 15.2, 5.2 Hz, 1H), 1.57 (s, 6H). ^{13}C NMR (101 MHz, $CDCl_3$) δ 133.3, 127.4, 125.5, 123.1, 119.2, 114.9, 113.2, 110.6, 109.5, 81.4, 78.8, 77.3, 77.1, 75.8, 69.4, 58.6, 57.6, 51.2, 27.6, 27.2. MS (ESI): Calculated for $C_{18}H_{23}N_2O_4$ ($[M+H]^+$): 331.1652, found: 331.1653.

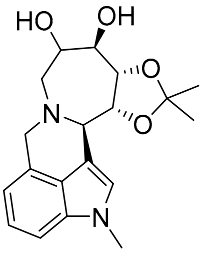

(3aS,4R,13cR)-2,2,12-trimethyl-3a,4,5,6,8,12,13b,13c-octahydro-[1,3]dioxolo[4',5':3,4]azepino[1,2-b]pyrrolo[4,3,2-de]isoquinoline-4,5-diol (7a). Yellow solid, yield 53%, DCM:MeOH *V*/*V* = 30:1, m.p. 134.2 - 135.3 ºC, $[\alpha]_D^{25}$ -41.0 (c 0.1, CH_3OH); ^{1}H NMR (400 MHz, $CDCl_3$) δ 7.23 – 7.16 (m, 2H), 6.99 (s, 1H), 6.85 (t, J = 3.9 Hz, 1H), 4.52 (d, J = 16.1 Hz, 1H), 4.40 (d, J = 8.1 Hz, 1H), 4.34 (dd, J = 10.8, 7.8 Hz, 1H), 3.84 (s, 3H), 3.78 (d, J = 7.8 Hz, 2H), 3.48 (s, 1H), 2.97 (s, 1H), 2.78 (d, J = 15.1 Hz, 1H), 2.62 (dd, J = 15.1, 5.2 Hz, 1H), 1.57 (d, J = 5.4 Hz, 7H). ^{13}C NMR (151 MHz, $CDCl_3$) δ 134.3, 127.4, 125.8, 123.7, 122.6, 114.2, 112.0, 110.5, 107.7, 81.5, 78.8, 75.8, 69.4, 64.5, 58.6, 57.6, 51.2, 32.9, 27.5, 27.1. MS (ESI): Calculated for $C_{19}H_{25}N_2O_4$ ($[M+H]^+$): 345.1809, found: 345.1810.

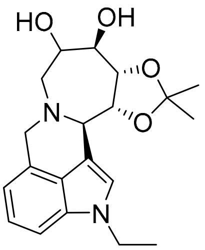

(3a*S*,4*R*,13c*R*)-12-ethyl-2,2-dimethyl-3a,4,5,6,8,12,13b,13c-octahydro-[1,3]dioxolo[4',5':3,4]azepino[1,2-*b*]pyrrolo[4,3,2-*de*]isoquinoline-4,5-diol (8a). Yellow solid, yield 52%, DCM:MeOH *V*/*V* = 30:1, m.p. 126.5 - 127.8 ºC, $[\alpha]_D^{25}$ -47.0 (c 0.1, CH_3OH); ^{1}H NMR (600 MHz, CD_3OD) δ 7.19 (t, J = 7.3 Hz, 1H), 7.13 – 7.05 (m, 2H), 6.77 (t, J = 6.6 Hz, 1H), 4.41 – 4.28 (m, 2H), 4.28 – 4.15 (m, 3H), 3.88 – 3.80 (m, 2H), 3.70 (q, J = 5.7 Hz, 1H), 3.57 (ddd, J = 9.1, 6.6, 4.2 Hz, 1H), 2.70 (dd, J = 15.0, 6.3 Hz, 1H), 2.65 – 2.59 (m, 1H), 1.52 (dd, J = 10.1, 6.4 Hz, 6H), 1.44 (q, J = 7.0 Hz, 3H).

^{13}C NMR (151 MHz, CD_3OD) δ 133.4, 127.9, 125.9, 122.0, 121.8, 113.4, 112.0, 109.6, 107.2, 81.4, 79.4, 74.7, 69.7, 59.6, 57.1, 47.7, 47.6, 40.7, 26.3, 26.0, 14.9. MS (ESI): Calculated for $C_{20}H_{27}N_2O_4$ ($[M+H]^+$): 359.1965, found: 359.1967.

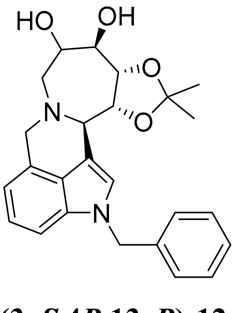

(3a*S*,4*R*,13c*R*)-12-benzyl-2,2-dimethyl-3a,4,5,6,8,12,13b,13c-octahydro-[1,3]dioxolo[4',5':3,4]azepino[1,2-*b*]pyrrolo[4,3,2-*de*]isoquinoline-4,5-diol (9a). Yellow solid, yield 60%, DCM:MeOH *V*/*V* = 30:1, m.p. 140.2 - 141.3 °C, $[\alpha]_D^{25}$ -36.0 (c 0.1, CH_3OH); ^1H NMR (600 MHz, CD_3OD) δ 7.17 – 7.08 (m, 3H), 7.05 – 7.02 (m, 2H), 6.98 (dd, *J* = 4.8, 3.5 Hz, 2H), 6.93 (dd, *J* = 8.2, 6.9 Hz, 1H), 6.65 (d, *J* = 6.8 Hz, 1H), 5.17 (d, *J* = 1.7 Hz, 2H), 4.23 (t, *J* = 9.1 Hz, 1H), 4.18 (d, *J* = 15.7 Hz, 1H), 4.11 (d, *J* = 8.3 Hz, 1H), 3.73 – 3.68 (m, 2H), 3.56 (t, *J* = 5.1 Hz, 1H), 3.42 (dd, *J* = 9.1, 4.4 Hz, 1H), 2.56 (d, *J* = 14.9 Hz, 1H), 2.48 (dd, *J* = 15.0, 5.9 Hz, 1H), 1.37 (d, *J* = 3.6 Hz, 6H). ^{13}C NMR (151 MHz, CD_3OD) δ 138.4, 134.0, 128.2, 128.0, 127.1, 127.1, 126.7, 126.0, 123.0, 122.3, 113.8, 112.5, 109.6, 107.8, 81.3, 79.4, 74.6, 69.7, 59.5, 57.1, 52.3, 49.7, 26.3, 26.0. MS (ESI): Calculated for $C_{25}H_{29}N_2O_4$ ($[M+H]^+$): 421.2122, found: 421.2125.

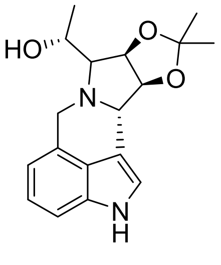

(1*R*)-1-((8a*R*,11a*S*)-10,10-dimethyl-2,6,8,8a,11a,11b-hexahydro-[1,3]dioxolo[4',5':3,4]pyrrolo[1,2-*b*]pyrrolo[4,3,2-*de*]isoquinolin-8-yl)ethan-1-ol (10a). Yellow solid, yield 70%, DCM:MeOH *V*/*V* = 30:1, m.p. 124.6 - 125.6 °C, $[\alpha]_D^{25}$ -26.0 (c 0.1, CH_3OH); ^1H NMR (600 MHz, $CDCl_3$) δ 7.14 (dd, *J* = 8.2, 3.9 Hz, 1H), 7.10 – 7.03 (m, 2H), 6.77 (t, *J* = 5.4 Hz, 1H), 4.23 – 4.15 (m, 3H), 3.82 (s, 1H), 3.66 (dd, *J* = 8.8, 4.0 Hz, 1H), 3.57 (dd, *J* = 14.2, 4.1 Hz, 1H), 3.21 – 3.16 (m, 2H), 2.99 (q, *J* = 6.1 Hz, 1H), 1.52 (d, *J* = 4.0 Hz, 3H), 1.38 (dd, *J* = 10.8, 4.3 Hz, 6H). ^{13}C NMR (151 MHz, $CDCl_3$) δ

132.7, 127.4, 124.7, 122.6, 117.7, 113.8, 109.6, 109.1, 78.3, 78.3, 77.1, 76.6, 60.9, 57.7, 50.4, 28.2, 26.4, 16.7. MS (ESI): Calculated for $C_{18}H_{23}N_2O_3$ ($[M+H]^+$): 315.1703, found: 315.1700.

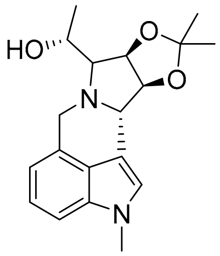

(1*R*)-1-((8a*R*,11a*S*)-2,10,10-trimethyl-2,6,8,8a,11a,11b-hexahydro-[1,3]dioxolo[4',5':3,4]pyrrolo[1,2-*b*]pyrrolo[4,3,2-*de*]isoquinolin-8-yl)ethan-1-ol (11a). Yellow solid, yield 62%, DCM:MeOH *V*/*V* = 30:1, m.p. 127.3 - 128.1 °C, $[\alpha]_D^{25}$ -37.0 (c 0.1, CH_3OH); ^1H NMR (600 MHz, $CDCl_3$) δ 7.18 – 7.12 (m, 2H), 6.99 (d, *J* = 1.4 Hz, 1H), 6.82 (d, *J* = 6.6 Hz, 1H), 4.28 (dd, *J* = 4.9, 2.8 Hz, 1H), 4.23 (dd, *J* = 8.6, 4.9 Hz, 1H), 4.19 (d, *J* = 14.2 Hz, 1H), 3.86 (s, 1H), 3.79 (s, 3H), 3.70 (dd, *J* = 8.6, 1.5 Hz, 1H), 3.63 (d, *J* = 14.2 Hz, 1H), 3.06 (dtd, *J* = 8.4, 6.7, 1.5 Hz, 1H), 1.58 (s, 3H), 1.45 (s, 3H), 1.44 (d, *J* = 6.8 Hz, 3H). ^{13}C NMR (151 MHz, $CDCl_3$) δ 133.8, 128.1, 125.0, 122.6, 122.1, 113.6, 109.9, 107.3, 78.9, 77.0, 76.6, 70.8, 61.3, 57.7, 50.2, 32.9, 28.3, 26.7, 17.0. MS (ESI): Calculated for $C_{19}H_{25}N_2O_3$ ($[M+H]^+$): 329.1860, found: 329.1863.

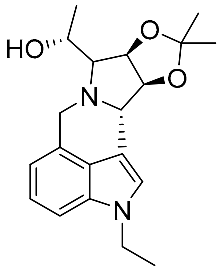

(1*R*)-1-((8a*R*,11a*S*)-2-ethyl-10,10-dimethyl-2,6,8,8a,11a,11b-hexahydro-[1,3]dioxolo[4',5':3,4]pyrrolo[1,2-*b*]pyrrolo[4,3,2-*de*]isoquinolin-8-yl)ethan-1-ol (12a). Yellow solid, yield 61%, DCM:MeOH *V*/*V* = 30:1, m.p. 119.2 - 120.2 °C, $[\alpha]_D^{25}$ -43.0 (c 0.1, CH_3OH); ^1H NMR (400 MHz, CD_3OD) δ 7.20 (d, *J* = 8.2 Hz, 1H), 7.14 – 7.07 (m, 2H), 6.81 (d, *J* = 6.9 Hz, 1H), 4.33 – 4.27 (m, 2H), 4.25 (q, *J* = 3.2, 2.7 Hz, 1H), 4.21 (t, *J* = 7.2 Hz, 2H), 3.96 (t, *J* = 2.9 Hz, 1H), 3.80 (dd, *J* = 8.8, 1.3 Hz, 1H), 3.68 (d, *J* = 14.5 Hz, 1H), 3.02 (qd, *J* = 6.8, 2.6 Hz, 1H), 1.61 (s, 3H), 1.47 (s, 3H), 1.45 – 1.39 (m, 6H). ^{13}C NMR (101 MHz, CD_3OD) δ 133.1, 128.1, 125.3, 122.0, 120.4, 113.0, 108.9, 107.0,

77.5, 76.9, 70.1, 59.5, 57.9, 49.9, 47.6, 40.7, 27.3, 25.2, 15.3, 14.9. MS (ESI): Calculated for $C_{20}H_{27}N_2O_3$ ($[M+H]^+$): 343.2016, found: 343.2015.

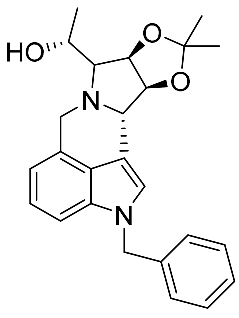

(1*R*)-1-((8a*R*,11a*S*)-2-benzyl-10,10-dimethyl-2,6,8,8a,11a,11b-hexahydro-[1,3]dioxolo[4',5':3,4]pyrrolo[1,2-*b*]pyrrolo[4,3,2-*de*]isoquinolin-8-yl)ethan-1-ol (13a). Yellow solid, yield 57%, DCM:MeOH *V*/*V* = 30:1, m.p. 120.9 - 121.6 °C, $[\alpha]_D^{25}$ -32.0 (c 0.1, CH_3OH); ^1H NMR (600 MHz, $CDCl_3$) δ 7.23 – 7.13 (m, 3H), 7.08 – 6.94 (m, 5H), 6.74 (d, *J* = 2.9 Hz, 1H), 5.25 – 5.20 (m, 2H), 4.22 (tdd, *J* = 13.5, 6.6, 2.7 Hz, 2H), 4.14 (dd, *J* = 14.3, 2.6 Hz, 1H), 3.83 – 3.78 (m, 1H), 3.58 – 3.53 (m, 1H), 3.31 – 3.24 (m, 1H), 2.97 (ddt, *J* = 11.8, 6.6, 2.7 Hz, 1H), 1.52 – 1.45 (m, 3H), 1.39 – 1.32 (m, 6H). ^{13}C NMR (151 MHz, $CDCl_3$) δ 141.8, 137.4, 132.5, 131.7, 131.4, 130.7, 129.2, 126.6, 126.6, 125.7, 125.7, 117.8, 117.5, 113.6, 111.8, 82.2, 80.5, 74.5, 64.6, 61.6, 54.3, 54.2, 53.3, 53.2, 53.1, 53.0, 53.0, 32.1, 32.1, 30.3, 30.3, 20.6, 20.5. MS (ESI): Calculated for $C_{25}H_{29}N_2O_3$ ($[M+H]^+$): 405.2173, found: 405.2177.

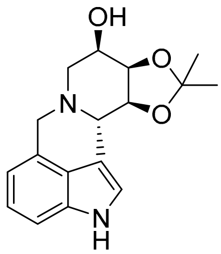

(3a*R*,4*R*,12b*S*,12c*S*)-2,2-dimethyl-3a,4,7,11,12b,12c-hexahydro-5*H*-[1,3]dioxolo[4',5':3,4]pyrido[1,2-*b*]pyrrolo[4,3,2-*de*]isoquinolin-4-ol (14a). Yellow solid, yield 51%, DCM:MeOH *V*/*V* = 30:1, m.p. 121.4 - 122.5 °C, $[\alpha]_D^{25}$ -61.0 (c 0.1, CH_3OH); ^1H NMR (600 MHz, $CDCl_3$) δ 7.08 (dd, *J* = 8.2, 2.2 Hz, 1H), 7.05 – 6.98 (m, 2H), 6.72 (dd, *J* = 7.2, 2.0 Hz, 1H), 4.35 (dd, *J* = 4.4, 1.8 Hz, 1H), 4.13 (ddd, *J* = 8.4, 4.6, 2.0 Hz, 1H), 4.04 – 3.98 (m, 1H), 3.93 (dd, *J* = 14.6, 2.0 Hz, 1H), 3.81 – 3.76 (m, 1H), 3.62 – 3.57 (m, 1H), 2.97 (ddd, *J* = 11.2, 5.4, 2.0 Hz, 1H), 2.64 – 2.59 (m, 1H), 1.52 (d, *J*

= 2.0 Hz, 3H), 1.38 (d, J = 2.0 Hz, 3H).^{13}C NMR (151 MHz, $CDCl_3$) δ 132.9, 127.3, 124.9, 122.7, 117.5, 113.8, 113.0, 110.0, 109.1, 79.7, 75.5, 65.6, 59.5, 56.0, 55.4, 53.4, 28.2, 26.5. MS (ESI): Calculated for $C_{17}H_{21}N_2O_3$ ($[M+H]^+$): 301.1547, found: 301.1548.

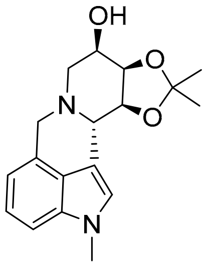

(3a*R*,4*R*,12c*S*)-2,2,11-trimethyl-3a,4,7,11,12b,12c-hexahydro-5*H*-[1,3]dioxolo[4',5':3,4]pyrido[1,2-*b*]pyrrolo[4,3,2-*de*]isoquinolin-4-ol (15a). Yellow solid, yield 58%, DCM:MeOH V/V = 30:1, m.p. 136.6 - 137.2 °C, $[\alpha]_D^{25}$ -70.0 (c 0.1, CH_3OH); ^{1}H NMR (600 MHz, CD_3OD) δ 7.18 (d, J = 8.2 Hz, 1H), 7.12 (dd, J = 8.2, 6.9 Hz, 1H), 7.04 (d, J = 1.4 Hz, 1H), 6.81 (d, J = 6.9 Hz, 1H), 4.50 (t, J = 4.2 Hz, 1H), 4.19 – 4.08 (m, 2H), 4.04 (d, J = 14.4 Hz, 1H), 3.80 (s, 4H), 3.65 (dd, J = 8.7, 1.5 Hz, 1H), 3.05 (dd, J = 11.0, 5.4 Hz, 1H), 2.75 (t, J = 11.1 Hz, 1H), 1.62 (s, 3H), 1.48 (s, 3H). ^{13}C NMR (151 MHz, CD_3OD) δ 134.2, 127.4, 125.3, 122.1, 121.9, 112.9, 112.1, 109.8, 107.1, 79.8, 75.7, 65.1, 59.7, 55.6, 55.0, 47.6, 31.5, 27.4, 25.4. MS (ESI): Calculated for $C_{18}H_{23}N_2O_3$ ($[M+H]^+$): 315.1703, found: 315.1707.

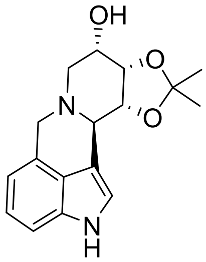

(3a*S*,4*S*,12c*R*)-2,2-dimethyl-3a,4,7,11,12b,12c-hexahydro-5*H*-[1,3]dioxolo[4',5':3,4]pyrido[1,2-*b*]pyrrolo[4,3,2-*de*]isoquinolin-4-ol (16a). Yellow solid, yield 53%, DCM:MeOH V/V = 30:1, m.p. 135.3 - 136.7 °C, $[\alpha]_D^{25}$ +63.0 (c 0.1, CH_3OH); ^{1}H NMR (600 MHz, $CDCl_3$) δ 7.07 (d, J = 8.1 Hz, 1H), 7.04 – 6.92 (m, 2H), 6.68 (d, J = 7.1 Hz, 1H), 4.35 (q, J = 5.0, 4.2 Hz, 1H), 4.10 (q, J = 4.8, 4.2 Hz, 1H), 4.02 – 3.92 (m, 2H), 3.75 (d, J = 14.2 Hz, 1H), 3.56 (d, J = 8.6 Hz, 1H), 2.93 (dd, J = 10.9, 5.4 Hz, 1H), 2.62 (dd, J = 12.0, 10.2 Hz, 1H), 1.49 (s, 3H), 1.35 (s, 3H). ^{13}C NMR (151 MHz, $CDCl_3$) δ 132.8, 127.0, 124.7, 122.3, 117.5, 113.3, 112.5, 109.8, 109.0, 79.6, 77.0, 75.4,

65.1, 59.4, 55.6, 55.2, 48.6, 27.9, 26.2. MS (ESI): Calculated for $C_{17}H_{21}N_2O_3$ ($[M+H]^+$): 301.1547, found: 301.1550.

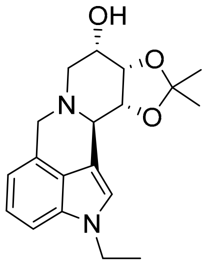

(3a*S*,4*S*,12c*R*)-11-ethyl-2,2-dimethyl-3a,4,7,11,12b,12c-hexahydro-5*H*-[1,3]dioxolo[4',5':3,4]pyrido[1,2-*b*]pyrrolo[4,3,2-*de*]isoquinolin-4-ol (17a). Yellow solid, yield 61%, DCM:MeOH *V*/*V* = 30:1, m.p. 133.3 - 135.0 °C, $[\alpha]_D^{25}$ +57.0 (c 0.1, CH_3OH); ^{1}H NMR (600 MHz, CD_3OD) δ 7.18 (d, *J* = 1.4 Hz, 1H), 7.08 (d, *J* = 8.2 Hz, 1H), 6.99 (dd, *J* = 8.2, 7.0 Hz, 1H), 6.67 (d, *J* = 7.0 Hz, 1H), 4.39 (t, *J* = 4.2 Hz, 1H), 4.23 – 4.19 (m, 1H), 4.08 (q, *J* = 7.3 Hz, 2H), 3.89 (d, *J* = 14.5 Hz, 1H), 3.73 – 3.68 (m, 2H), 3.63 (dd, *J* = 10.0, 1.4 Hz, 1H), 2.99 (dd, *J* = 11.7, 6.9 Hz, 1H), 2.51 (dd, *J* = 11.7, 10.1 Hz, 1H), 1.48 (s, 3H), 1.34 – 1.29 (m, 6H). ^{13}C NMR (151 MHz, CD_3OD) δ 132.9, 127.3, 125.5, 121.6, 112.5, 111.4, 109.2, 106.9, 76.0, 71.6, 71.2, 58.5, 56.0, 55.0, 47.4, 40.5, 27.2, 25.0, 14.6. MS (ESI): Calculated for $C_{19}H_{25}N_2O_3$ ($[M+H]^+$): 329.1860, found: 329.1857.

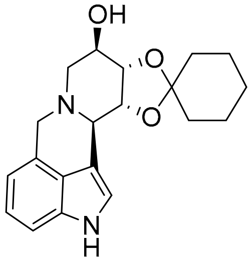

(3a'*S*,4'*R*,12b'*R*,12c'*R*)-3a',4',7',11',12b',12c'-hexahydro-5'*H*-spiro[cyclohexane-1,2'-[1,3]dioxolo[4',5':3,4]pyrido[1,2-*b*]pyrrolo[4,3,2-de]isoquinolin]-4'-ol (18a). Yellow solid, yield 63%, DCM:MeOH *V*/*V* = 30:1, m.p. 136.7 - 137.4 °C, $[\alpha]_D^{25}$ +68.0 (c 0.1, CH_3OH); ^{1}H NMR (400 MHz, $CDCl_3$) δ 8.19 – 8.11 (m, 1H), 7.24 (d, *J* = 8.1 Hz, 1H), 7.21 – 7.17 (m, 1H), 7.15 (q, *J* = 1.9 Hz, 1H), 6.89 (d, *J* = 6.8 Hz, 1H), 4.27 – 4.20 (m, 2H), 4.18 – 4.12 (m, 1H), 4.01 (d, *J* = 14.6 Hz, 1H), 3.90 (d, *J* = 14.5 Hz, 1H), 3.71 (dd, *J* = 7.6, 1.5 Hz, 1H), 3.19 (d, *J* = 10.0 Hz, 1H), 3.07 – 2.93 (m, 2H), 1.83 (t, *J* = 6.1 Hz, 2H), 1.71 (t, *J* = 5.6 Hz, 6H), 1.48 (q, *J* = 5.7 Hz, 2H). ^{13}C NMR (101 MHz, $CDCl_3$) δ

133.0, 128.0, 124.9, 123.0, 117.6, 114.1, 110.4, 109.1, 77.8, 77.4, 76.2, 66.1, 60.9, 56.8, 55.2, 38.3, 35.9, 25.2, 24.3, 23.9. MS (ESI): Calculated for $C_{20}H_{25}N_2O_3$ ($[M+H]^+$): 341.1860, found: 341.1863.

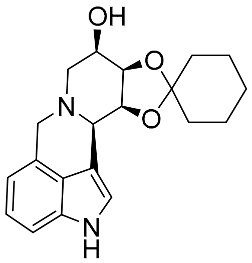

(3a'*R*,4'*R*,12b'*R*,12c'*S*)-3a',4',7',11',12b',12c'-hexahydro-5'*H*-spiro[cyclohexane-1,2'-[1,3]dioxolo[4',5':3,4]pyrido[1,2-*b*]pyrrolo[4,3,2-de]isoquinolin]-4'-ol (**19a**). Yellow solid, yield 60%, DCM:MeOH *V*/*V* = 30:1, m.p. 127.0 - 128.5 °C, $[\alpha]_D^{25}$ -57.0 (c 0.1, CH_3OH); ^{1}H NMR (400 MHz, CD_3OD) δ 7.21 (d, *J* = 8.2 Hz, 1H), 7.16 (d, *J* = 1.4 Hz, 1H), 7.08 (dd, *J* = 8.2, 7.0 Hz, 1H), 6.80 (d, *J* = 7.0 Hz, 1H), 4.50 (t, *J* = 4.2 Hz, 1H), 4.19 (dd, *J* = 8.7, 4.3 Hz, 1H), 4.12 (ddd, *J* = 11.1, 5.4, 4.0 Hz, 1H), 4.06 (d, *J* = 14.4 Hz, 1H), 3.85 (d, *J* = 14.4 Hz, 1H), 3.68 (dd, *J* = 8.8, 1.5 Hz, 1H), 3.08 (dd, *J* = 11.0, 5.4 Hz, 1H), 2.78 (t, *J* = 11.0 Hz, 1H), 1.86 (q, *J* = 6.2, 5.7 Hz, 2H), 1.81 – 1.68 (m, 6H), 1.49 (d, *J* = 5.8 Hz, 2H). ^{13}C NMR (151 MHz, CD_3OD) δ 134.7, 128.4, 126.4, 123.4, 118.9, 114.3, 113.9, 111.8, 110.3, 80.8, 76.7, 66.7, 61.6, 57.1, 56.7, 39.4, 36.9, 26.3, 25.3, 24.8. MS (ESI): Calculated for $C_{20}H_{25}N_2O_3$ ($[M+H]^+$): 341.1860, found: 341.1864.

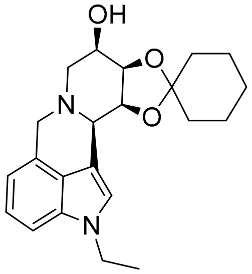

(3a'*R*,4'*R*,12b'*R*,12c'*S*)-11'-ethyl-3a',4',7',11',12b',12c'-hexahydro-5'*H*-spiro[cyclohexane-1,2'-[1,3]dioxolo[4',5':3,4]pyrido[1,2-*b*]pyrrolo[4,3,2-de]isoquinolin]-4'-ol (20a). Yellow solid, yield 67%, DCM:MeOH *V*/*V* = 30:1, m.p. 120.4 - 122.5 °C, $[\alpha]_D^{25}$ -45.0 (c 0.1, CH_3OH); ^{1}H NMR (400 MHz, $CDCl_3$) δ 7.18 – 7.13 (m, 2H), 7.09 (d, *J* = 1.4 Hz, 1H), 6.84 (dd, *J* = 5.5, 2.2 Hz, 1H), 4.49 (t, *J* = 4.4 Hz, 1H), 4.25 (dd, *J* = 8.2, 4.6 Hz, 1H), 4.18 (q, *J* = 7.3 Hz, 3H), 4.05 (d, *J* = 14.5 Hz, 1H), 3.89 (d,

J = 14.5 Hz, 1H), 3.69 (dd, *J* = 8.2, 1.4 Hz, 1H), 3.14 (dd, *J* = 11.1, 5.4 Hz, 1H), 2.68 (t, *J* = 10.7 Hz, 1H), 1.89 (td, *J* = 6.7, 3.7 Hz, 2H), 1.73 (ddd, *J* = 17.3, 7.6, 3.4 Hz, 6H), 1.49 (d, *J* = 7.3 Hz, 5H). ^{13}C NMR (101 MHz, $CDCl_3$) δ 133.2, 128.2, 125.4, 122.4, 120.5, 113.5, 112.7, 110.5, 107.4, 79.7, 75.0, 66.5, 59.8, 56.8, 55.3, 41.3, 38.1, 35.9, 25.2, 24.3, 23.9, 16.0. MS (ESI): Calculated for $C_{22}H_{29}N_2O_3$ ($[M+H]^+$): 369.2173, found: 369.2167.

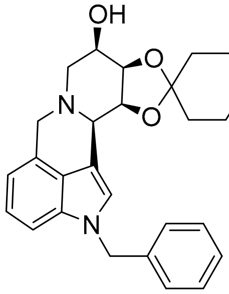

(3a'*R*,4'*R*,12b'*R*,12c'*S*)-11'-benzyl-3a',4',7',11',12b',12c'-hexahydro-5'*H*-spiro[cyclohexane-1,2'-[1,3]dioxolo[4',5':3,4]pyrido[1,2-*b*]pyrrolo[4,3,2-de]isoquinolin]-4'-ol (21a). Yellow solid, yield 64%, DCM:MeOH *V*/*V* = 30:1, m.p. 135.1 - 136.2°C, $[\alpha]_D^{25}$ -48.0 (c 0.1, CH_3OH); ^{1}H NMR (400 MHz, $CDCl_3$) δ 7.32 (q, *J* = 4.7, 2.7 Hz, 3H), 7.23 – 7.17 (m, 2H), 7.15 (d, *J* = 5.5 Hz, 2H), 6.90 – 6.82 (m, 2H), 5.34 (s, 2H), 4.77 (t, *J* = 4.6 Hz, 1H), 4.19 (t, *J* = 5.2 Hz, 1H), 4.08 (d, *J* = 14.6 Hz, 1H), 3.99 – 3.87 (m, 3H), 3.28 (dd, *J* = 12.3, 4.1 Hz, 1H), 2.62 (d, *J* = 12.2 Hz, 1H), 1.96 (d, *J* = 6.2 Hz, 1H), 1.83 (dt, *J* = 9.6, 6.0 Hz, 2H), 1.63 (td, *J* = 12.1, 11.4, 4.1 Hz, 5H), 1.49 – 1.43 (m, 2H). ^{13}C NMR (151 MHz, $CDCl_3$) δ 137.8, 134.1, 128.8, 128.7, 127.6, 127.0, 126.1, 122.6, 121.2, 113.8, 110.8, 109.9, 107.7, 74.3, 72.7, 65.9, 59.1, 58.0, 55.9, 50.3, 35.1, 34.8, 25.3, 24.2, 24.0. MS (ESI): Calculated for $C_{27}H_{31}N_2O_3$ ($[M+H]^+$): 431.2329, found: 431.2331.

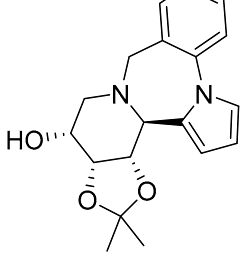

(3aR,4R,15bS,15cS)-2,2-dimethyl-3a,4,15b,15c-tetrahydro-5H,7H-[1,3]dioxolo[4',5':3,4]pyrido[1,2-a]benzo[e]pyrrolo[2,1-c][1,4]diazepin-4-ol. (1b). white solid, yield 71%, petroleum ether/ ethyl acetate *V*/*V* = 1:1, m.p. 179.4-181.1 °C.

[α]$_D^{25}$ -121.0 (*c* 0.1, CH_3OH); ^{1}H NMR (600 MHz, Chloroform-*d*) δ 7.4 (p, *J* = 3.7 Hz, 1H), 7.4 (d, *J* = 7.7 Hz, 1H), 7.3 (d, *J* = 4.4 Hz, 2H), 7.0 (s, 1H), 6.3 (d, *J* = 2.3 Hz, 2H), 4.6 (dd, *J* = 9.4, 5.0 Hz, 1H), 4.5 (t, *J* = 4.8 Hz, 1H), 4.1 (dt, *J* = 10.3, 5.2 Hz, 1H), 3.8 (d, *J* = 14.2 Hz, 1H), 3.4 (d, *J* = 14.2 Hz, 1H), 3.0 (d, *J* = 9.3 Hz, 1H), 3.0 (dd, *J* = 10.7, 5.8 Hz, 1H), 2.6 (t, *J* = 10.2 Hz, 1H), 1.3 (s, 3H), 1.2 (s, 3H). ^{13}C NMR (151 MHz, Chloroform-*d*) δ 130.6, 130.4, 129.3, 128.3, 126.4, 121.9, 120.9, 109.4, 109.3, 106.7, 75.3, 74.8, 67.0, 58.3, 56.5, 55.6, 28.1, 26.1. MS (ESI): Calculated for $C_{19}H_{23}N_2O_3$ ($[M+H]^+$): 327.1703, found: 327.1705.

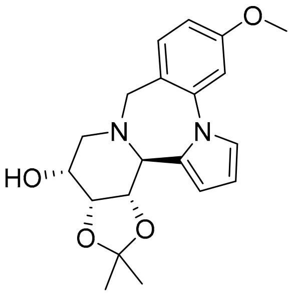

(3aR,4R,15bS,15cS)-10-methoxy-2,2-dimethyl-3a,4,15b,15c-tetrahydro-5H,7H-[1,3]dioxolo[4',5':3,4]pyrido[1,2-a]benzo[e]pyrrolo[2,1-c][1,4]diazepin-4-ol (2b). white solid, yield 68%, petroleum ether/ ethyl acetate *V/V* = 1:1, m.p. 165.4-166.6 ºC. [α]$_D^{25}$ -114.0 (*c* 0.1, CH_3OH); ^{1}H NMR (400 MHz, Chloroform-*d*) δ 7.3 (d, *J* = 8.6 Hz, 1H), 7.0 – 6.9 (m, 2H), 6.8 (d, *J* = 2.8 Hz, 1H), 6.3 (d, *J* = 2.3 Hz, 2H), 4.6 (dd, *J* = 9.3, 5.0 Hz, 1H), 4.5 (t, *J* = 4.7 Hz, 1H), 4.1 (dt, *J* = 10.1, 5.3 Hz, 1H), 3.9 (s, 3H), 3.8 (d, *J* = 14.2 Hz, 1H), 3.4 (d, *J* = 14.1 Hz, 1H), 3.1 – 2.9 (m, 2H), 2.6 (t, *J* = 10.1 Hz, 1H), 1.4 (s, 3H), 1.3 (s, 3H). ^{13}C NMR (101 MHz, Chloroform-*d*) δ 157.9, 122.9, 120.9, 115.7, 114.0, 109.4, 109.0, 106.3, 75.2, 74.8, 67.0, 58.3, 56.6, 55.7, 55.6, 28.1, 26.1. MS (ESI): Calculated for $C_{20}H_{25}N_2O_4$ ($[M+H]^+$): 357.1809, found: 357.1811.

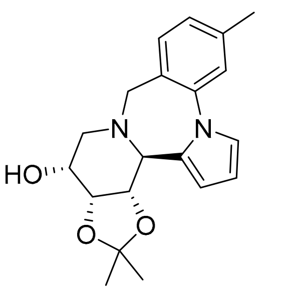

(3aR,4R,15bS,15cS)-2,2,10-trimethyl-3a,4,15b,15c-tetrahydro-5H,7H-[1,3]dioxolo[4',5':3,4]pyrido[1,2-a]benzo[e]pyrrolo[2,1-c][1,4]diazepin-4-ol (3b).

Light yellow solid, yield 67%, petroleum ether/ ethyl acetate *V/V* = 1:1, m.p. 147.2-148.6 °C. $[\alpha]_D^{25}$ -99.0 (*c* 0.1, CH_3OH); ^1H NMR (400 MHz, Chloroform-*d*) δ 7.3 (d, *J* = 8.2 Hz, 1H), 7.3 (dd, *J* = 8.1, 1.9 Hz, 1H), 7.1 (d, *J* = 1.9 Hz, 1H), 7.0 (t, *J* = 2.2 Hz, 1H), 6.3 (d, *J* = 2.3 Hz, 2H), 4.6 (dd, *J* = 9.3, 5.0 Hz, 1H), 4.5 (t, *J* = 4.7 Hz, 1H), 4.2 (dd, *J* = 6.3, 3.3 Hz, 1H), 3.8 (d, *J* = 14.1 Hz, 1H), 3.4 (d, *J* = 14.1 Hz, 1H), 3.0 (d, *J* = 9.3 Hz, 1H), 3.0 (dd, *J* = 10.7, 5.9 Hz, 1H), 2.7 (dd, *J* = 10.7, 9.6 Hz, 1H), 2.4 (s, 3H), 1.4 (s, 3H), 1.3 (s, 3H). ^{13}C NMR (101 MHz, Chloroform-*d*) δ 137.5, 136.2, 130.9, 130.6, 129.7, 128.0, 121.6, 120.8, 109.3, 109.1, 106.4, 75.3, 74.8, 67.1, 58.3, 56.5, 55.7, 28.1, 26.1, 21.1. MS (ESI): Calculated for $C_{20}H_{25}N_2O_3$ ($[M+H]^+$): 341.1860, found: 341.1862.

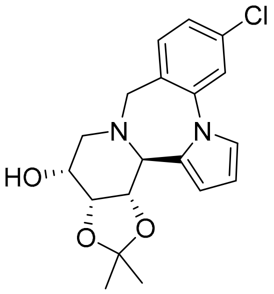

(3aR,4R,15bS,15cS)-10-chloro-2,2-dimethyl-3a,4,15b,15c-tetrahydro-5H,7H-[1,3]dioxolo[4',5':3,4]pyrido[1,2-a]benzo[e]pyrrolo[2,1-c][1,4]diazepin-4-ol (4b). white solid, yield 67%, petroleum ether/ ethyl acetate *V/V* = 1:1, m.p. 184.4-186.8 °C. $[\alpha]_D^{25}$ -84.0 (*c* 0.1, CH_3OH); ^1H NMR (400 MHz, Chloroform-*d*) δ 7.4 (dd, *J* = 8.4, 2.3 Hz, 1H), 7.3 (d, *J* = 8.4 Hz, 1H), 7.3 (d, *J* = 2.3 Hz, 1H), 6.9 (t, *J* = 2.2 Hz, 1H), 6.3 (d, *J* = 2.4 Hz, 2H), 4.6 (dd, *J* = 9.3, 5.0 Hz, 1H), 4.5 (t, *J* = 4.7 Hz, 1H), 4.1 (s, 1H), 3.8 (d, *J* = 14.2 Hz, 1H), 3.4 (d, *J* = 14.3 Hz, 1H), 3.0 – 2.9 (m, 2H), 2.6 (dd, *J* = 10.6, 9.6 Hz, 1H), 1.4 (s, 3H), 1.3 (s, 3H). ^{13}C NMR (101 MHz, Chloroform-*d*) δ 138.5, 131.8, 130.7, 130.2, 130.1, 129.3, 123.1, 120.9, 109.7, 109.5, 107.1, 74.8, 67.0, 58.4, 56.2, 55.7, 28.1, 26.1. MS (ESI): Calculated for $C_{19}H_{22}ClN_2O_3$ ($[M+H]^+$): 361.1313, found: 361.1315.

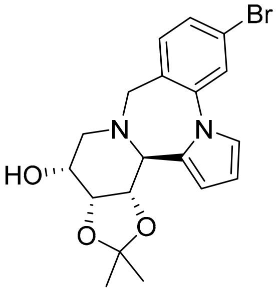

(3aR,4R,15bS,15cS)-10-bromo-2,2-dimethyl-3a,4,15b,15c-tetrahydro-5H,7H-[1,3]dioxolo[4',5':3,4]pyrido[1,2-a]benzo[e]pyrrolo[2,1-c][1,4]diazepin-4-ol (5b). white solid, yield 67%, petroleum ether/ ethyl acetate *V/V* = 1:1, m.p. 178.5-180.2 °C. $[\alpha]_D^{25}$ -91.0 (*c* 0.1, CH_3OH); ^{1}H NMR (400 MHz, Chloroform-*d*) δ 7.4 (dd, *J* = 8.5, 2.3 Hz, 1H), 7.3 (d, *J* = 8.4 Hz, 1H), 7.3 (d, *J* = 2.3 Hz, 1H), 6.9 (t, *J* = 2.3 Hz, 1H), 6.3 (d, *J* = 2.2 Hz, 2H), 4.6 (dd, *J* = 9.3, 5.0 Hz, 1H), 4.5 (t, *J* = 4.7 Hz, 1H), 4.2 – 4.1 (m, 1H), 3.8 (d, *J* = 14.2 Hz, 1H), 3.4 (d, *J* = 14.2 Hz, 1H), 3.0 (d, *J* = 9.6 Hz, 1H), 3.0 – 2.9 (m, 1H), 2.6 (t, *J* = 10.1 Hz, 1H), 1.3 (s, 3H), 1.3 (s, 3H). ^{13}C NMR (101 MHz, Chloroform-*d*) δ 138.6, 131.9, 130.8, 130.3, 130.2, 129.4, 123.2, 121.0, 109.8, 109.6, 107.2, 74.9, 67.1, 58.5, 56.3, 55.8, 28.2, 26.2. MS (ESI): Calculated for $C_{19}H_{22}BrN_2O_3$ ($[M+H]^+$): 405.0808, found: 405.0810.

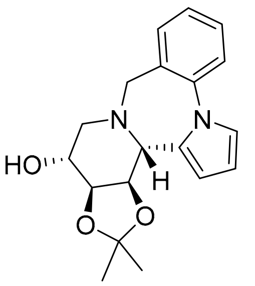

(3aS,4R,15bR,15cR)-2,2-dimethyl-3a,4,15b,15c-tetrahydro-5H,7H-[1,3]dioxolo[4',5':3,4]pyrido[1,2-a]benzo[e]pyrrolo[2,1-c][1,4]diazepin-4-ol (6b). white solid, yield 64%, petroleum ether/ ethyl acetate *V/V* = 1:1, m.p. 135.4-137.1 °C. $[\alpha]_D^{25}$ +90.0 (*c* 0.1, CH_3OH); ^{1}H NMR (400 MHz, Chloroform-*d*) δ 7.5 (ddd, *J* = 8.5, 5.4, 3.3 Hz, 1H), 7.4 (d, *J* = 7.7 Hz, 1H), 7.4 – 7.3 (m, 2H), 7.0 (t, *J* = 2.2 Hz, 1H), 6.4 (q, *J* = 3.5, 2.5 Hz, 2H), 4.7 (dd, *J* = 9.3, 5.0 Hz, 1H), 4.3 (dd, *J* = 5.1, 2.6 Hz, 1H), 4.1 (q, *J* = 2.5 Hz, 1H), 3.9 (d, *J* = 14.2 Hz, 1H), 3.4 (d, *J* = 14.2 Hz, 1H), 3.1 – 3.0 (m, 2H), 2.9 (dd, *J* = 11.8, 3.0 Hz, 1H), 1.4 (s, 3H), 1.3 (s, 3H). ^{13}C NMR (101 MHz, Chloroform-*d*) δ 130.7, 130.2, 129.3, 126.4, 121.9, 120.9, 109.3, 109.2, 106.7, 76.3, 73.7, 66.4, 59.7, 56.7, 56.2, 28.2, 26.1. MS (ESI): Calculated for $C_{19}H_{23}N_2O_3$ ($[M+H]^+$): 327.1703, found: 327.1705.

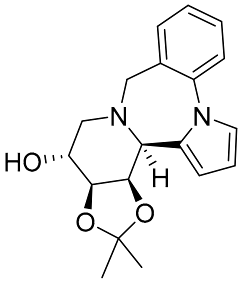

(3aS,4R,15bS,15cR)-2,2-dimethyl-3a,4,15b,15c-tetrahydro-5H,7H-[1,3]dioxolo[4',5':3,4]pyrido[1,2-a]benzo[e]pyrrolo[2,1-c][1,4]diazepin-4-ol (6b'). white solid, yield 6%, petroleum ether/ ethyl acetate *V/V* = 1:1, m.p. 186.6-188.3 ºC. $[\alpha]_D^{25}$ +42.0 (*c* 0.1, CH_3OH); ^1H NMR (400 MHz, Chloroform-*d*) δ 7.5 – 7.4 (m, 1H), 7.4 (d, *J* = 7.8 Hz, 1H), 7.3 (d, *J* = 3.9 Hz, 2H), 7.0 (t, *J* = 2.2 Hz, 1H), 6.7 (dd, *J* = 3.4, 1.6 Hz, 1H), 6.4 (t, *J* = 3.2 Hz, 1H), 4.5 (dd, *J* = 5.3, 2.0 Hz, 1H), 4.1 – 4.0 (m, 2H), 3.8 (dd, *J* = 7.3, 5.2 Hz, 1H), 3.5 (d, *J* = 14.4 Hz, 1H), 3.2 (s, 1H), 3.1 (dd, *J* = 10.9, 5.3 Hz, 1H), 2.4 (t, *J* = 10.8 Hz, 1H), 1.7 (s, 3H), 1.5 (s, 3H). ^{13}C NMR (101 MHz, Chloroform-*d*) δ 140.0, 130.6, 130.1, 129.2, 127.8, 126.3, 121.8, 120.1, 110.6, 109.3, 109.1, 77.2, 77.0, 75.4, 71.8, 57.1, 56.9, 28.6, 26.4. MS (ESI): Calculated for $C_{19}H_{23}N_2O_3$ ($[M+H]^+$): 327.1703, found: 327.1705.

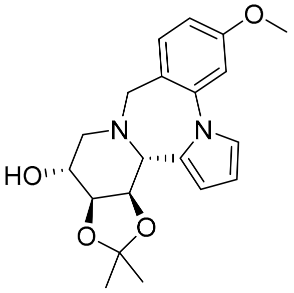

(3aS,4R,15bR,15cR)-10-methoxy-2,2-dimethyl-3a,4,15b,15c-tetrahydro-5H,7H-[1,3]dioxolo[4',5':3,4]pyrido[1,2-a]benzo[e]pyrrolo[2,1-c][1,4]diazepin-4-ol (7b). white solid, yield 70%, petroleum ether/ ethyl acetate *V/V* = 1:1, m.p. 165.2-167.6 ºC. $[\alpha]_D^{25}$ +87.0 (*c* 0.1, CH_3OH); ^1H NMR (400 MHz, Chloroform-*d*) δ 7.3 (d, *J* = 8.6 Hz, 1H), 7.0 – 6.9 (m, 2H), 6.8 (d, *J* = 2.8 Hz, 1H), 6.3 (d, *J* = 2.3 Hz, 2H), 4.7 (dd, *J* = 9.4, 5.0 Hz, 1H), 4.3 (dd, *J* = 5.2, 2.6 Hz, 1H), 4.1 – 4.0 (m, 1H), 3.8 (d, *J* = 12.3 Hz, 4H), 3.3 (d, *J* = 14.1 Hz, 1H), 3.0 – 2.9 (m, 2H), 2.8 (dd, *J* = 11.7, 3.0 Hz, 1H), 1.3 (s, 3H), 1.2 (s, 3H). ^{13}C NMR (101 MHz, Chloroform-*d*) δ 158.0, 130.6, 129.6, 123.0, 120.9, 115.6,

113.9, 109.3, 108.9, 106.3, 73.8, 66.4, 59.7, 56.9, 56.3, 55.7, 28.3, 26.2. MS (ESI): Calculated for $C_{20}H_{25}N_2O_4$ ($[M+H]^+$): 357.1809, found: 357.1811.

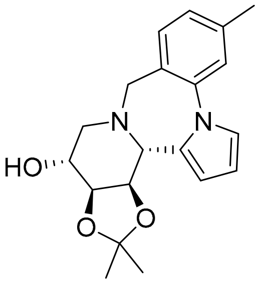

(3aS,4R,15bR,15cR)-2,2,10-trimethyl-3a,4,15b,15c-tetrahydro-5H,7H-[1,3]dioxolo[4',5':3,4]pyrido[1,2-a]benzo[e]pyrrolo[2,1-c][1,4]diazepin-4-ol (8b). Light yellow solid, yield 81%, petroleum ether/ ethyl acetate *V/V* = 1:1, m.p. 198.7-200.2 °C. $[\alpha]_D^{25}$ +84.0 (*c* 0.1, CH_3OH); ^1H NMR (400 MHz, Chloroform-*d*) δ 7.3 (d, *J* = 8.0 Hz, 1H), 7.2 (dd, *J* = 8.1, 1.9 Hz, 1H), 7.1 (d, *J* = 1.8 Hz, 1H), 7.0 (t, *J* = 2.2 Hz, 1H), 6.3 (d, *J* = 2.8 Hz, 2H), 4.7 (dd, *J* = 9.4, 5.0 Hz, 1H), 4.3 (dd, *J* = 5.1, 2.6 Hz, 1H), 4.1 – 4.0 (m, 1H), 3.9 (d, *J* = 14.2 Hz, 1H), 3.3 (d, *J* = 14.2 Hz, 1H), 3.1 (d, *J* = 10.4 Hz, 1H), 3.0 – 2.9 (m, 2H), 2.8 (dd, *J* = 11.8, 3.0 Hz, 1H), 2.4 (s, 3H), 1.3 (s, 3H), 1.2 (s, 3H). ^{13}C NMR (101 MHz, Chloroform-*d*) δ 137.5, 136.3, 130.8, 130.7, 129.8, 128.1, 121.7, 120.9, 109.3, 109.1, 106.5, 76.4, 73.8, 66.4, 59.8, 56.7, 56.3, 28.3, 26.2, 21.1. MS (ESI): Calculated for $C_{20}H_{25}N_2O_3$ ($[M+H]^+$): 341.1860, found: 341.1862.

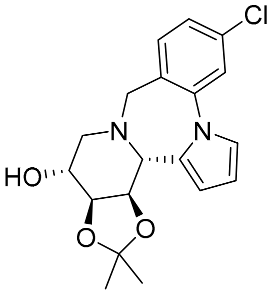

(3aS,4R,15bR,15cR)-10-chloro-2,2-dimethyl-3a,4,15b,15c-tetrahydro-5H,7H-[1,3]dioxolo[4',5':3,4]pyrido[1,2-a]benzo[e]pyrrolo[2,1-c][1,4]diazepin-4-ol (9b). Light yellow solid, yield 80%, petroleum ether/ ethyl acetate *V/V* = 1:1, m.p. 188.0-190.2 °C. $[\alpha]_D^{25}$ +92.0 (*c* 0.1, CH_3OH); ^1H NMR (400 MHz, Chloroform-*d*) δ 7.4 (dd, *J* = 8.4, 2.3 Hz, 1H), 7.4 (d, *J* = 8.4 Hz, 1H), 7.3 (d, *J* = 2.3 Hz, 1H), 7.0 (t, *J* = 2.2 Hz, 1H), 6.4 (d, *J* = 2.8 Hz, 2H), 4.7 (dd, *J* = 9.4, 5.0 Hz, 1H), 4.3 (dd, *J* = 5.1, 2.6 Hz, 1H), 4.1 – 4.0 (m, 1H), 3.9 (d, *J* = 14.3 Hz, 1H), 3.4 (d, *J* = 14.3 Hz, 1H), 3.1 (d, *J* = 10.3 Hz, 1H), 3.0 –

3.0 (m, 2H), 2.9 (dd, *J* = 11.7, 2.9 Hz, 1H), 1.4 (s, 3H), 1.3 (s, 3H). ^{13}C NMR (101 MHz, Chloroform-*d*) δ 131.8, 130.7, 130.1, 129.4, 123.2, 121.0, 109.7, 109.4, 107.2, 73.7, 66.3, 59.8, 56.4, 56.3, 28.3, 26.1. MS (ESI): Calculated for $C_{19}H_{22}ClN_2O_3$ ($[M+H]^+$): 361.1313, found: 361.1315.

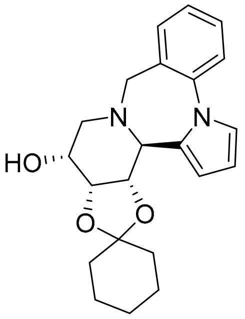

(3a'R,4'R,15b'S,15c'S)-3a',4',15b',15c'-tetrahydro-5'H,7'H-spiro[cyclohexane-1,2'-[1,3]dioxolo[4',5':3,4]pyrido[1,2-a]benzo[e]pyrrolo[2,1-c][1,4]diazepin]-4'-ol (10b). Light yellow solid, yield 81%, petroleum ether/ ethyl acetate *V/V* = 1:1, m.p. 162.5-163.2 °C. $[\alpha]_D^{25}$ -96.0 (*c* 0.1, CH_3OH); ^{1}H NMR (400 MHz, Chloroform-*d*) δ 7.5 – 7.4 (m, 2H), 7.4 – 7.3 (m, 2H), 7.0 (dd, *J* = 2.7, 1.8 Hz, 1H), 6.4 (t, *J* = 2.1 Hz, 2H), 4.6 (dd, *J* = 9.2, 5.1 Hz, 1H), 4.5 (t, *J* = 4.8 Hz, 1H), 4.2 (dt, *J* = 9.1, 4.3 Hz, 1H), 3.9 (d, *J* = 14.2 Hz, 1H), 3.4 (d, *J* = 14.2 Hz, 1H), 3.0 (d, *J* = 9.3 Hz, 1H), 3.0 – 3.0 (m, 1H), 2.7 (dd, *J* = 10.8, 9.3 Hz, 1H), 2.3 (d, *J* = 9.7 Hz, 1H), 1.8 – 1.4 (m, 11H). ^{13}C NMR (101 MHz, Chloroform-*d*) δ 140.1, 130.8, 130.4, 129.2, 128.4, 126.3, 121.9, 120.9, 109.9, 109.2, 106.9, 74.8, 74.3, 67.1, 58.7, 56.5, 55.8, 37.7, 35.0, 25.0, 24.1, 23.8. MS (ESI): Calculated for $C_{22}H_{27}N_2O_3$ ($[M+H]^+$): 367.2016, found: 367.2019.

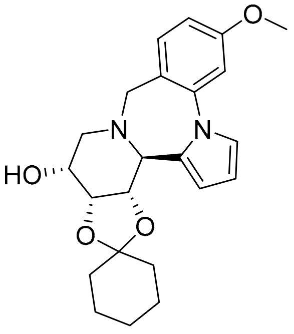

(3a'R,4'R,15b'S,15c'S)-10'-methoxy-3a',4',15b',15c'-tetrahydro-5'H,7'H-spiro[cyclohexane-1,2'-[1,3]dioxolo[4',5':3,4]pyrido[1,2-a]benzo[e]pyrrolo[2,1-c][1,4]diazepin]-4'-ol (11b). Light yellow solid, yield 74%, petroleum ether/ ethyl

acetate *V/V* = 1:1, m.p. 123.0-124.4 ºC. $[\alpha]_D^{25}$ -84.0 (*c* 0.1, CH_3OH); ^1H NMR (400 MHz, Chloroform-*d*) δ 7.3 (dd, *J* = 8.6, 2.2 Hz, 1H), 6.9 (ddd, *J* = 7.3, 5.4, 2.4 Hz, 2H), 6.8 (t, *J* = 2.5 Hz, 1H), 6.3 (d, *J* = 2.5 Hz, 2H), 4.6 (ddd, *J* = 9.4, 5.1, 2.2 Hz, 1H), 4.4 (td, *J* = 4.7, 2.0 Hz, 1H), 4.1 – 4.0 (m, 1H), 3.8 (d, *J* = 2.2 Hz, 3H), 3.8 (dd, *J* = 14.1, 2.2 Hz, 1H), 3.3 (dd, *J* = 14.1, 2.2 Hz, 1H), 3.0 – 2.9 (m, 2H), 2.7 – 2.6 (m, 1H), 2.5 (s, 1H), 1.7 – 1.3 (m, 10H). ^{13}C NMR (101 MHz, Chloroform-*d*) δ 157.8, 133.2, 130.7, 129.6, 122.9, 120.8, 115.6, 113.9, 109.9, 108.8, 106.4, 74.8, 74.4, 67.0, 58.7, 56.6, 55.8, 55.6, 37.8, 35.0, 25.0, 24.1, 23.8. MS (ESI): Calculated for $C_{23}H_{29}N_2O_4$ ($[M+H]^+$): 397.2122, found: 397.2124.

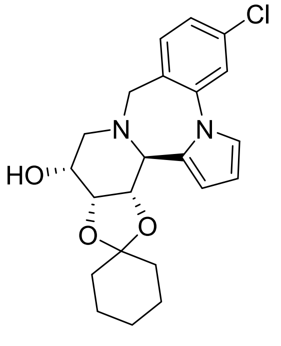

(3a'R,4'R,15b'S,15c'S)-10'-chloro-3a',4',15b',15c'-tetrahydro-5'H,7'H-spiro[cyclohexane-1,2'-[1,3]dioxolo[4',5':3,4]pyrido[1,2-a]benzo[e]pyrrolo[2,1-c][1,4]diazepin]-4'-ol (12b). Light yellow solid, yield 80%, petroleum ether/ ethyl acetate *V/V* = 1:1, m.p. 154.8-156.2 ºC. $[\alpha]_D^{25}$ -92.0 (*c* 0.1, CH_3OH); ^1H NMR (400 MHz, Chloroform-*d*) δ 7.4 (dd, *J* = 8.4, 2.3 Hz, 1H), 7.4 (d, *J* = 8.4 Hz, 1H), 7.3 (d, *J* = 2.4 Hz, 1H), 7.0 (dd, *J* = 2.7, 1.7 Hz, 1H), 6.4 (t, *J* = 2.5 Hz, 2H), 4.6 (dd, *J* = 9.3, 5.1 Hz, 1H), 4.5 (t, *J* = 4.8 Hz, 1H), 4.2 – 4.1 (m, 1H), 3.8 (d, *J* = 14.3 Hz, 1H), 3.4 (d, *J* = 14.2 Hz, 1H), 3.1 – 3.0 (m, 2H), 2.7 (dd, *J* = 10.7, 9.4 Hz, 1H), 1.8 – 1.4 (m, 10H). ^{13}C NMR (101 MHz, Chloroform-*d*) δ 138.6, 131.7, 130.8, 130.2, 130.1, 129.2, 123.1, 120.9, 110.0, 109.6, 107.3, 74.7, 74.3, 67.0, 58.7, 56.1, 55.7, 37.8, 35.0, 25.0, 24.1, 23.8. MS (ESI): Calculated for $C_{22}H_{26}ClN_2O_3$ ($[M+H]^+$): 401.1626, found: 401.1628.

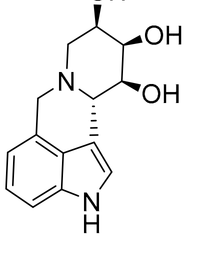

(9*R*,10*R*,11*S*)-2,8,9,10,11,11a-hexahydro-6*H*-pyrido[1,2-*b*]pyrrolo[4,3,2-*de*]isoquinoline-9,10,11-triol (1c). Yellow solid, yield 75%, DCM:MeOH *V*/*V* = 30:1, m.p. 125.4 - 126.6 ºC, $[\alpha]_D^{25}$ -74.0 (c 0.1, CH_3OH); ^1H NMR (600 MHz, CD_3OD) δ 7.35 (s, 1H), 7.24 (d, *J* = 8.2 Hz, 1H), 7.10 (t, *J* = 7.6 Hz, 1H), 6.84 (d, *J* = 7.0 Hz, 1H), 4.32 – 4.15 (m, 3H), 4.10 (s, 1H), 3.89 (td, *J* = 7.8, 2.7 Hz, 1H), 3.79 – 3.68 (m, 1H), 3.12 (d, *J* = 8.7 Hz, 2H). ^{13}C NMR (151 MHz, CD_3OD) δ 133.4, 126.0, 124.8, 122.1, 120.9, 119.7, 113.2, 109.6, 72.2, 71.4, 66.0, 57.9, 55.8, 52.8. MS (ESI): Calculated for $C_{14}H_{17}N_2O_3$ ($[M+H]^+$): 216.1234, found: 216.1235.

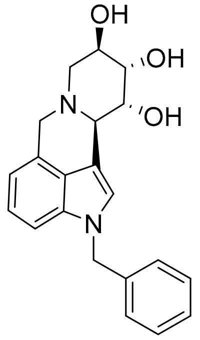

(9*R*,10*S*,11*R*)-2-benzyl-2,8,9,10,11,11a-hexahydro-6*H*-pyrido[1,2-*b*]pyrrolo[4,3,2-*de*]isoquinoline-9,10,11-triol (2c). Yellow solid, yield 70%, DCM:MeOH *V*/*V* = 30:1, m.p. 132.3 - 133.0 ºC, $[\alpha]_D^{25}$ +78.0 (c 0.1, CH_3OH); ^1H NMR (400 MHz, CD_3OD) δ 7.31 – 7.20 (m, 4H), 7.18 – 7.14 (m, 2H), 7.12 – 7.06 (m, 2H), 6.80 (d, *J* = 6.5 Hz, 1H), 5.32 (s, 2H), 4.26 – 3.68 (m, 6H), 2.93 (s, 2H).^{13}C NMR (101 MHz, CD_3OD) δ 138.4, 134.0, 128.3, 127.1, 126.7, 122.5, 107.6, 71.6, 70.8, 67.6, 55.3, 49.7, 47.8, 32.3. MS (ESI): Calculated for $C_{21}H_{23}N_2O_3$ ($[M+H]^+$): 351.1703, found: 351.1700. MS (ESI): Calculated for $C_{21}H_{23}N_2O_3$ ($[M+H]^+$): 351.1703, found: 351.1706.

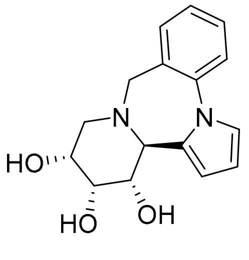

(12R,13R,14S,14aS)-12,13,14,14a-tetrahydro-9H,11H-benzo[e]pyrido[1,2-a]pyrrolo[2,1-c][1,4]diazepine-12,13,14-triol (3c). White solid, yield 83%, DCM:MeOH *V*/*V* = 7:1, m.p. 106.3-108.6 ºC, $[\alpha]_D^{25}$ -72.0 (c 0.1, CH_3OH); ^1H NMR (600

MHz, Chloroform-*d*) δ 7.4 (d, *J* = 7.5 Hz, 1H), 7.4 (d, *J* = 7.9 Hz, 1H), 7.3 (q, *J* = 7.2 Hz, 2H), 7.0 (s, 1H), 6.3 (d, *J* = 3.8 Hz, 1H), 6.3 (d, *J* = 3.4 Hz, 1H), 4.1 (t, *J* = 6.9 Hz, 2H), 3.9 (t, *J* = 6.0 Hz, 1H), 3.7 – 3.6 (m, 1H), 3.4 (d, *J* = 13.7 Hz, 1H), 3.3 – 3.2 (m, 1H), 3.0 (s, 3H), 2.8 (p, *J* = 10.3 Hz, 2H). ^{13}C NMR (151 MHz, Chloroform-*d*) δ 139.8, 130.6, 129.6, 129.3, 128.8, 126.6, 121.8, 120.9, 109.3, 106.2, 77.2, 69.8, 68.4, 56.7, 56.4, 54.1. MS (ESI): Calculated for $C_{16}H_{19}N_2O_3$ ($[M+H]^+$): 287.1390, found: 287.1391.

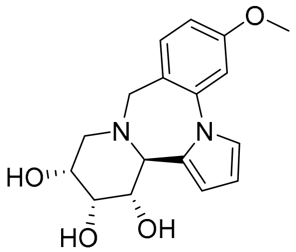

(12R,13R,14S,14aS)-6-methoxy-12,13,14,14a-tetrahydro-9H,11H-benzo[e]pyrido[1,2-a]pyrrolo[2,1-c][1,4]diazepine-12,13,14-triol (4c). White solid, yield 87%, DCM:MeOH *V*/*V* = 7:1, m.p. 110.1-112.2 ºC, $[\alpha]_D^{25}$ -90.0 (c 0.1, CH_3OH); ^{1}H NMR (400 MHz, Chloroform-*d*) δ 7.3 (d, *J* = 8.6 Hz, 1H), 7.0 – 6.9 (m, 2H), 6.8 (d, *J* = 2.8 Hz, 1H), 6.3 (t, *J* = 3.2 Hz, 1H), 6.2 (dd, *J* = 3.6, 1.4 Hz, 1H), 4.2 (t, *J* = 3.2 Hz, 1H), 4.1 (dd, *J* = 8.8, 3.1 Hz, 1H), 3.9 (td, *J* = 7.3, 3.2 Hz, 1H), 3.8 (s, 3H), 3.7 (d, *J* = 13.6 Hz, 1H), 3.4 (d, *J* = 13.8 Hz, 1H), 3.3 (d, *J* = 8.9 Hz, 1H), 2.8 (d, *J* = 7.2 Hz, 2H). ^{13}C NMR (101 MHz, Chloroform-*d*) δ 158.2, 132.9, 130.2, 129.5, 122.9, 121.1, 115.9, 114.1, 109.0, 105.4, 69.8, 69.7, 68.5, 56.9, 56.3, 55.7, 54.3. MS (ESI): Calculated for $C_{17}H_{21}N_2O_4$ ($[M+H]^+$): 317.1496, found: 317.1499.

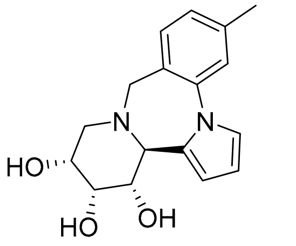

(12R,13R,14S,14aS)-6-methyl-12,13,14,14a-tetrahydro-9H,11H-benzo[e]pyrido[1,2-a]pyrrolo[2,1-c][1,4]diazepine-12,13,14-triol (5c). White solid, yield 82%, DCM:MeOH *V*/*V* = 7:1, m.p. 84.5-85.2 ºC, $[\alpha]_D^{25}$ -84.0 (c 0.1, CH_3OH); ^{1}H NMR (400 MHz, Methanol-d_4) δ 7.3 (d, *J* = 2.7 Hz, 2H), 7.2 (s, 1H), 7.0 (t, *J* = 2.1 Hz, 1H), 6.3 (t, *J* = 2.3 Hz, 1H), 6.3 (t, *J* = 3.2 Hz, 1H), 4.1 – 4.0 (m, 2H), 3.8 (ddd, *J* = 10.4, 5.1, 2.8 Hz,

1H), 3.7 (d, J = 13.4 Hz, 1H), 3.5 (d, J = 13.9 Hz, 1H), 3.3 (d, J = 9.9 Hz, 1H), 2.9 (t, J = 10.4 Hz, 1H), 2.8 (dd, J = 10.4, 5.1 Hz, 1H), 2.4 (s, 3H). ^{13}C NMR (101 MHz, Methanol-d_4) δ 137.7, 136.3, 130.9, 130.2, 129.6, 128.0, 121.3, 119.6, 108.5, 106.7, 71.1, 68.8, 67.9, 55.9, 55.3, 53.0, 19.7. MS (ESI): Calculated for $C_{17}H_{21}N_2O_3$ ([M+H]$^+$): 301.1547, found: 301.1549.

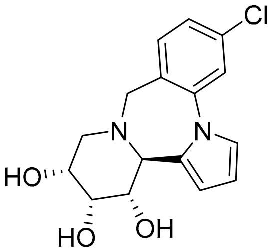

(12R,13R,14S,14aS)-6-chloro-12,13,14,14a-tetrahydro-9H,11H-benzo[e]pyrido[1,2-a]pyrrolo[2,1-c][1,4]diazepine-12,13,14-triol (6c). White solid, yield 84%, DCM:MeOH V/V = 7:1, m.p. 96.1-97.8 ºC, $[\alpha]_D^{25}$ -95.0 (c 0.1, CH_3OH); ^{1}H NMR (400 MHz, Chloroform-d) δ 7.5 (dd, J = 8.4, 2.3 Hz, 1H), 7.4 – 7.3 (m, 2H), 7.0 (dd, J = 2.9, 1.5 Hz, 1H), 6.4 (t, J = 3.2 Hz, 1H), 6.4 – 6.3 (m, 1H), 4.3 (t, J = 3.2 Hz, 1H), 4.2 (dd, J = 9.0, 3.2 Hz, 1H), 4.0 (s, 1H), 3.7 (d, J = 13.9 Hz, 1H), 3.5 – 3.4 (m, 1H), 3.3 (d, J = 9.1 Hz, 1H), 2.9 – 2.8 (m, 2H). ^{13}C NMR (101 MHz, Chloroform-d) δ 138.3, 132.2, 130.5, 129.3, 123.2, 121.2, 113.3, 109.8, 106.2, 69.8, 69.7, 68.4, 56.5, 56.2, 54.2. MS (ESI): Calculated for $C_{16}H_{18}ClN_2O_3$ ([M+H]$^+$): 321.1000, found: 321.1002.

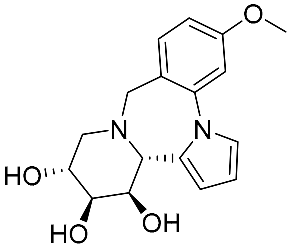

(12R,13S,14R,14aR)-6-methoxy-12,13,14,14a-tetrahydro-9H,11H-benzo[e]pyrido[1,2-a]pyrrolo[2,1-c][1,4]diazepine-12,13,14-triol (7c). Light yellow solid, yield 85%, DCM:MeOH V/V = 7:1, m.p. 131.0-132.4 ºC, $[\alpha]_D^{25}$ +53.0 (c 0.1, CH_3OH); ^{1}H NMR (400 MHz, Methanol-d_4) δ 7.4 (d, J = 8.5 Hz, 1H), 7.0 (dd, J = 8.6, 2.9 Hz, 1H), 7.0 – 7.0 (m, 2H), 6.3 (dd, J = 4.0, 1.5 Hz, 1H), 6.3 (t, J = 3.2 Hz, 1H), 4.3 (dd, J = 7.7, 2.8 Hz, 1H), 3.9 (dd, J = 3.8, 2.5 Hz, 2H), 3.9 (s, 3H), 3.5 (d, J = 8.6 Hz, 3H), 3.0 (dd, J = 11.6, 2.2 Hz, 1H), 2.6 (s, 1H). ^{13}C NMR (101 MHz, Methanol-d_4) δ 158.3,

133.3, 130.1, 122.5, 119.4, 115.6, 113.9, 108.2, 106.8, 71.7, 68.7, 68.1, 57.1, 56.2, 54.7, 54.2. MS (ESI): Calculated for $C_{17}H_{21}N_2O_4$ ($[M+H]^+$): 317.1496, found: 317.1499.

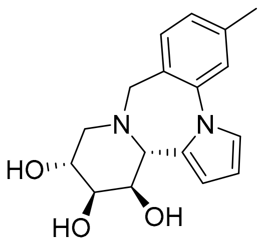

(12R,13S,14R,14aR)-6-methyl-12,13,14,14a-tetrahydro-9H,11H-benzo[e]pyrido[1,2-a]pyrrolo[2,1-c][1,4]diazepine-12,13,14-triol (8c). Light yellow solid, yield 80%, DCM:MeOH *V*/*V* = 7:1, m.p. 110.6-112.4 ºC, $[\alpha]_D^{25}$ +68.0 (c 0.1, CH_3OH); ^{1}H NMR (400 MHz, Methanol-*d*4) δ 7.3 (d, *J* = 2.0 Hz, 2H), 7.2 (s, 1H), 7.0 (dd, *J* = 2.8, 1.5 Hz, 1H), 6.3 (dd, *J* = 3.8, 1.4 Hz, 1H), 6.3 (t, *J* = 3.2 Hz, 1H), 4.3 (dd, *J* = 7.6, 2.5 Hz, 1H), 3.9 – 3.8 (m, 2H), 3.6 – 3.4 (m, 3H), 3.0 – 3.0 (m, 1H), 2.6 (d, *J* = 12.2 Hz, 1H), 2.4 (s, 3H). ^{13}C NMR (101 MHz, Methanol-*d*4) δ 137.8, 136.2, 130.9, 129.5, 128.6, 121.3, 119.4, 108.4, 107.1, 71.8, 68.6, 68.1, 57.1, 56.1, 54.2, 19.7. MS (ESI): Calculated for $C_{17}H_{21}N_2O_3$ ($[M+H]^+$): 301.1547, found: 301.1549.

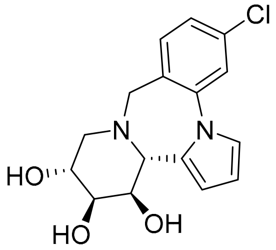

(12R,13S,14R,14aR)-6-chloro-12,13,14,14a-tetrahydro-9H,11H-benzo[e]pyrido[1,2-a]pyrrolo[2,1-c][1,4]diazepine-12,13,14-triol (9c). Yellow solid, yield 85%, DCM:MeOH *V*/*V* = 7:1, m.p. 90.5-92.1 ºC, $[\alpha]_D^{25}$ +70.0 (c 0.1, CH_3OH); ^{1}H NMR (400 MHz, Methanol-*d*4) δ 7.5 – 7.5 (m, 2H), 7.4 (d, *J* = 8.3 Hz, 1H), 7.0 (dd, *J* = 2.8, 1.5 Hz, 1H), 6.4 (dd, *J* = 3.8, 1.5 Hz, 1H), 6.3 (t, *J* = 3.2 Hz, 1H), 4.3 (dd, *J* = 8.1, 3.0 Hz, 1H), 3.9 – 3.8 (m, 2H), 3.5 – 3.4 (m, 3H), 3.0 (dd, *J* = 11.8, 2.3 Hz, 1H), 2.7 – 2.6 (m, 1H). ^{13}C NMR (101 MHz, Methanol-*d*4) δ 139.0, 131.4, 130.8, 130.1, 129.0, 123.0, 119.6, 109.0, 107.6, 71.6, 68.7, 67.8, 57.0, 55.7, 54.0. MS (ESI): Calculated for $C_{16}H_{18}ClN_2O_3$ ($[M+H]^+$): 321.1000, found: 321.1002.

NMR spectra of the compunds:

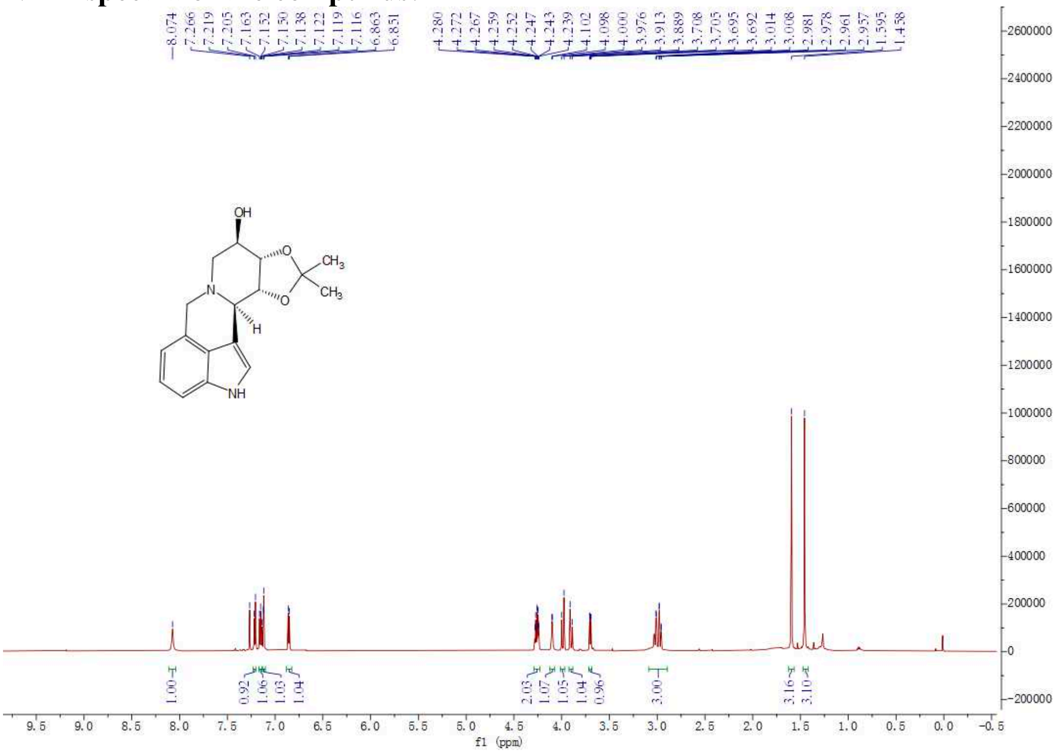

Figure. S1 ^{1}H NMR of compound **2a**

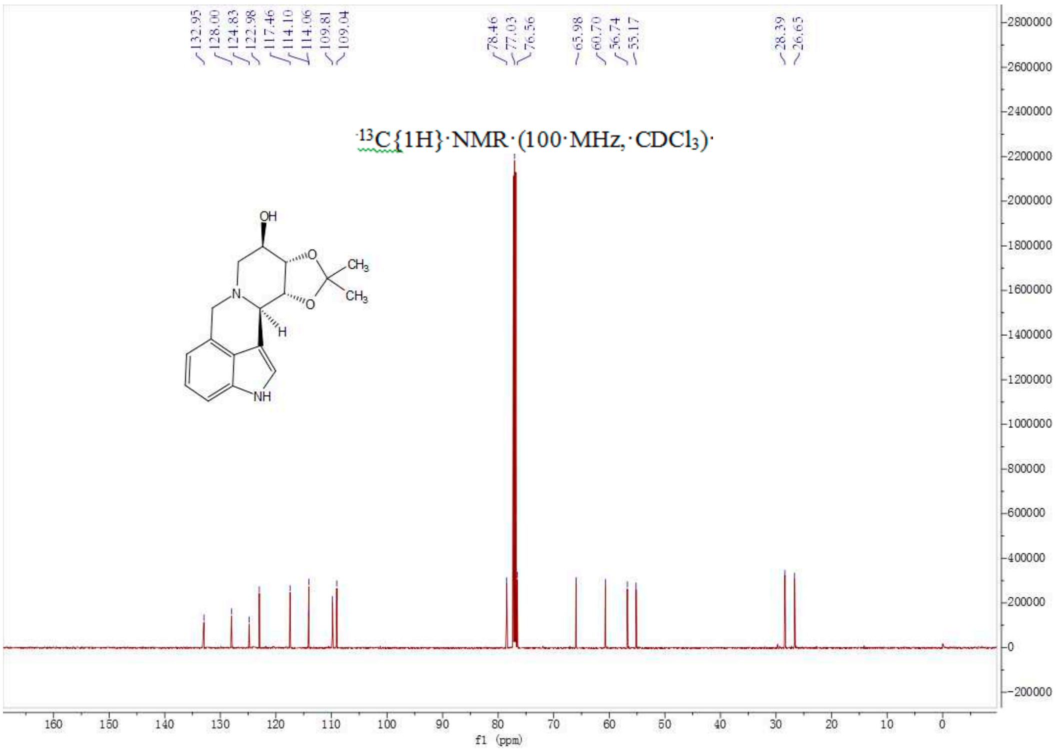

Figure. S2 ^{13}C{^{1}H} NMR of compound **2a**

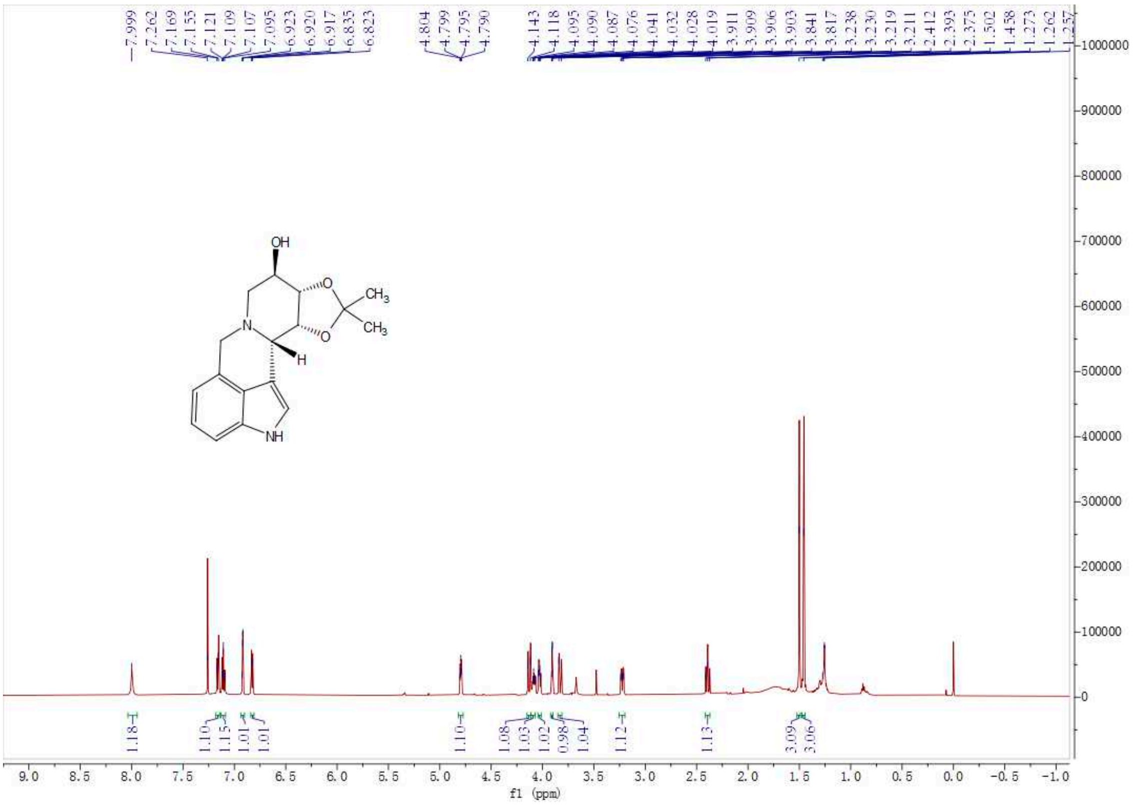

Figure. S3 ^{1}H NMR of compound **2a’**

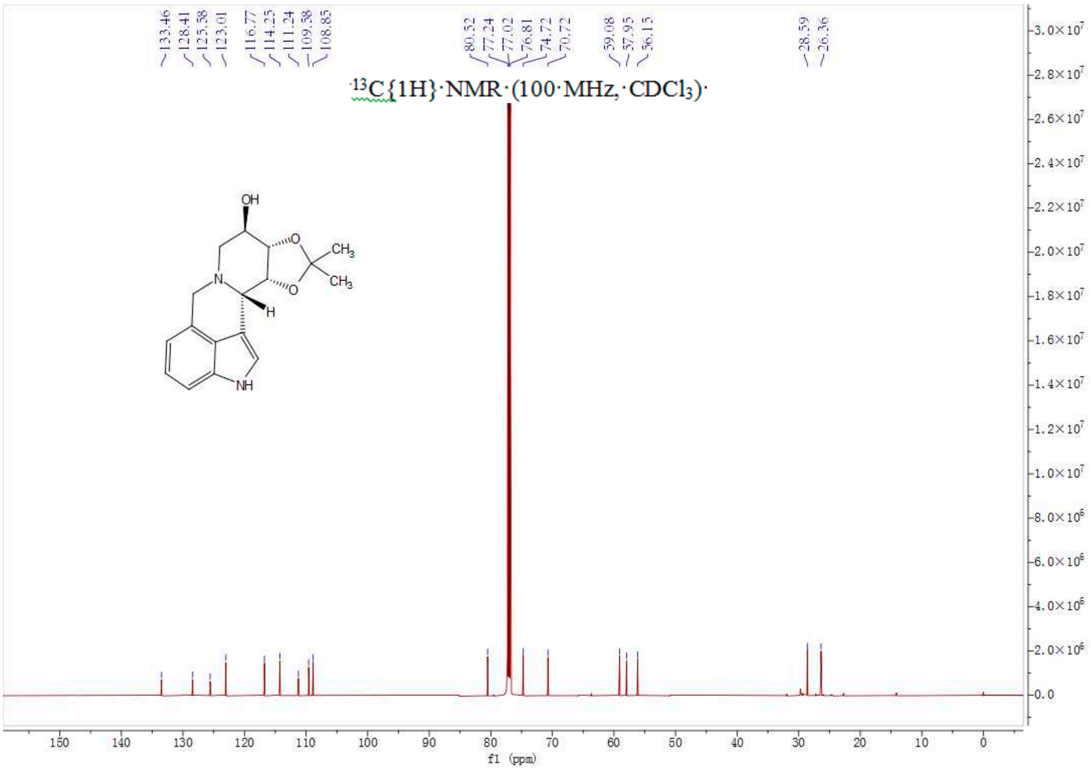

Figure. S4 ^{13}C{^{1}H} NMR of compound **2a’**

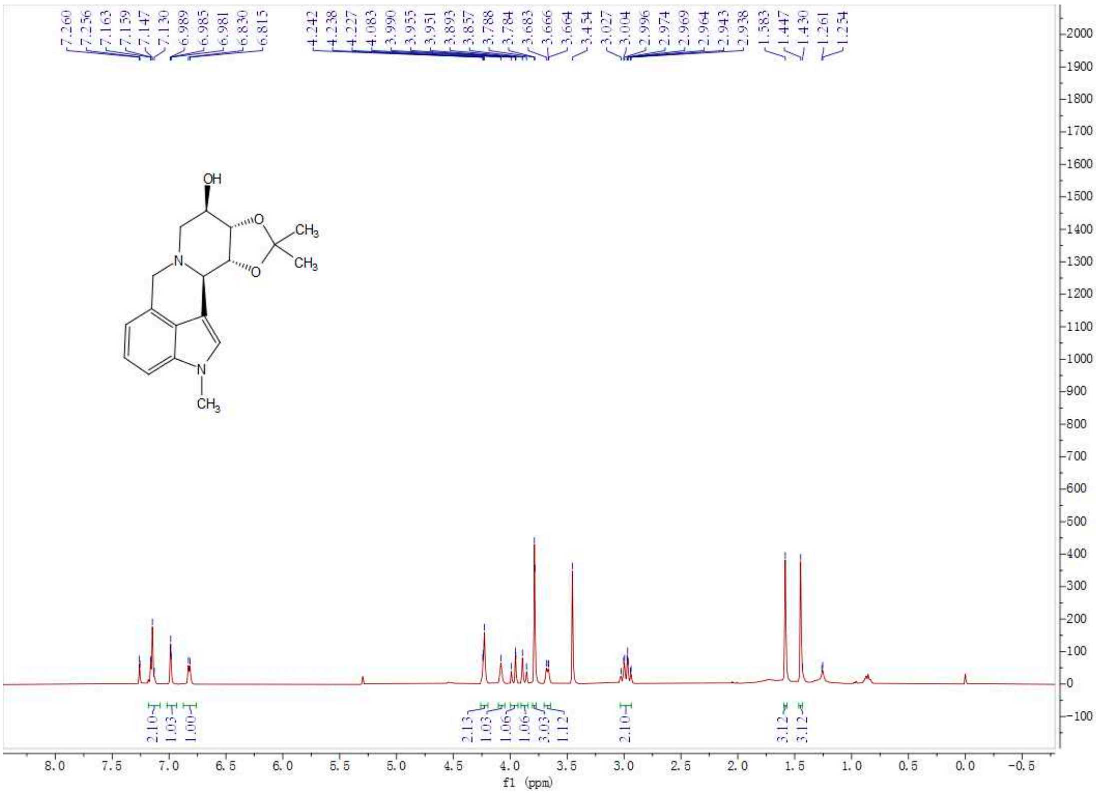

Figure. S5 ^{1}H NMR of compound **3a**

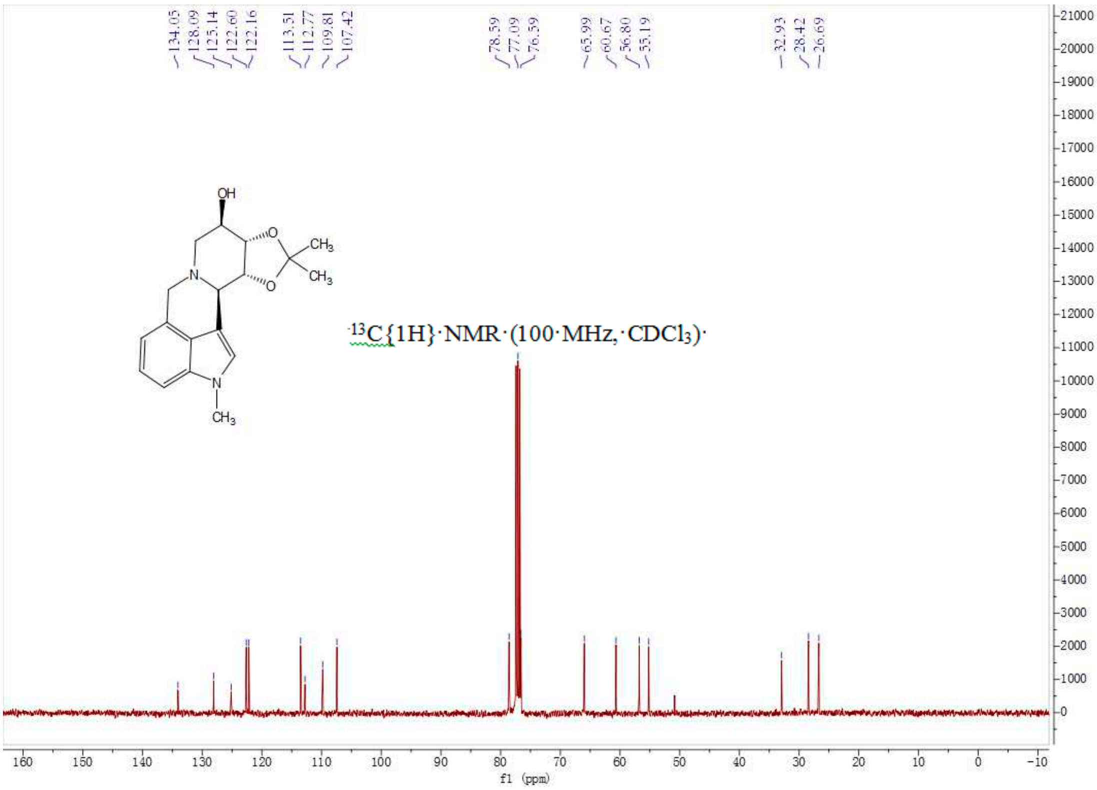

Figure. S6 ^{13}C{^{1}H} NMR of compound **3a**

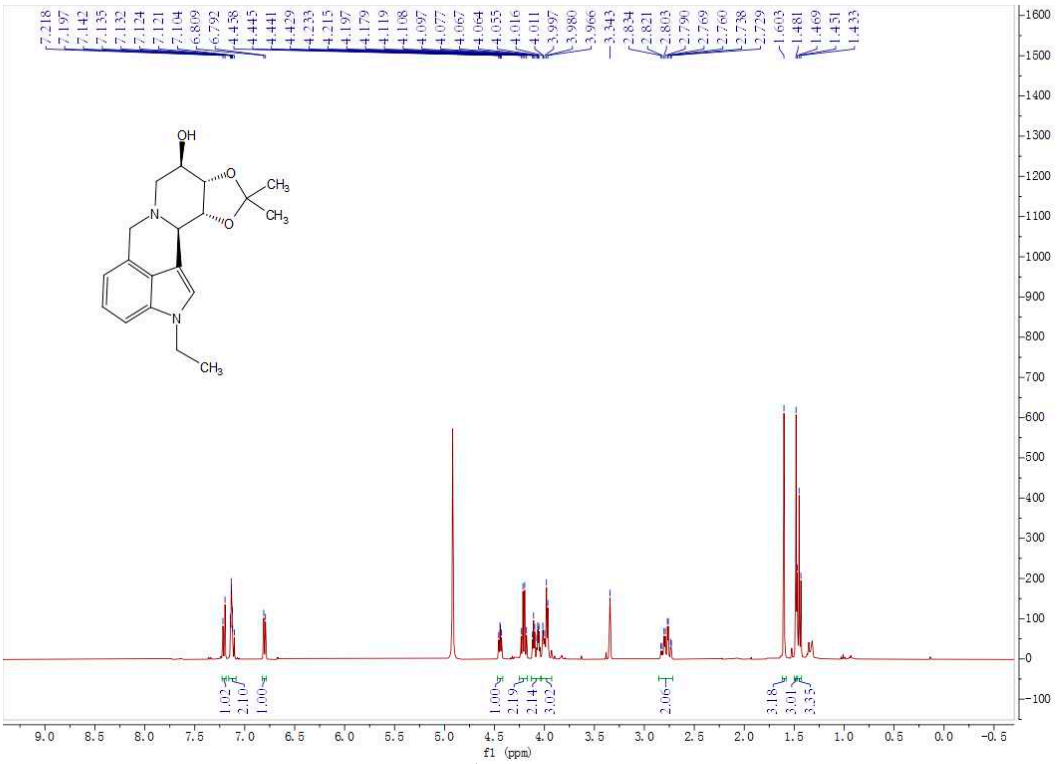

Figure. S7 ^{1}H NMR of compound **4a**

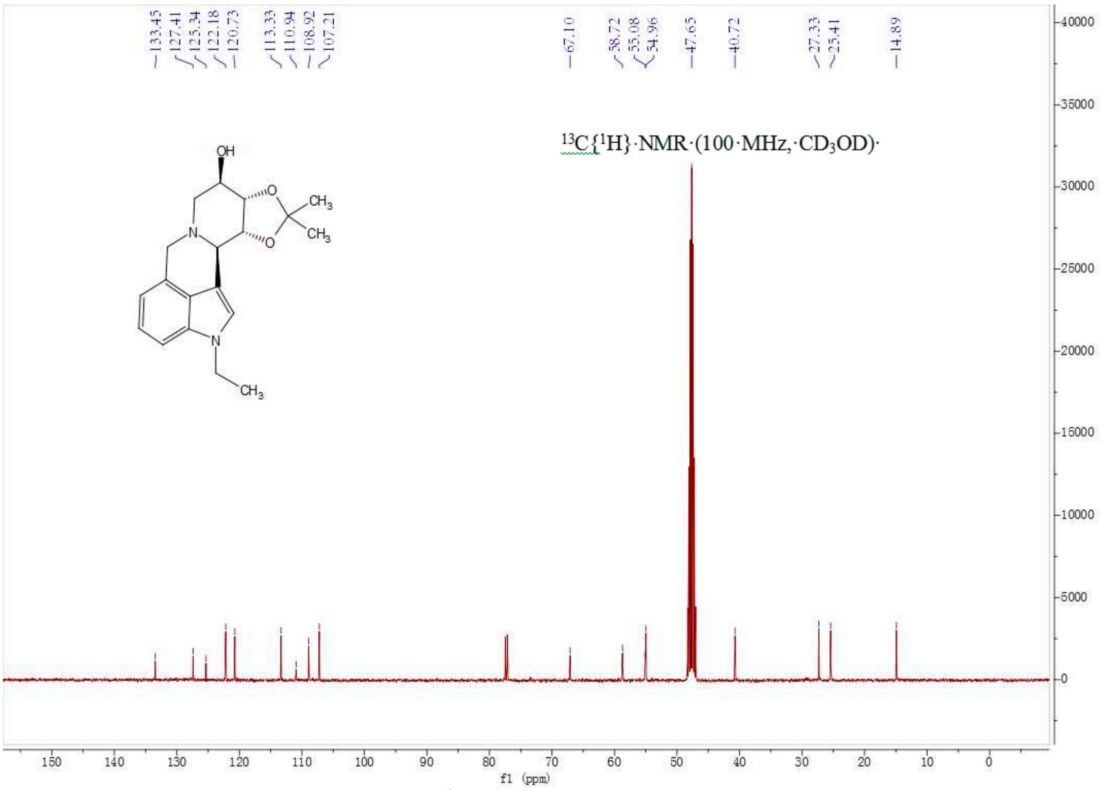

Figure. S8 $^{13}C\{^{1}H\}$ NMR of compound **4a**

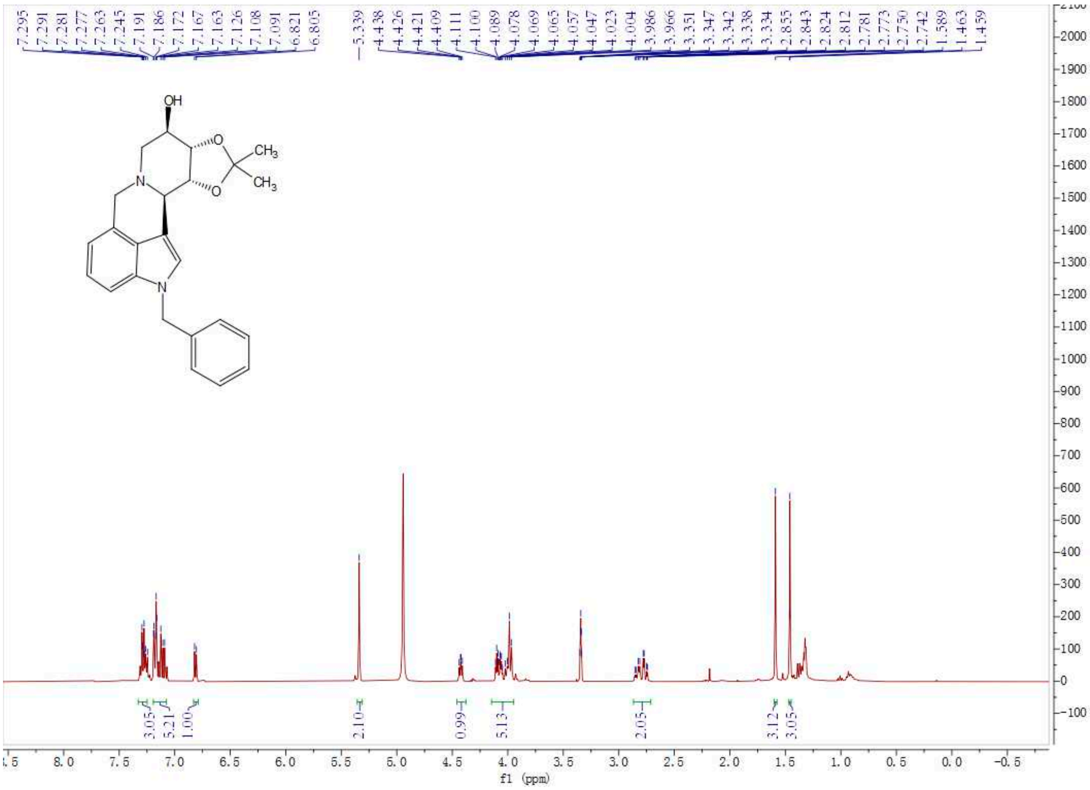

Figure. S9 ^{1}H NMR of compound **5a**

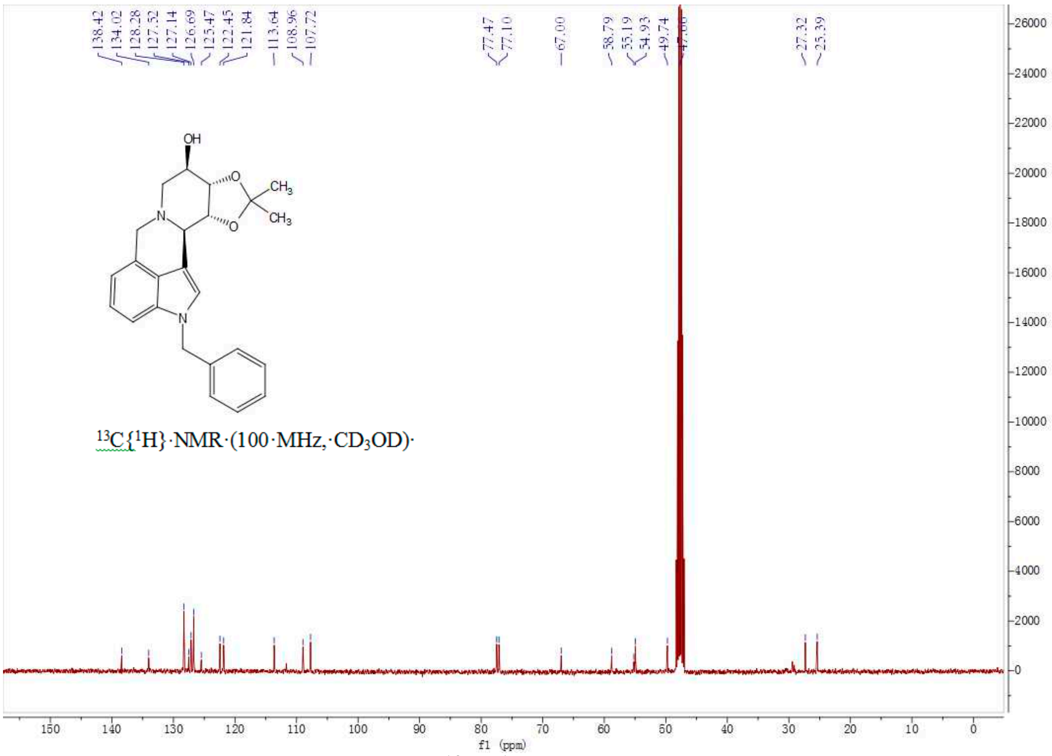

Figure. S10 ^{13}C{^{1}H} NMR of compound **5a**

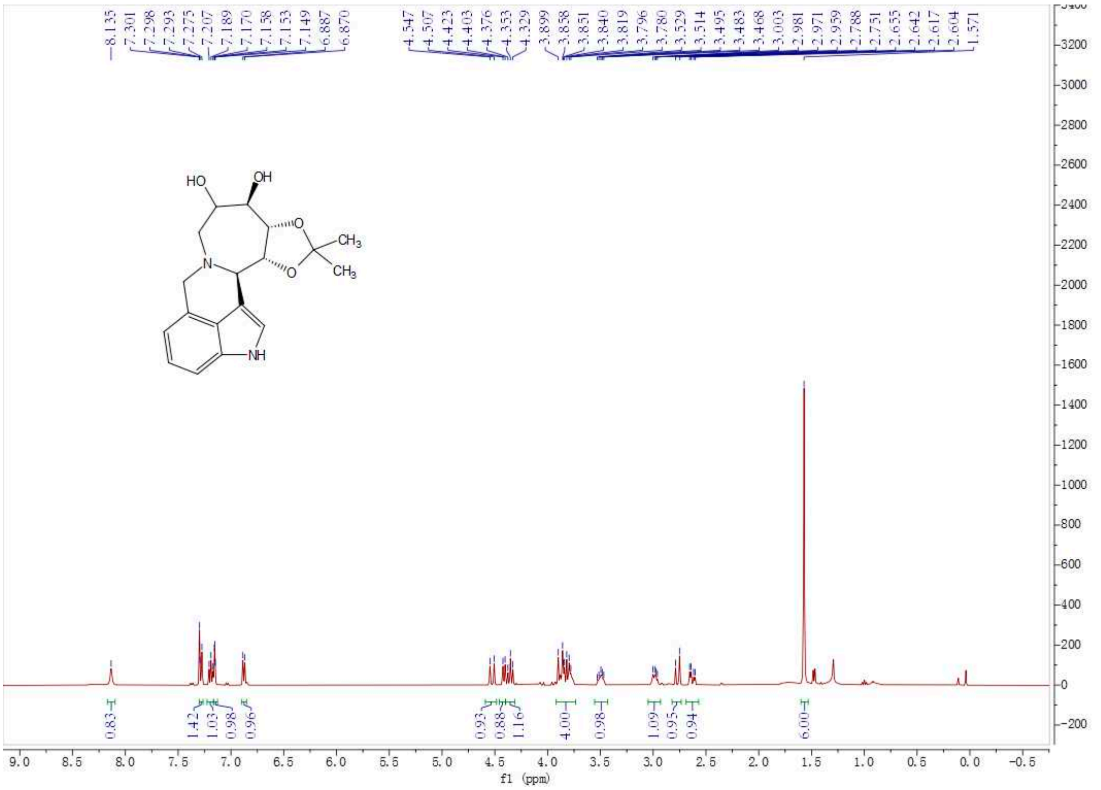

Figure. S11 ^{1}H NMR of compound **6a**

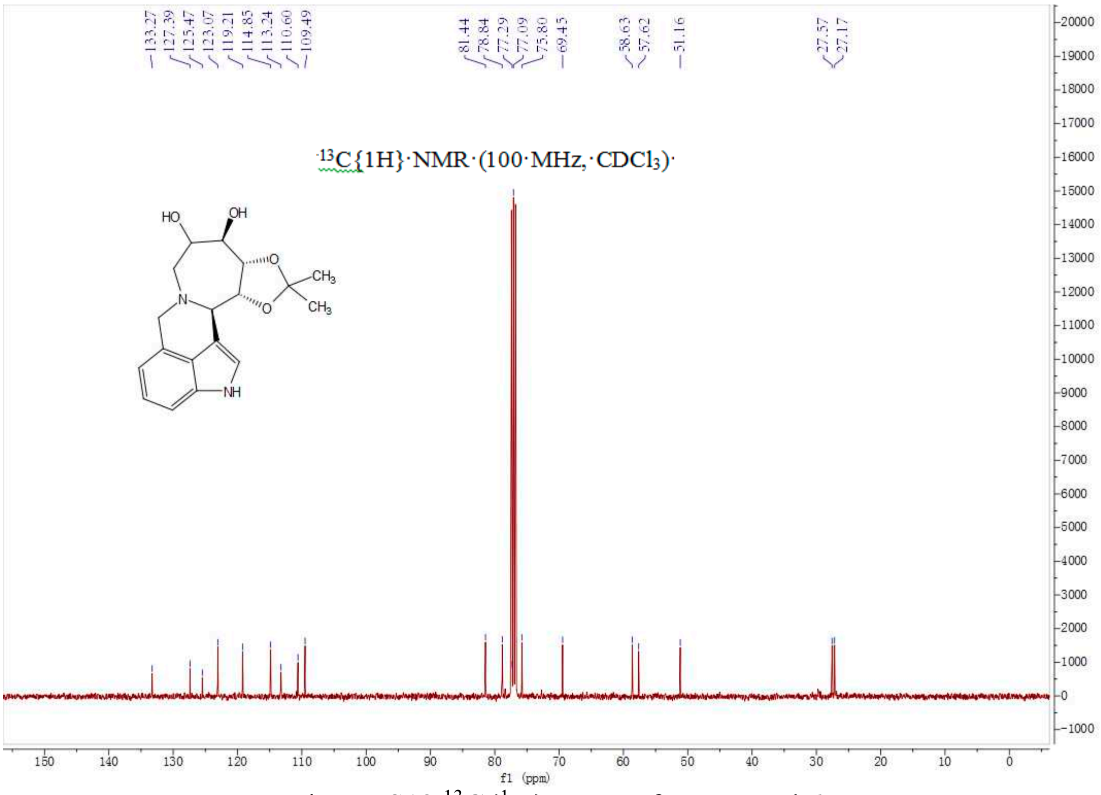

Figure. S12 ^{13}C{^{1}H} NMR of compound **6a**

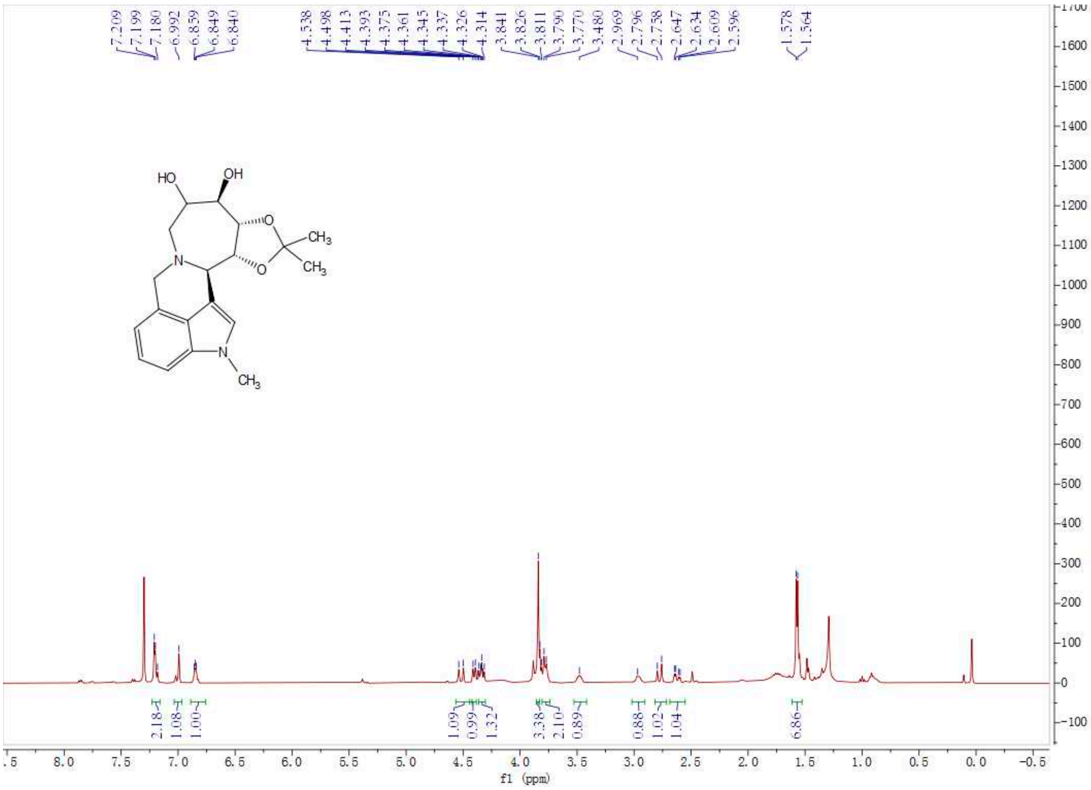

Figure. S13 ^{1}H NMR of compound **7a**

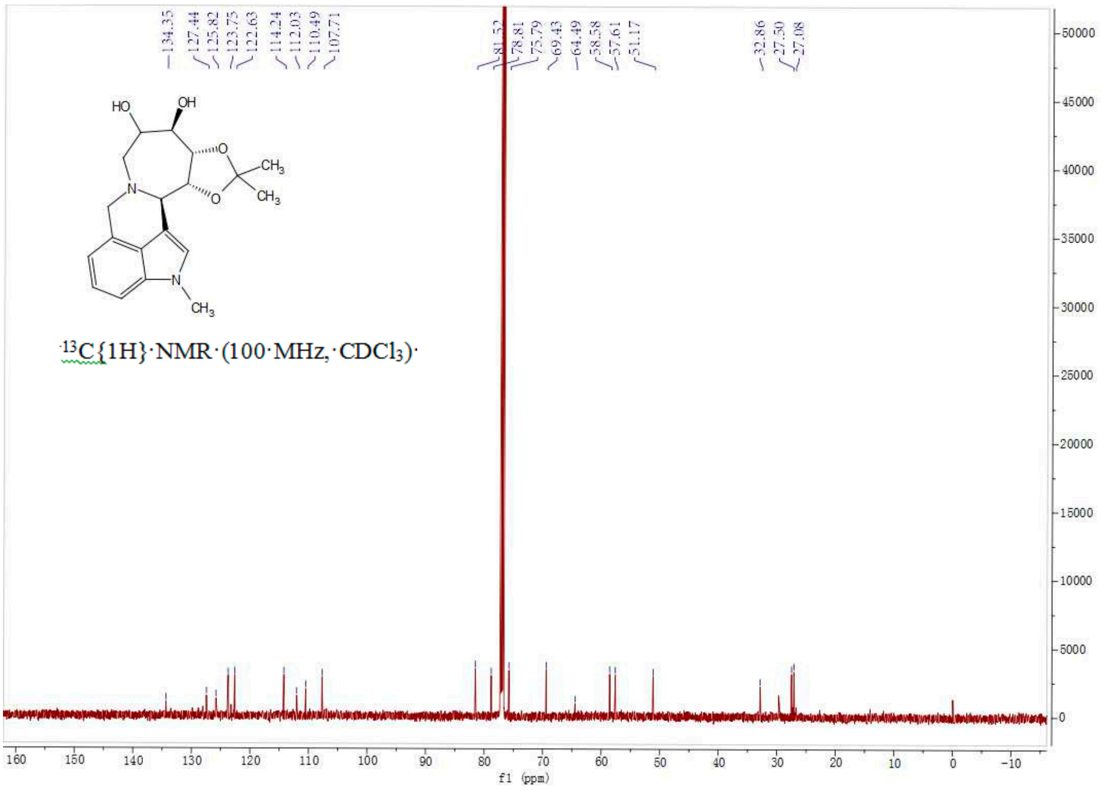

Figure. S14 ^{13}C{^{1}H} NMR of compound **7a**

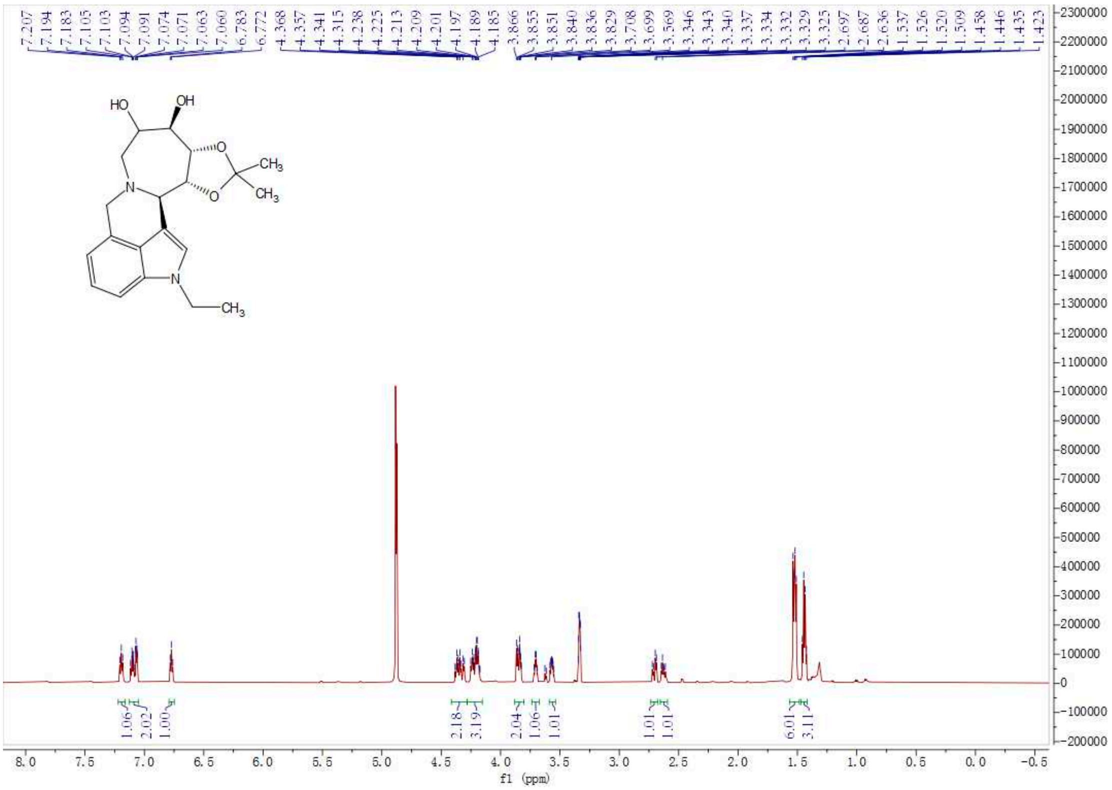

Figure. S15 ^{1}H NMR of compound **8a**

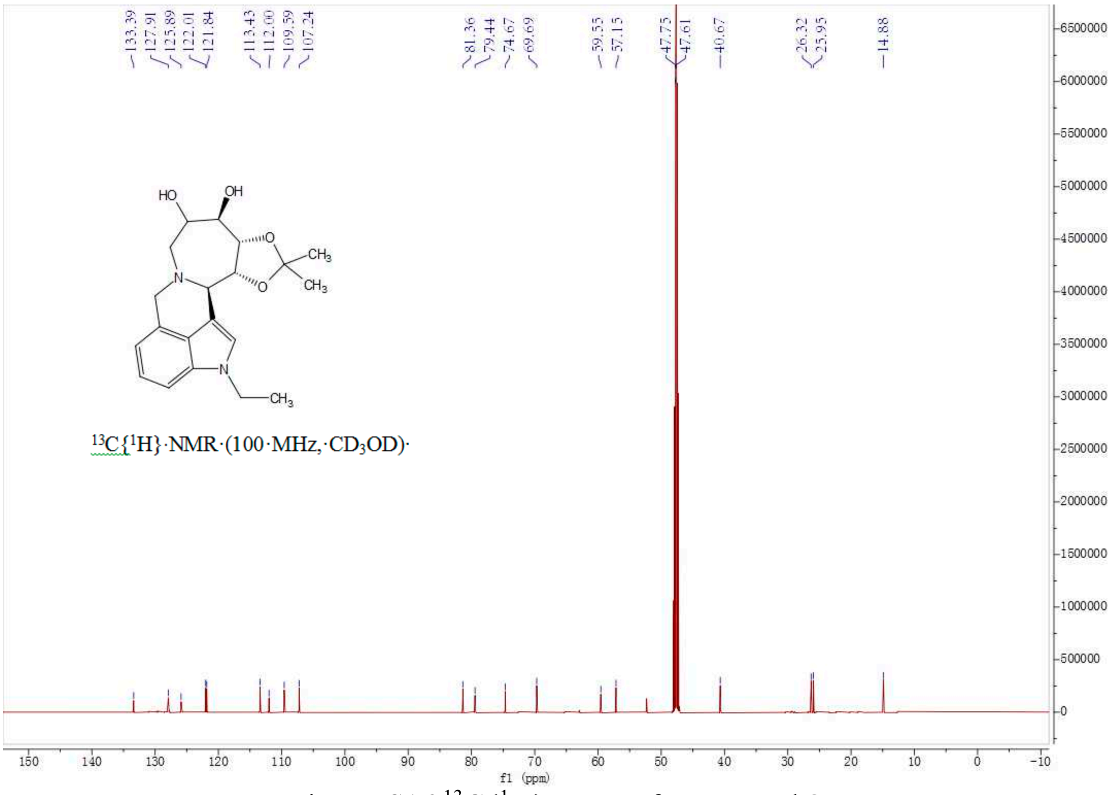

Figure. S16 ^{13}C{^{1}H} NMR of compound **8a**

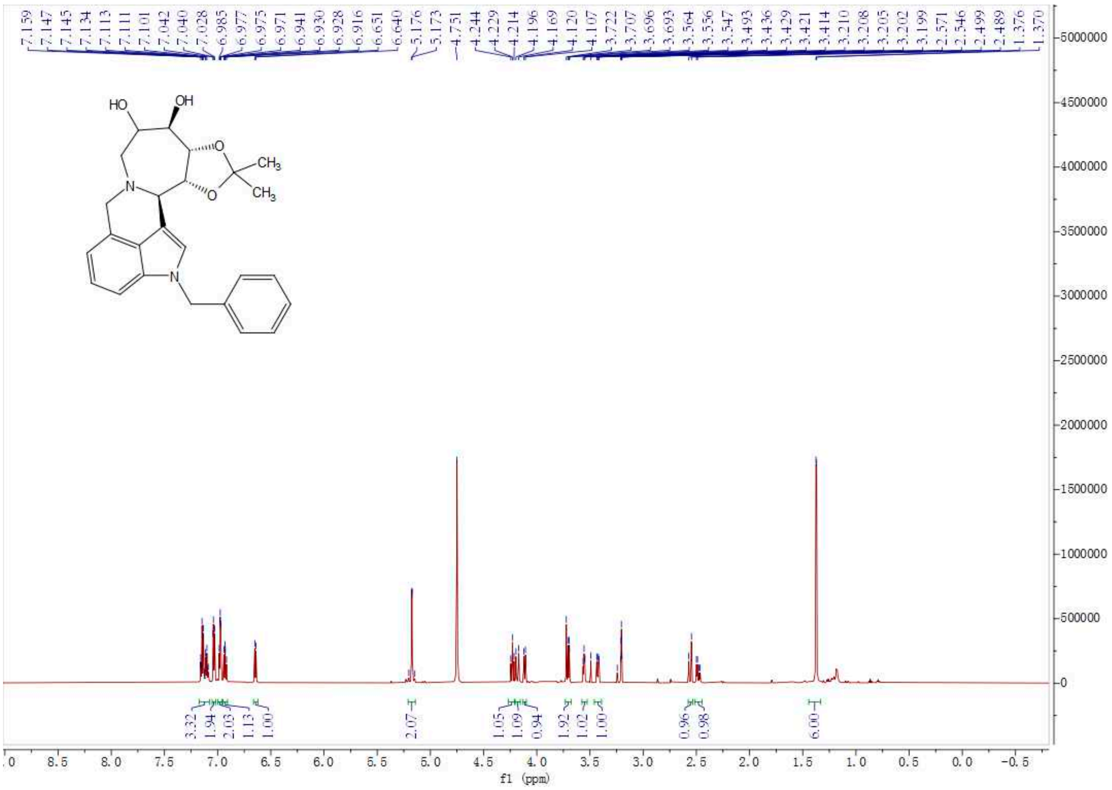

Figure. S17 ^{1}H NMR of compound **9a**

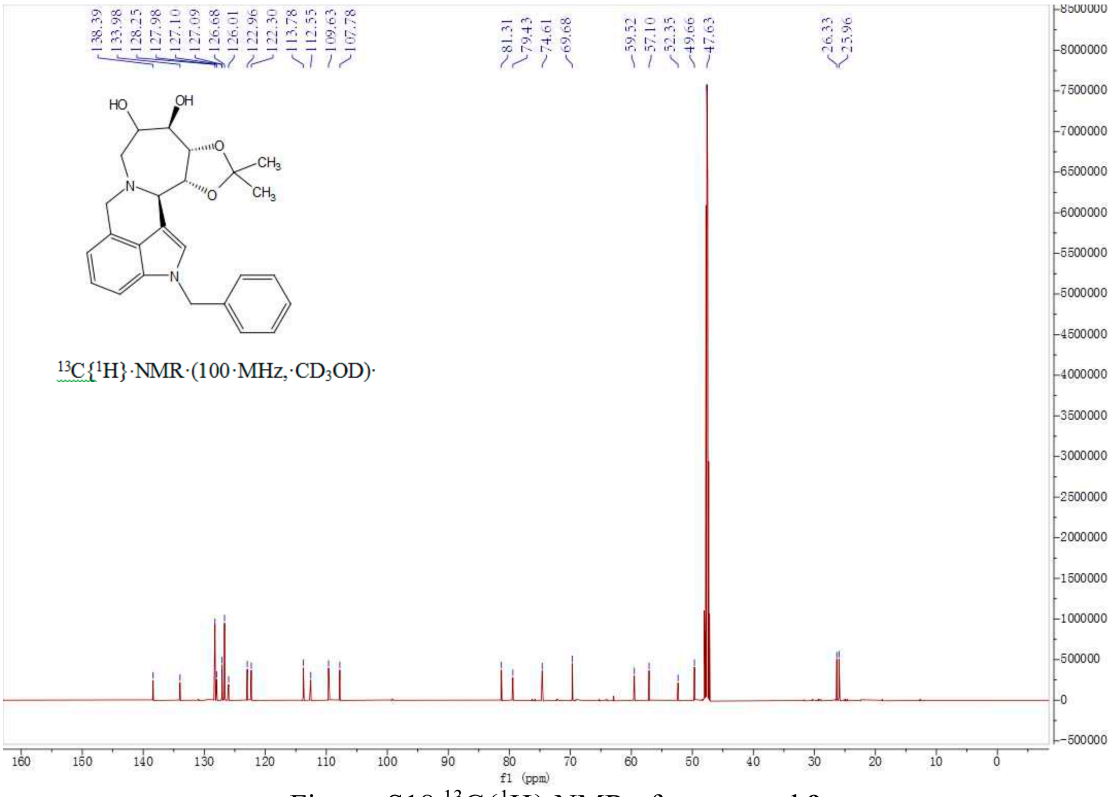

Figure. S18 ^{13}C{^{1}H} NMR of compound **9a**

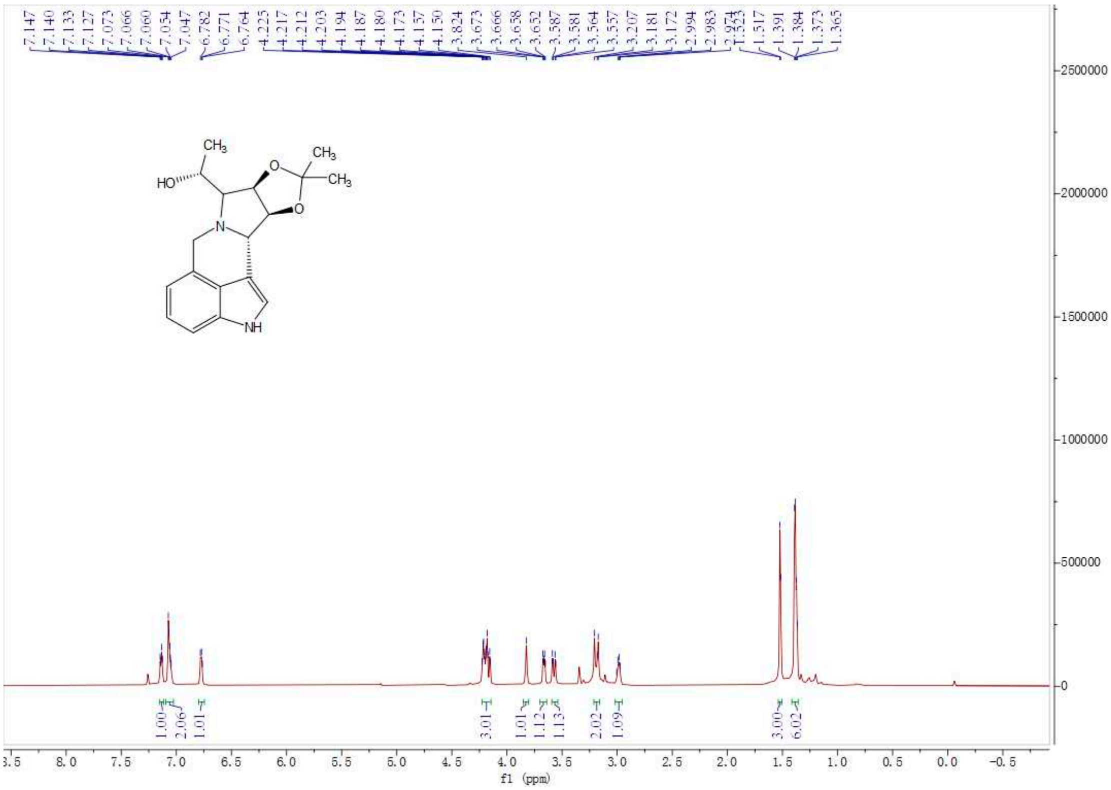

Figure. S19 ^{1}H NMR of compound **10a**

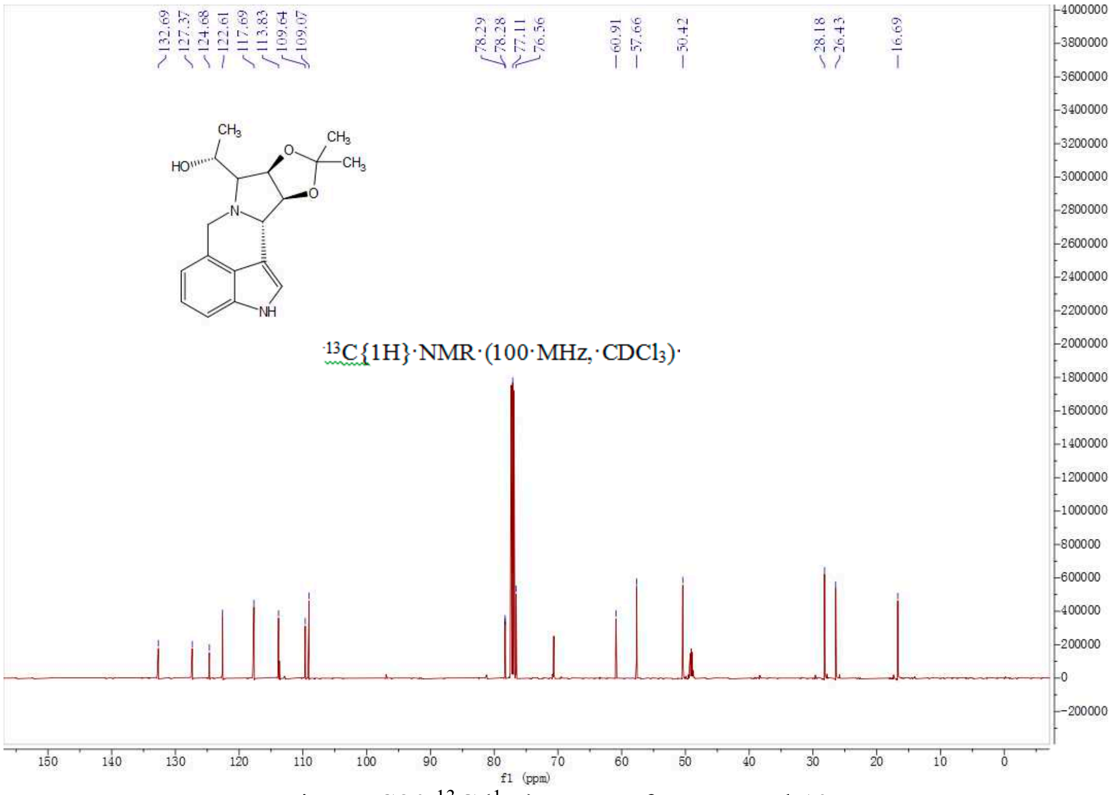

Figure. S20 ^{13}C{^{1}H} NMR of compound **10a**

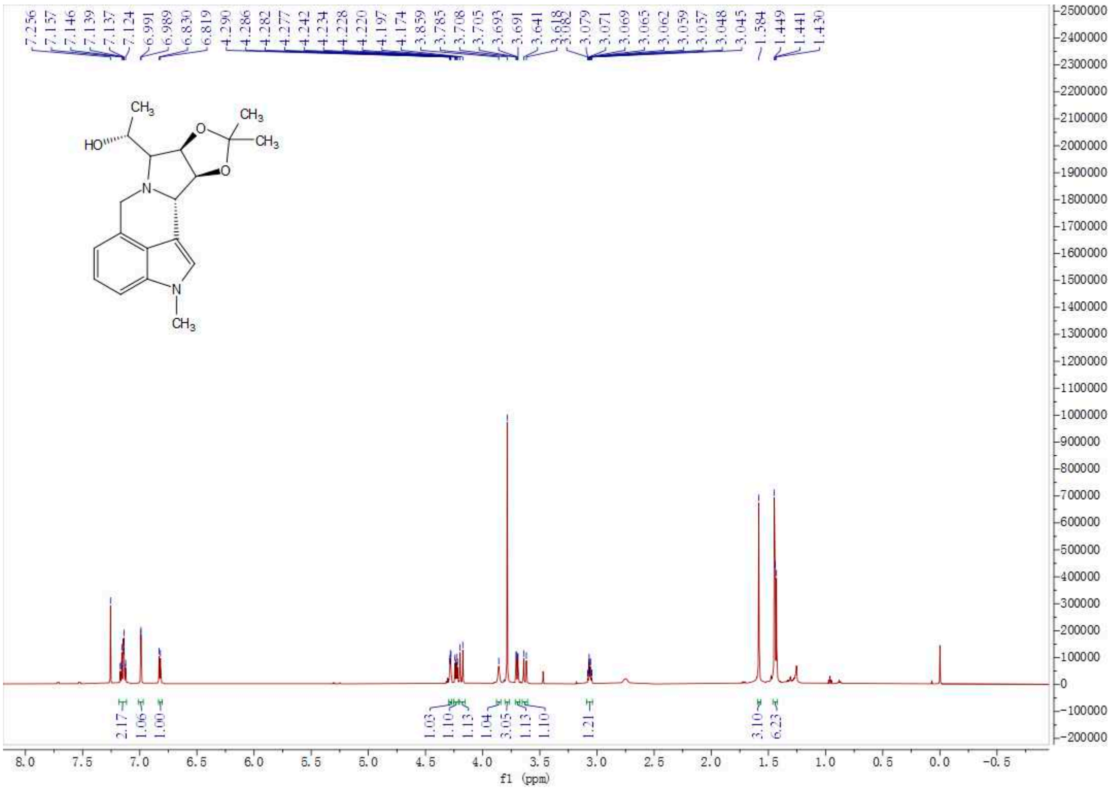

Figure. S21 ^{1}H NMR of compound **11a**

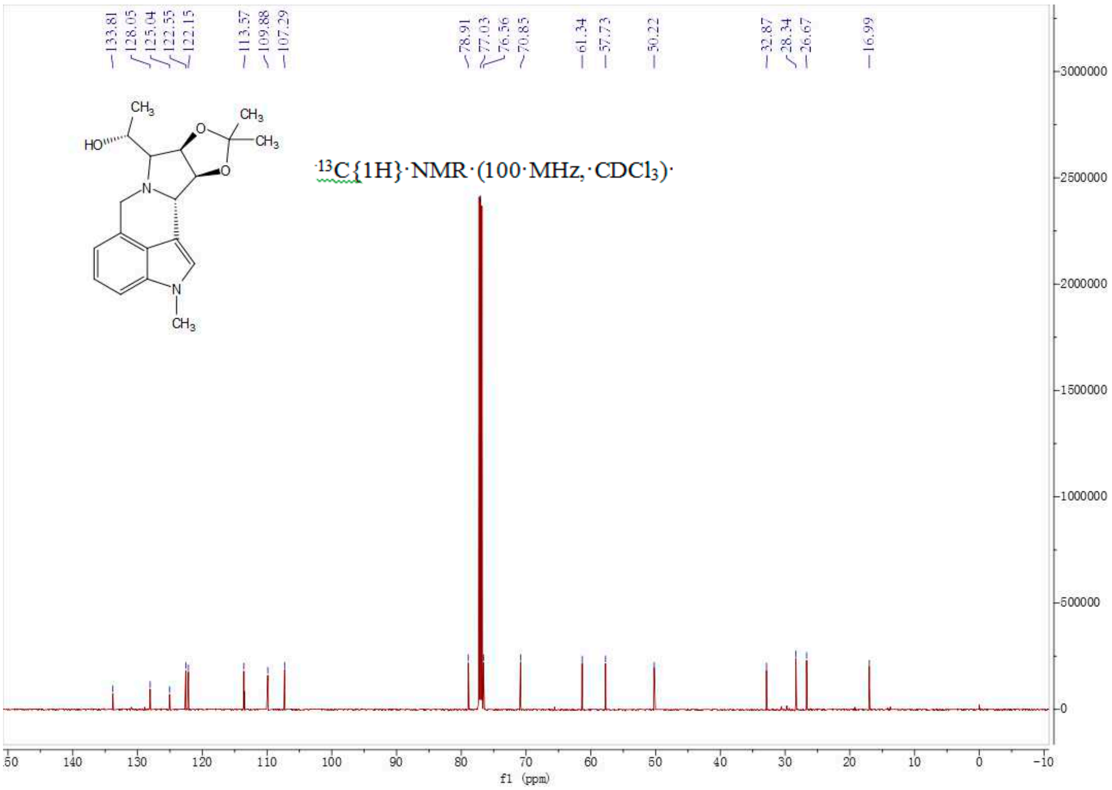

Figure. S22 ^{13}C{^{1}H} NMR of compound **11a**

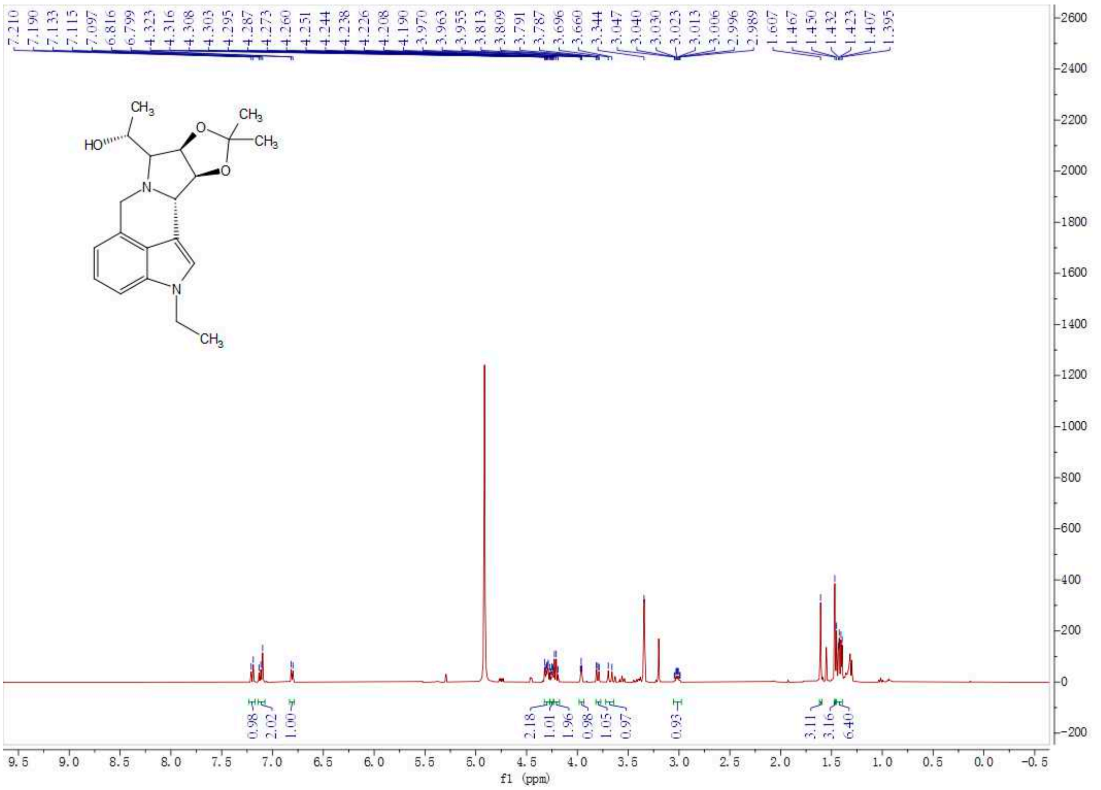

Figure. S23 ^{1}H NMR of compound **12a**

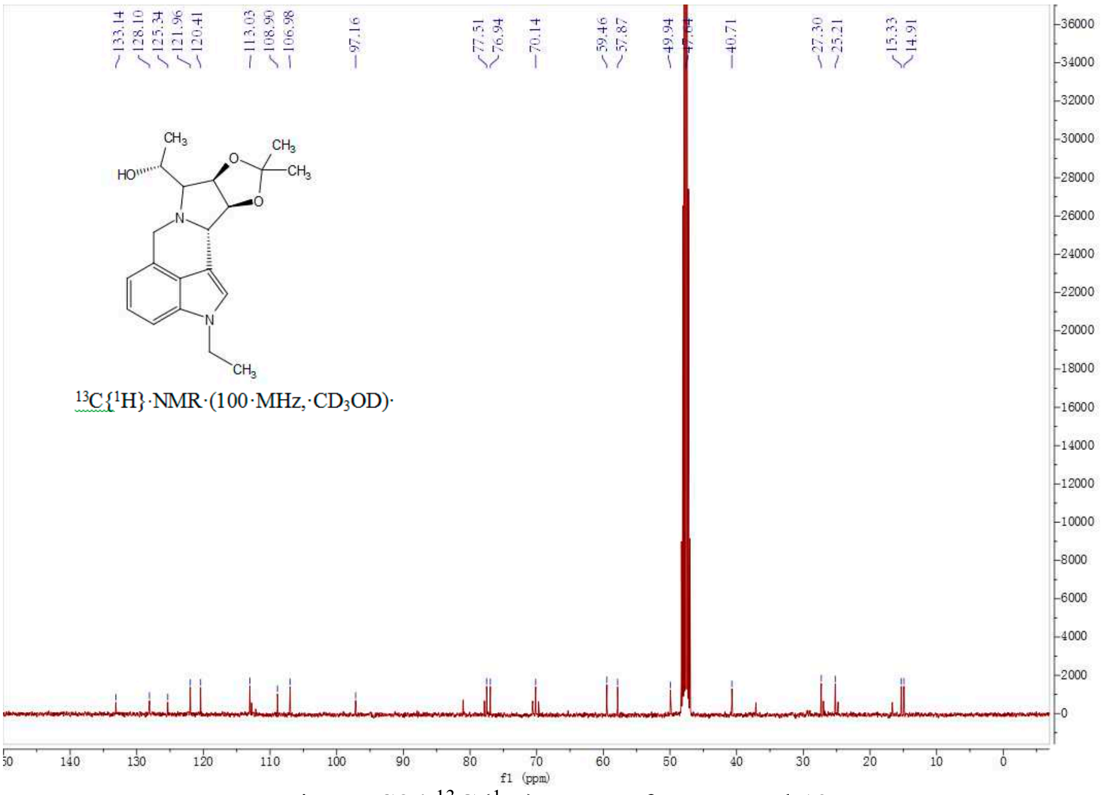

Figure. S24 ^{13}C{^{1}H} NMR of compound **12a**

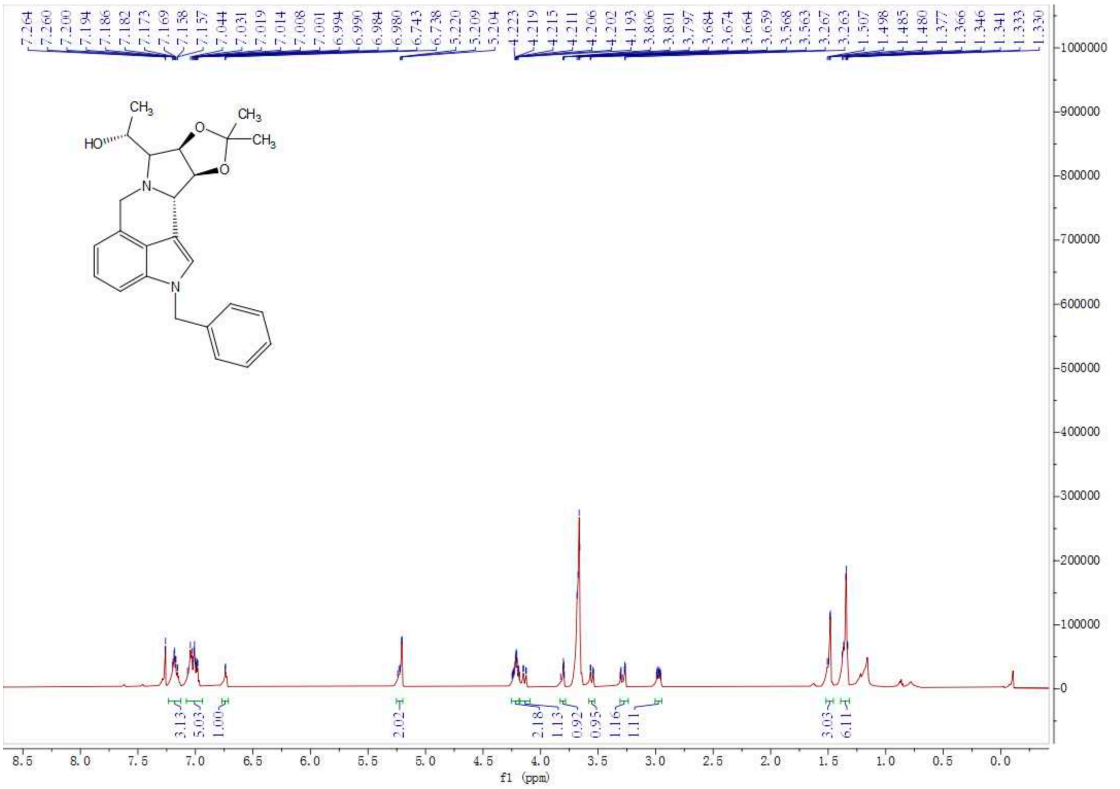

Figure. S25 ^{1}H NMR of compound **13a**

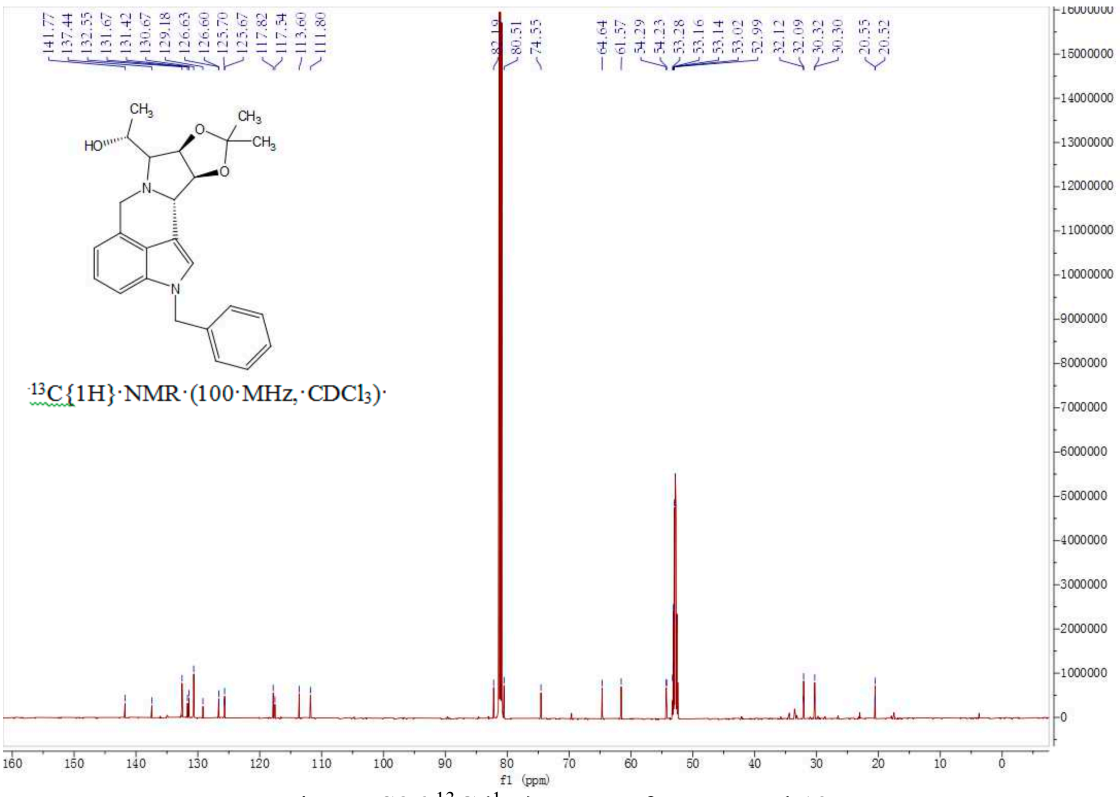

Figure. S26 ^{13}C{^{1}H} NMR of compound **13a**

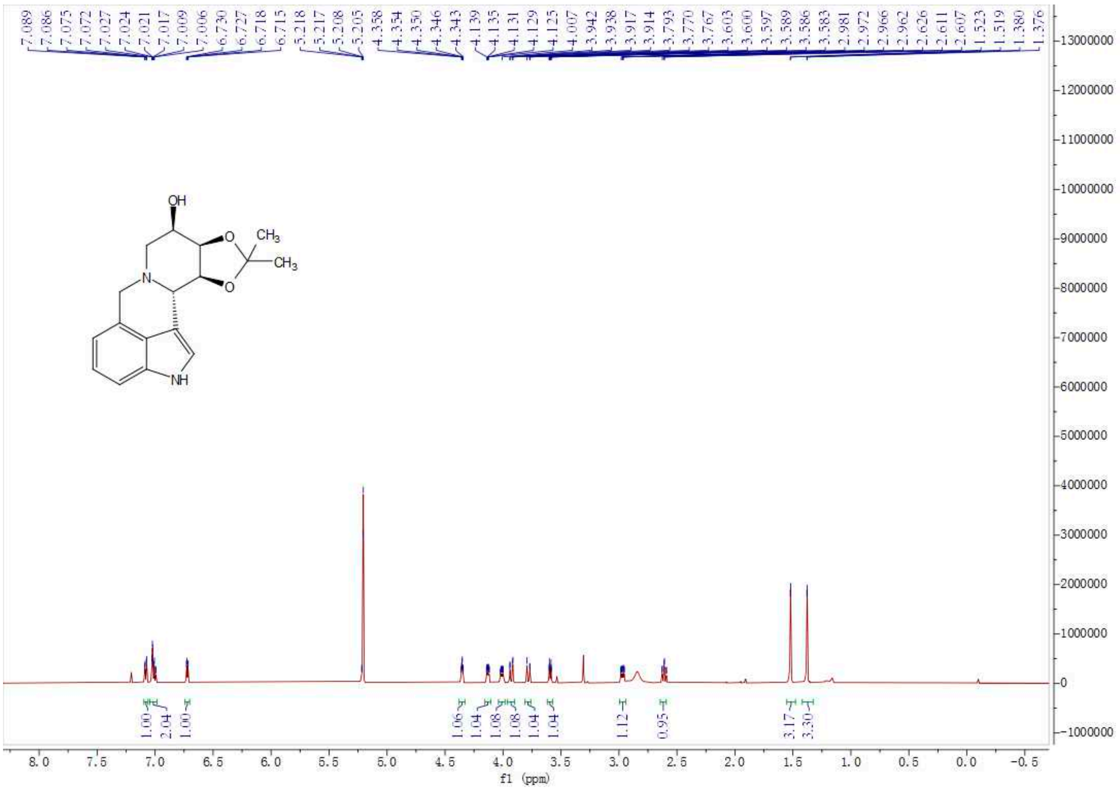

Figure. S27 ^{1}H NMR of compound **14a**

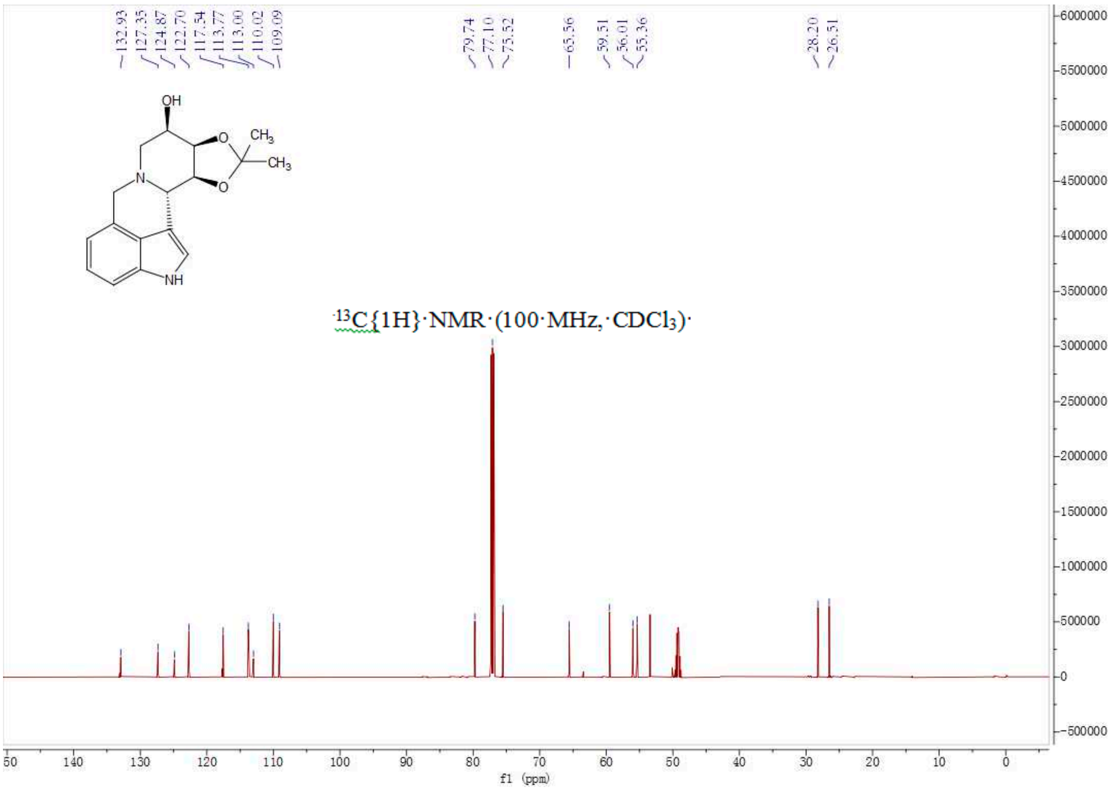

Figure. S28 ^{13}C{^{1}H} NMR of compound **14a**

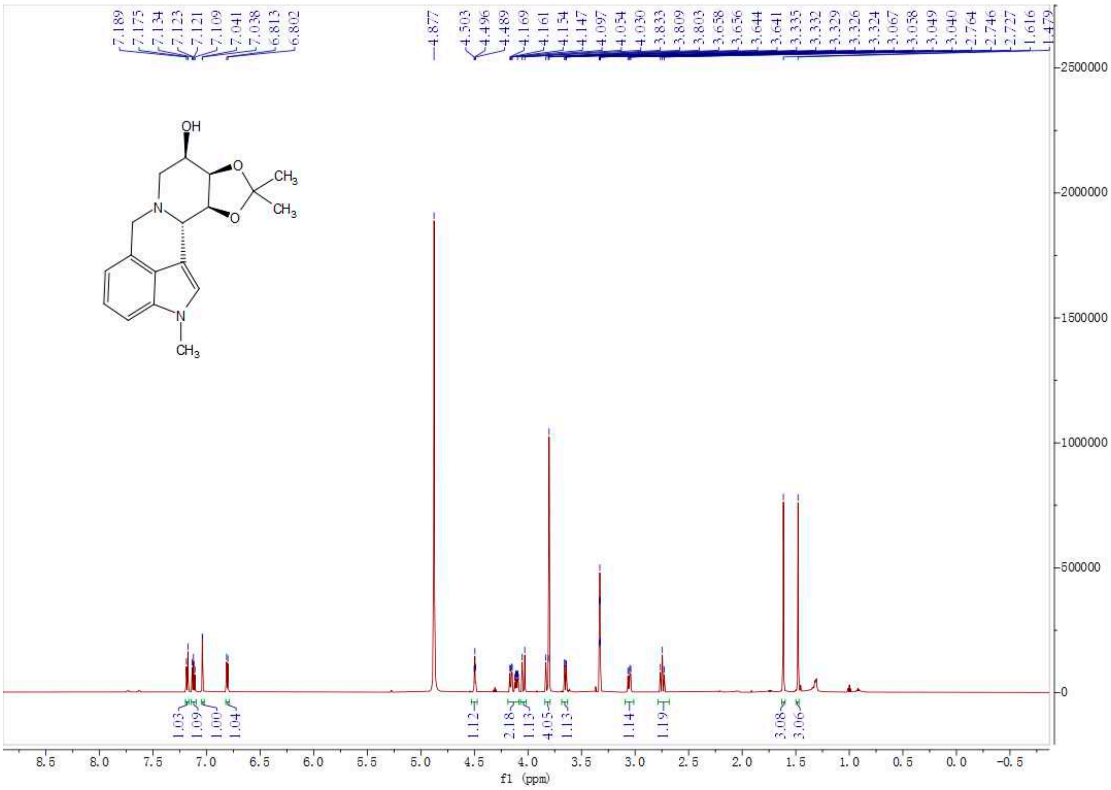

Figure. S29 ^{1}H NMR of compound **15a**

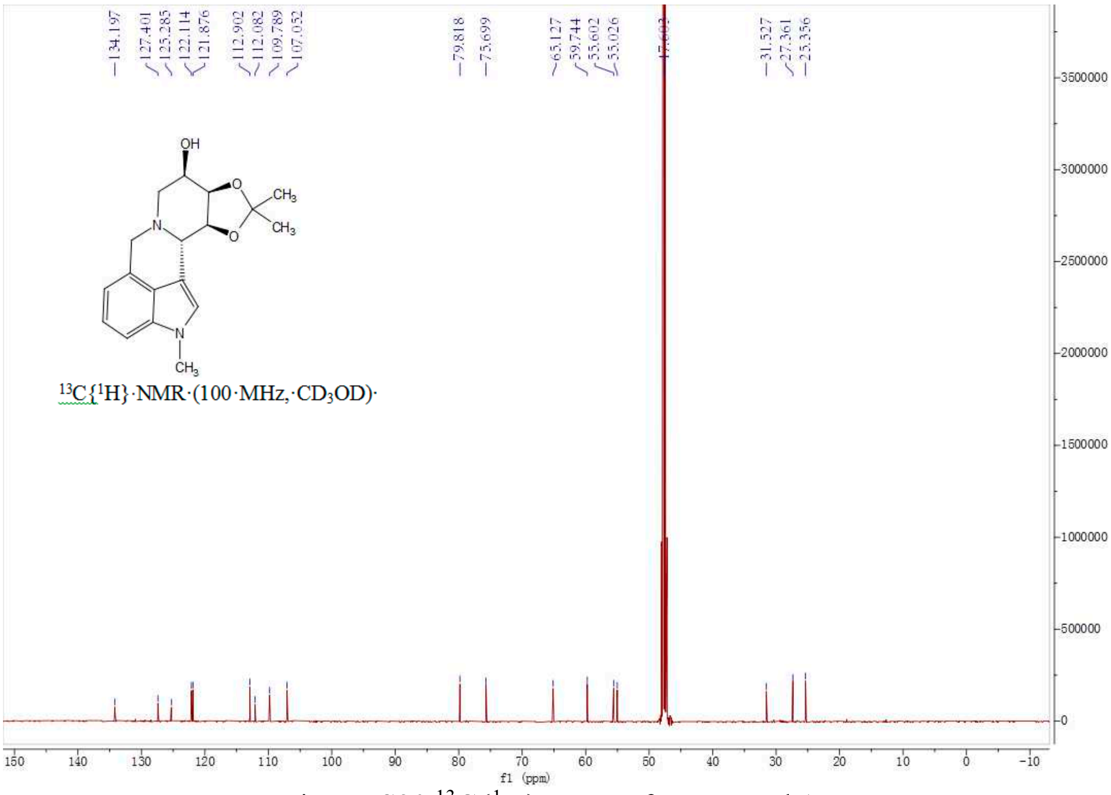

Figure. S30 ^{13}C{^{1}H} NMR of compound **15a**

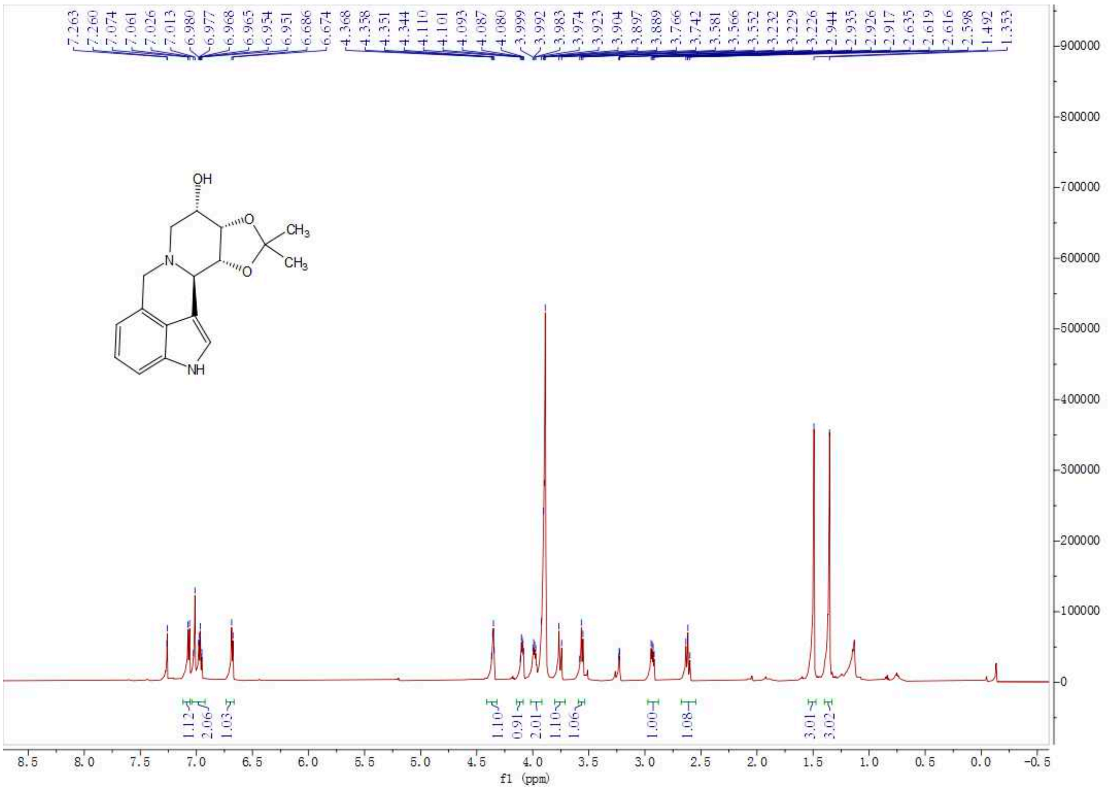

Figure. S31 ^{1}H NMR of compound **16a**

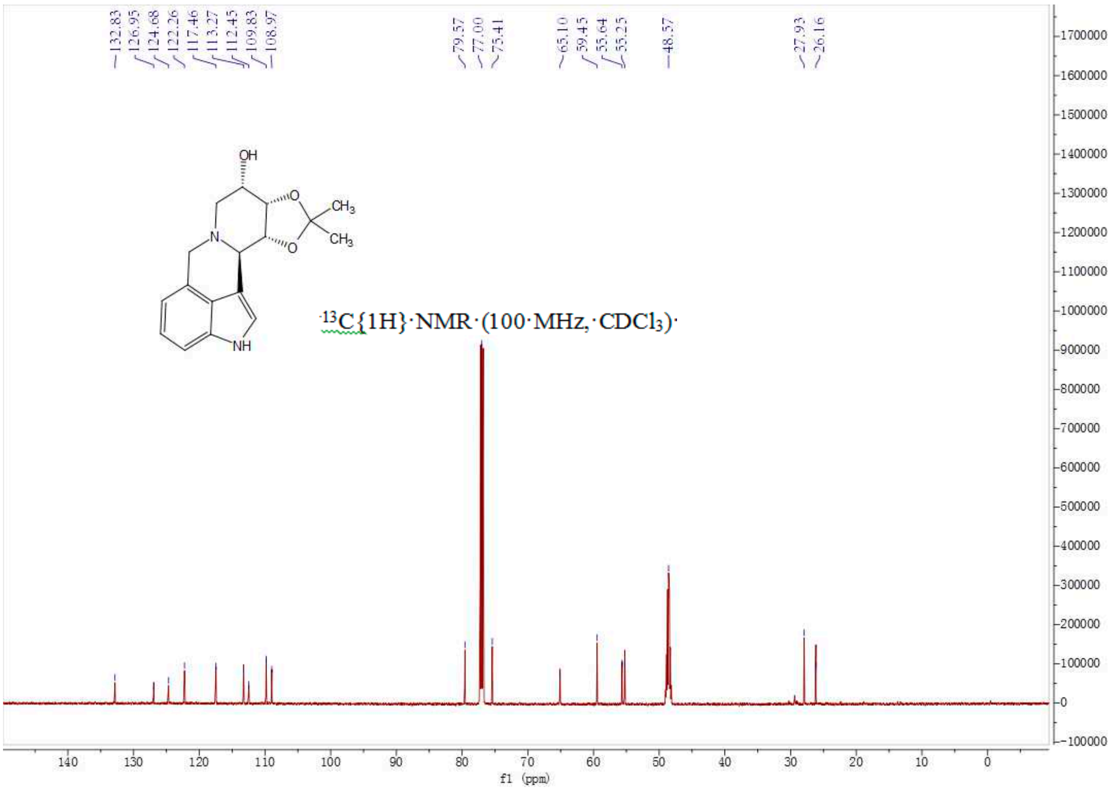

Figure. S32 $^{13}C\{^{1}H\}$ NMR of compound **16a**

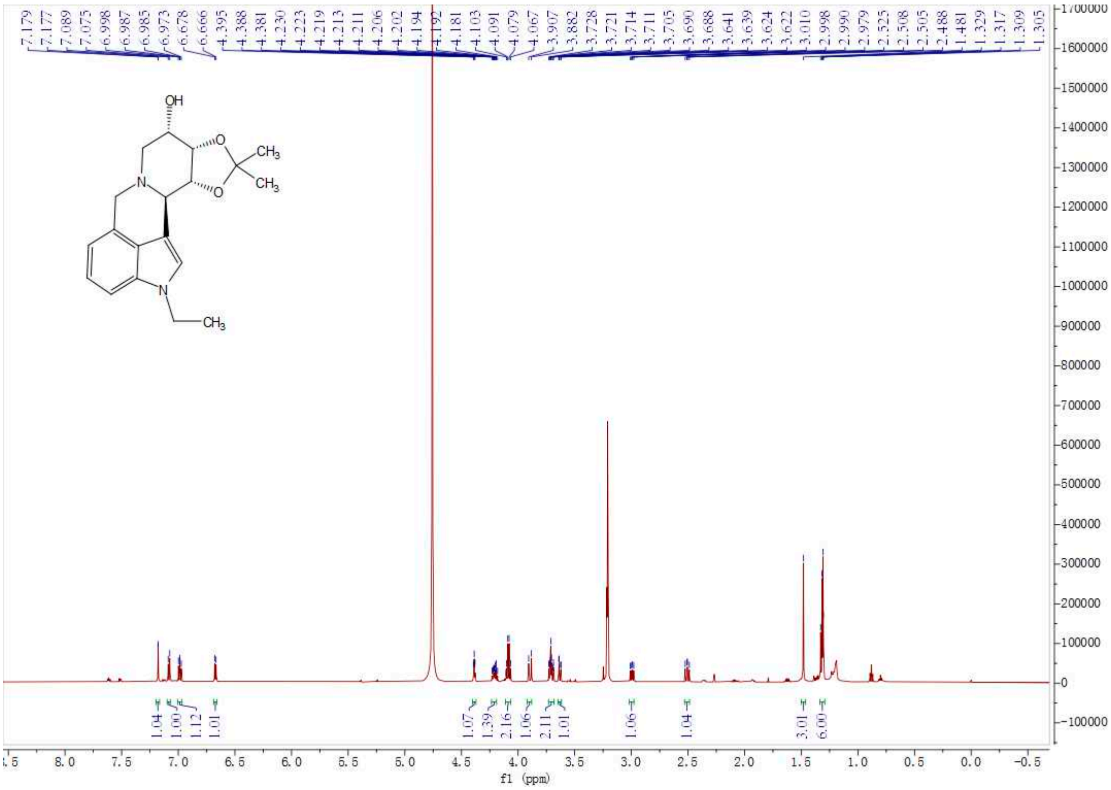

Figure. S33 ^{1}H NMR of compound **17a**

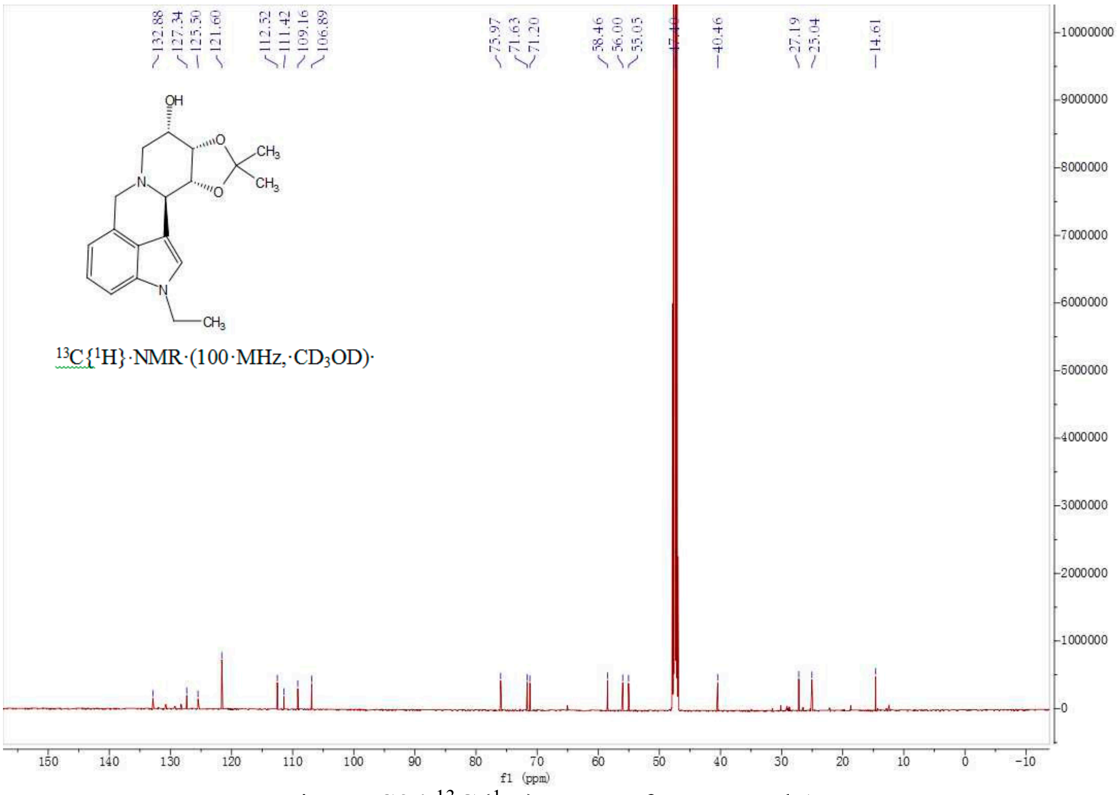

Figure. S34 $^{13}C\{^{1}H\}$ NMR of compound **17a**

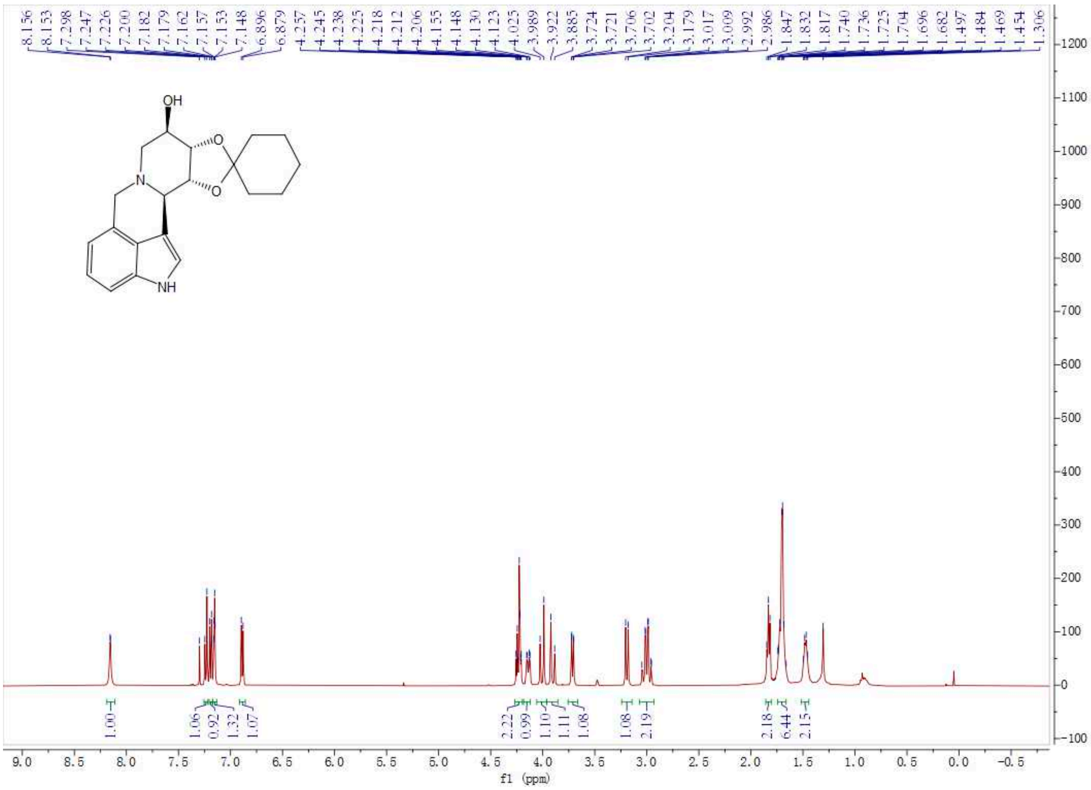

Figure. S35 ^{1}H NMR of compound **18a**

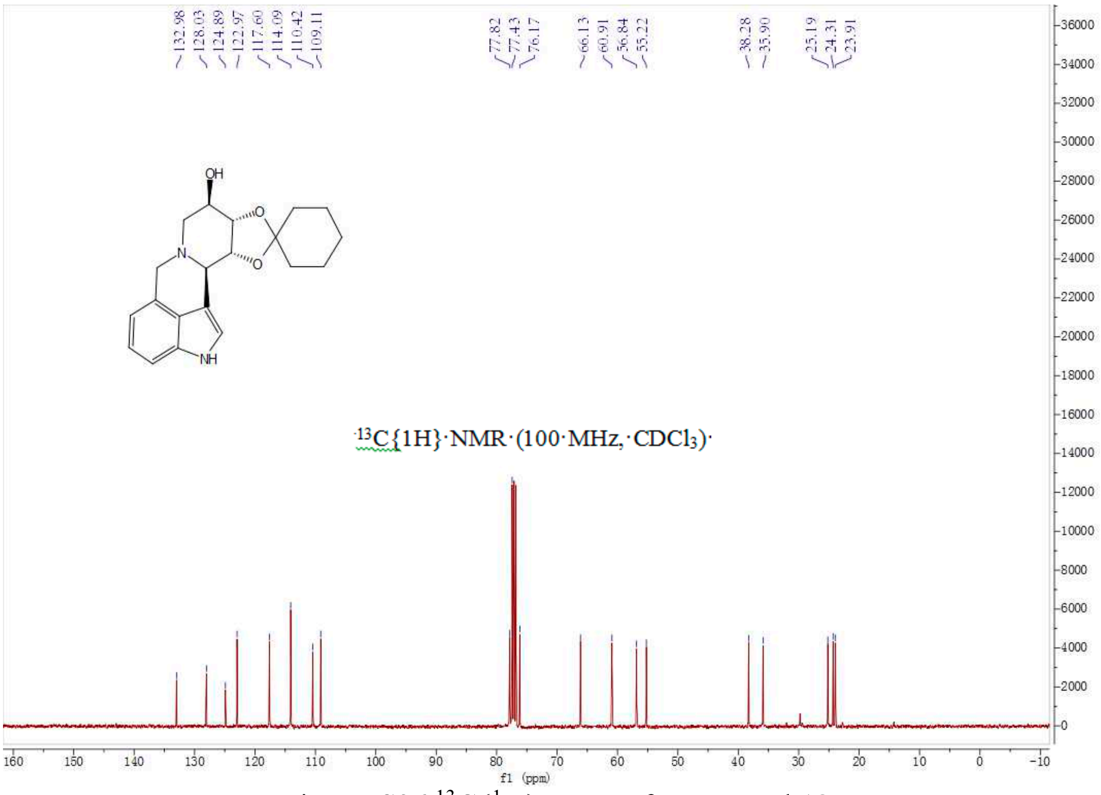

Figure. S36 ^{13}C{^{1}H} NMR of compound **18a**

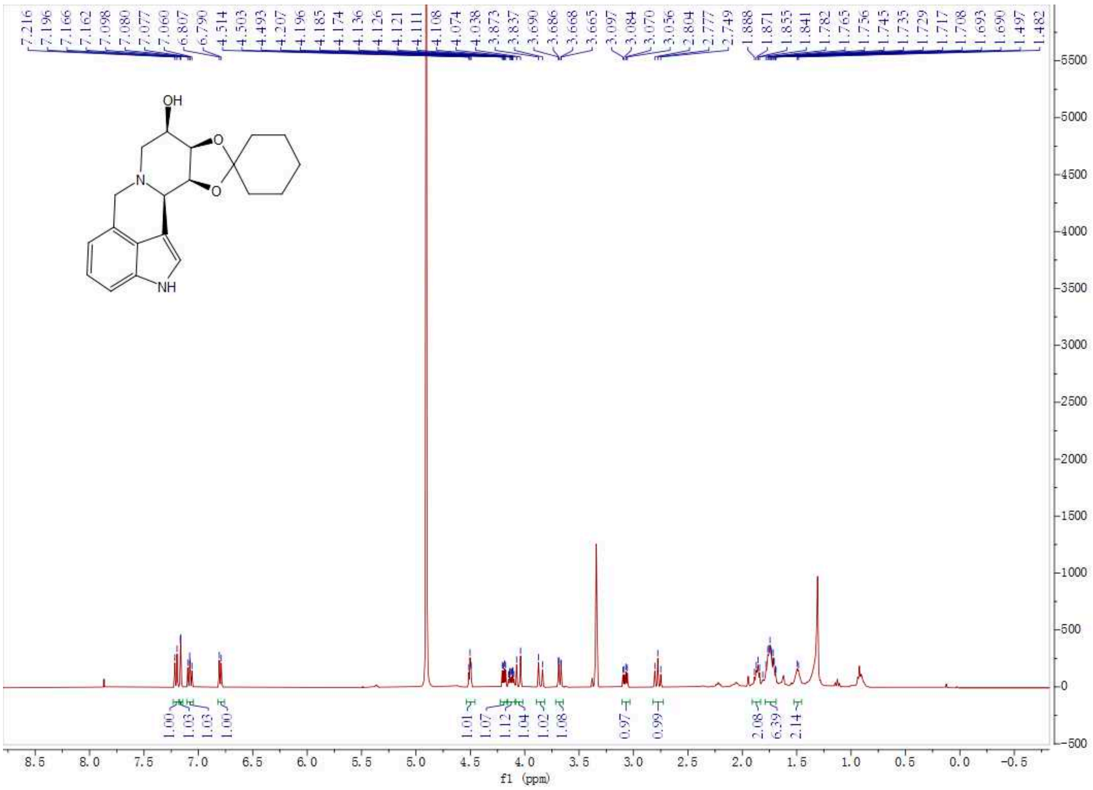

Figure. S37 ^{1}H NMR of compound **19a**

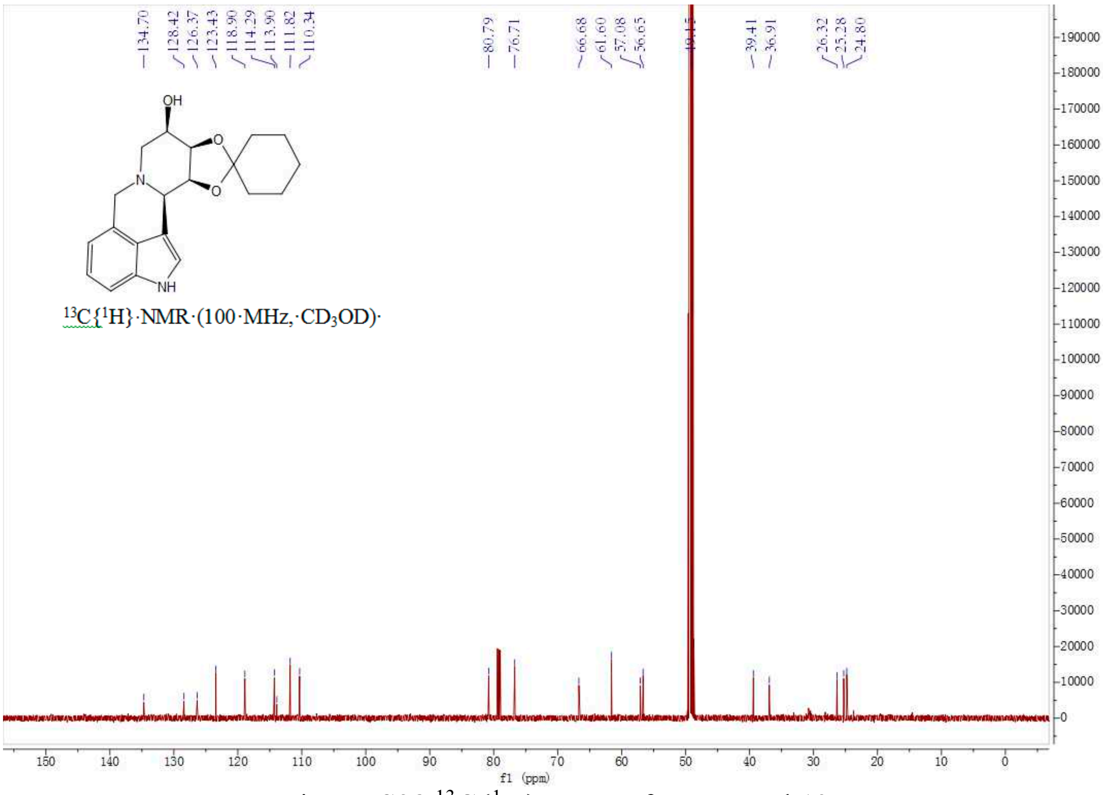

Figure. S38 $^{13}C\{^{1}H\}$ NMR of compound **19a**

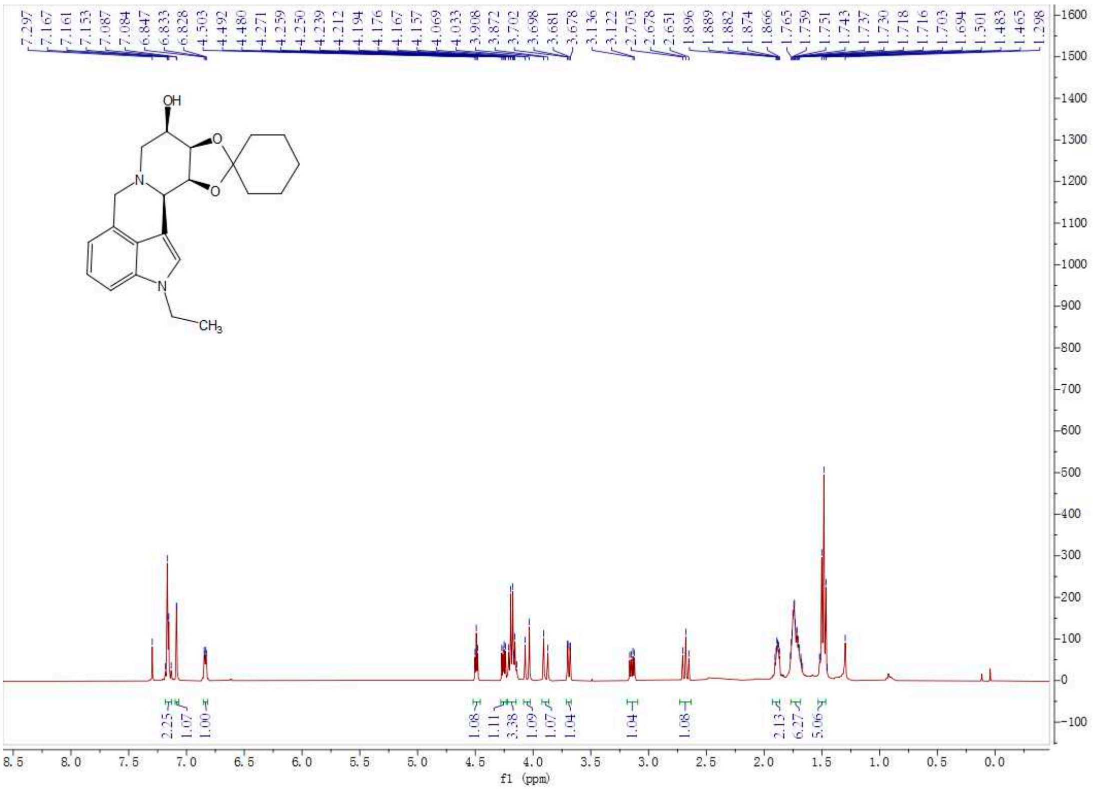

Figure. S39 ^{1}H NMR of compound **20a**

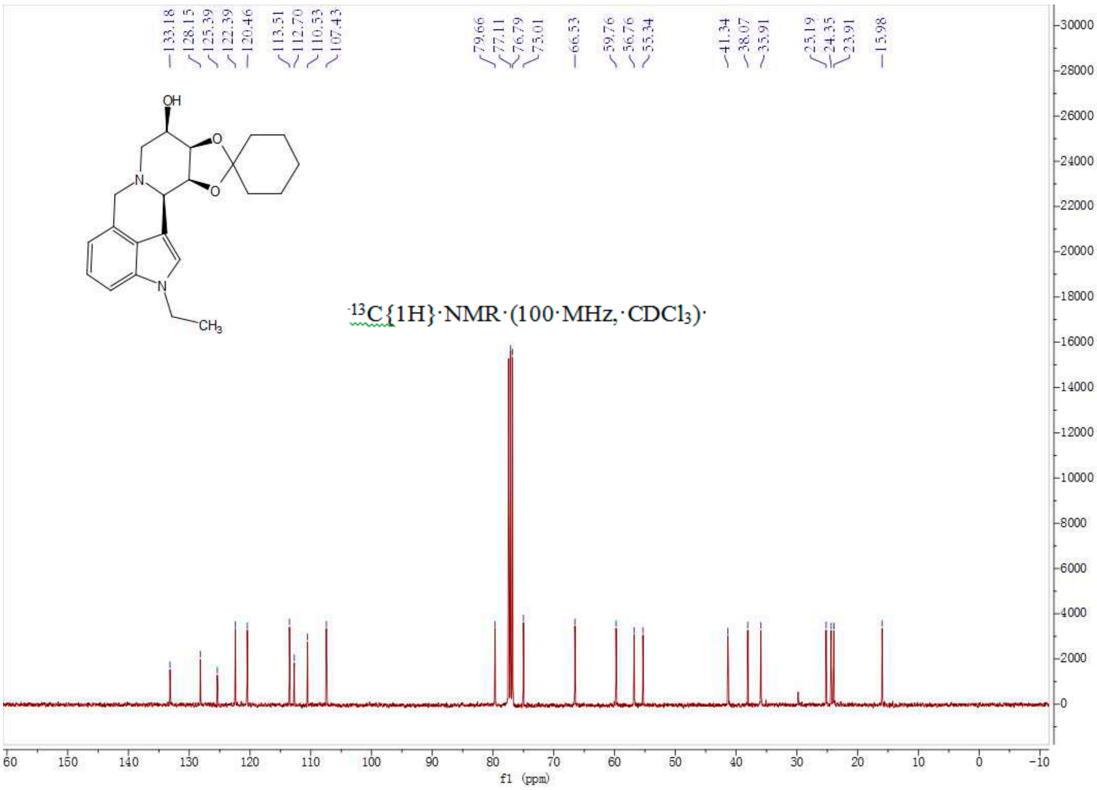

Figure. S40 $^{13}C\{^{1}H\}$ NMR of compound **20a**

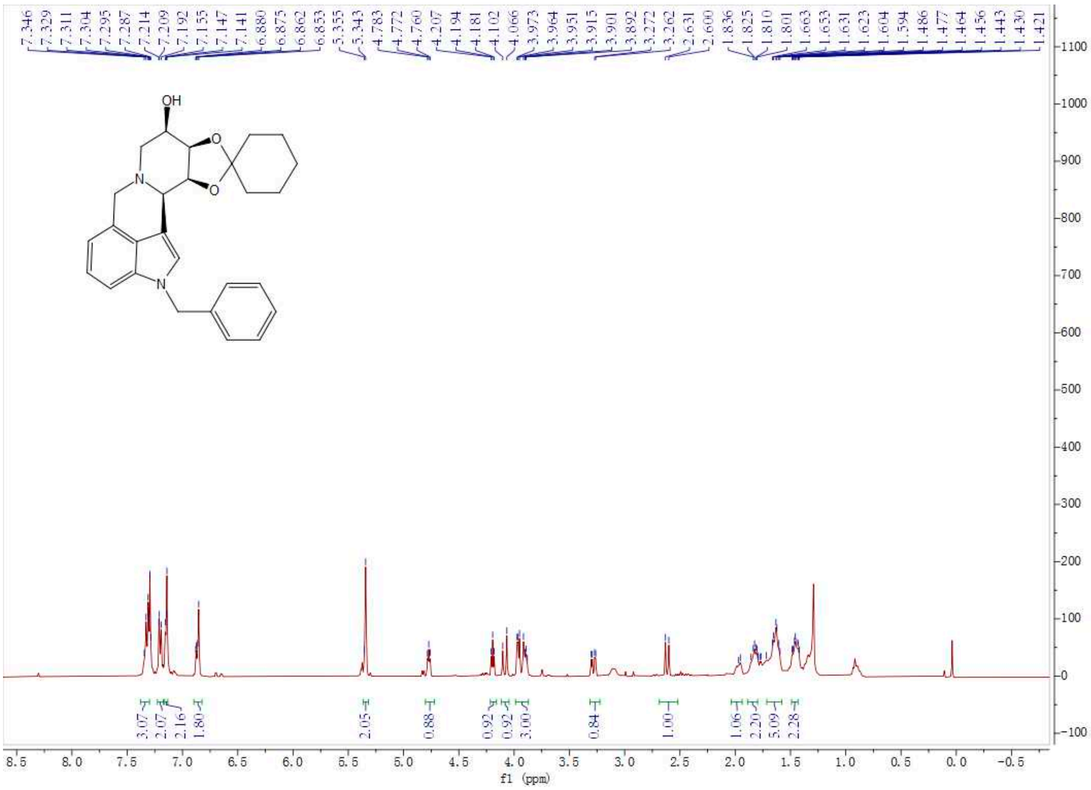

Figure. S41 ^{1}H NMR of compound **21a**

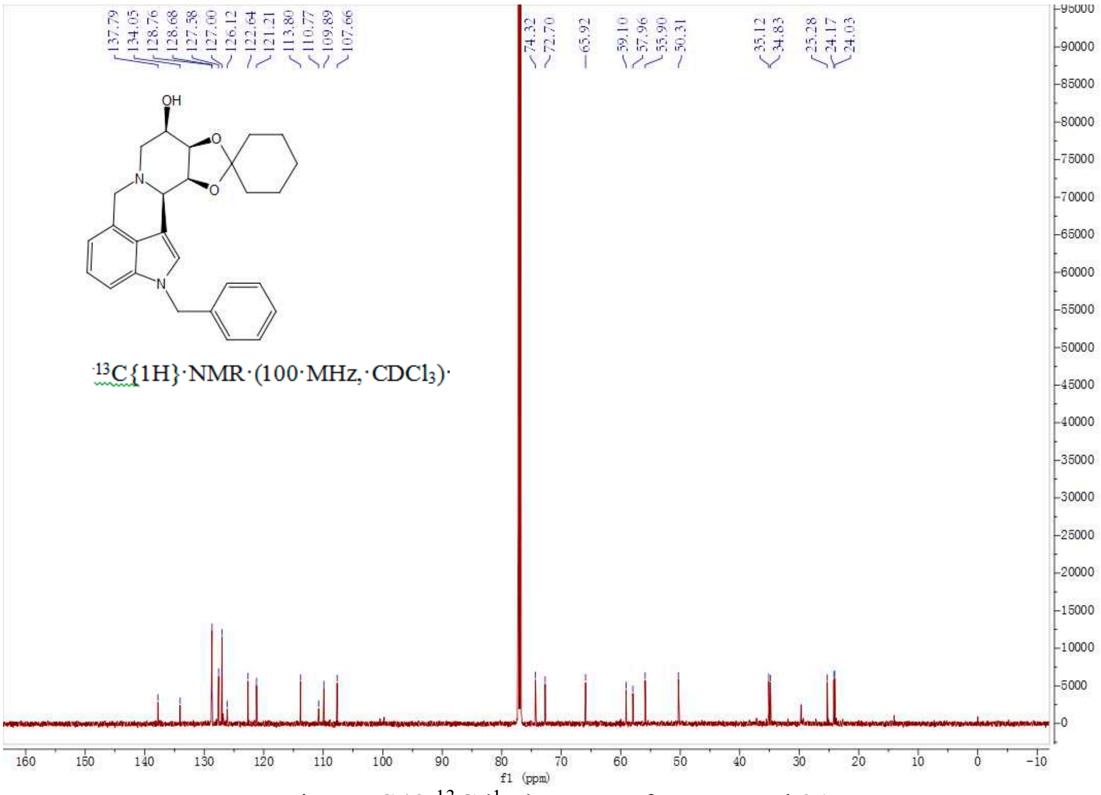

Figure. S42 $^{13}C\{^{1}H\}$ NMR of compound **21a**

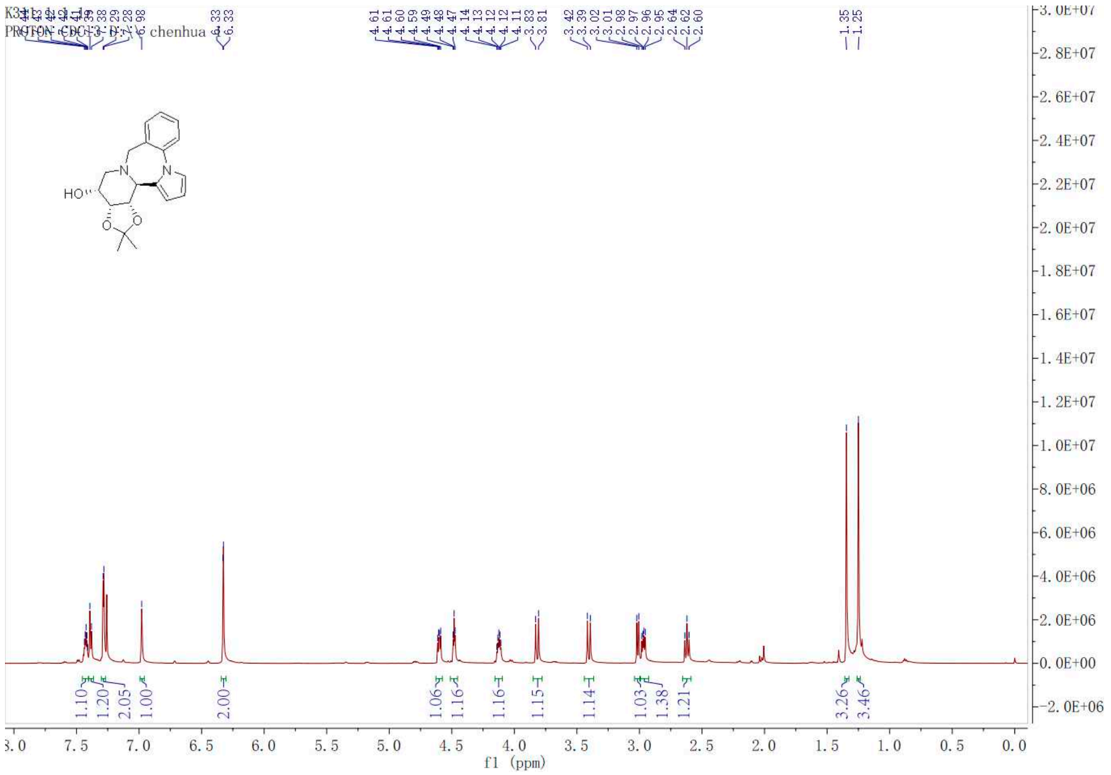

Figure. S43 ^{1}H NMR of compound **1b**

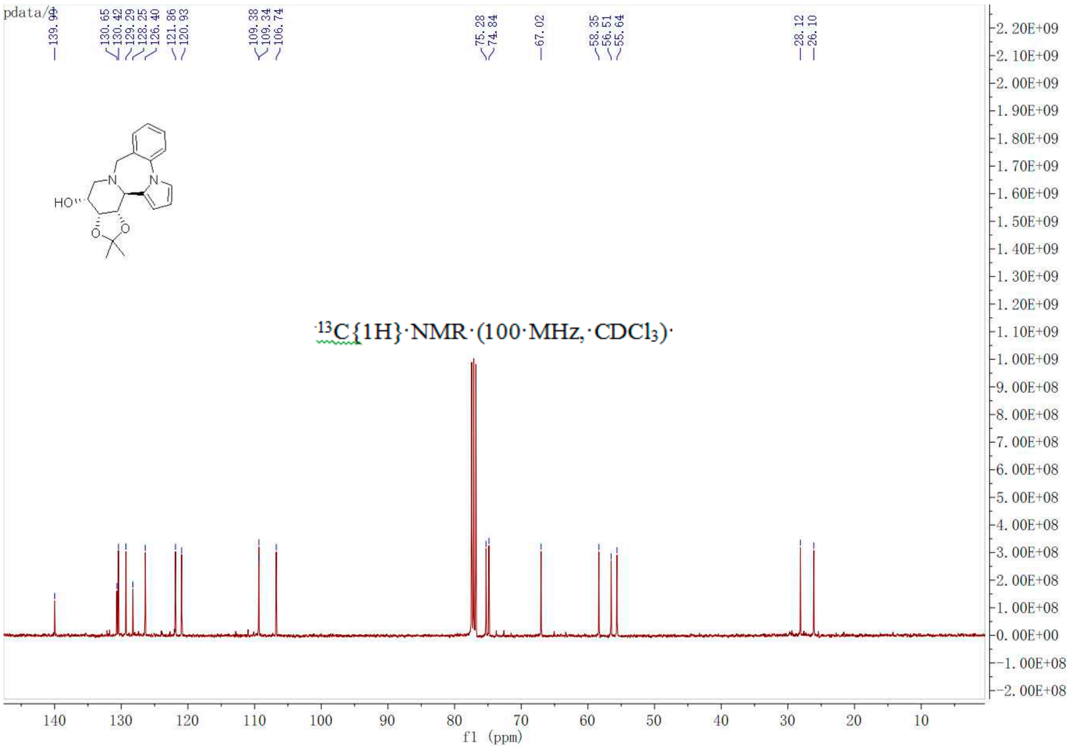

Figure. S44 ^{13}C{^{1}H} NMR of compound **1b**

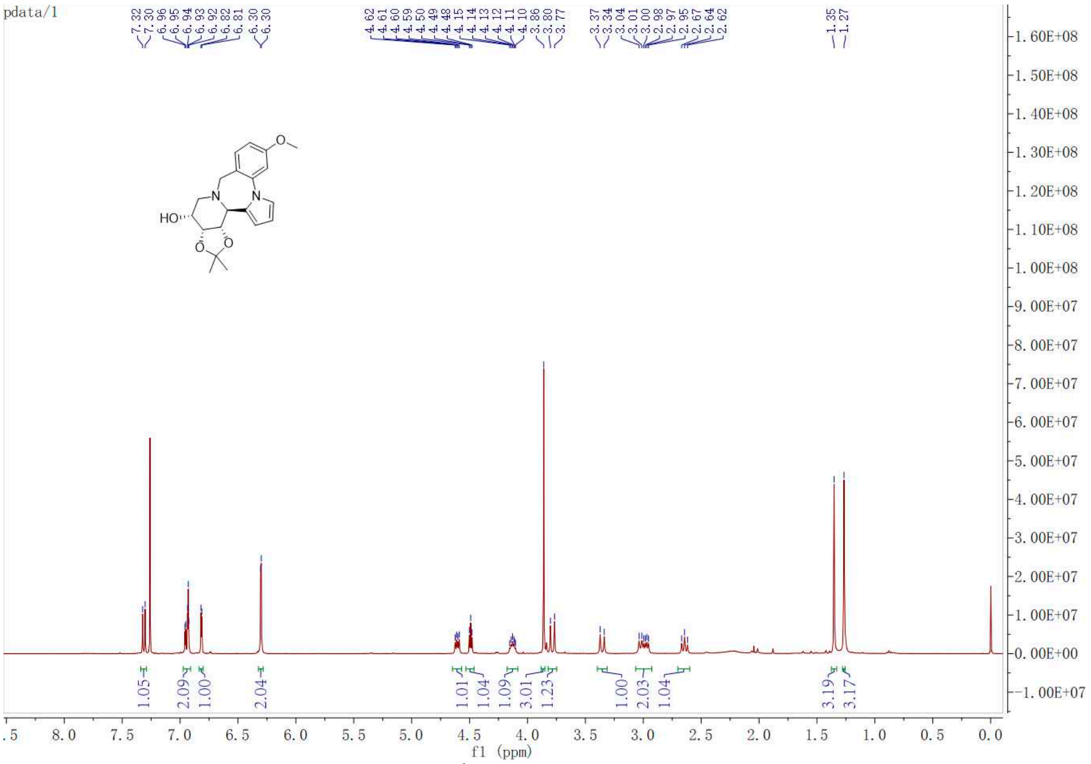

Figure. S45 ^{1}H NMR of compound **2b**

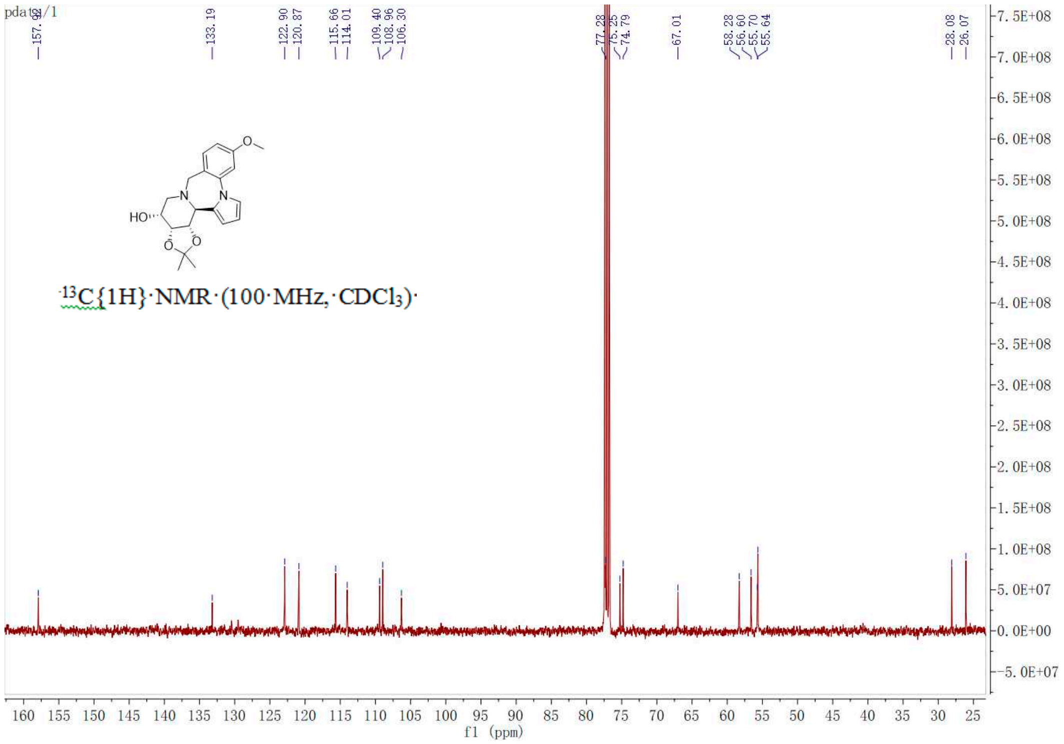

Figure. S46 $^{13}C\{^{1}H\}$ NMR of compound **2b**

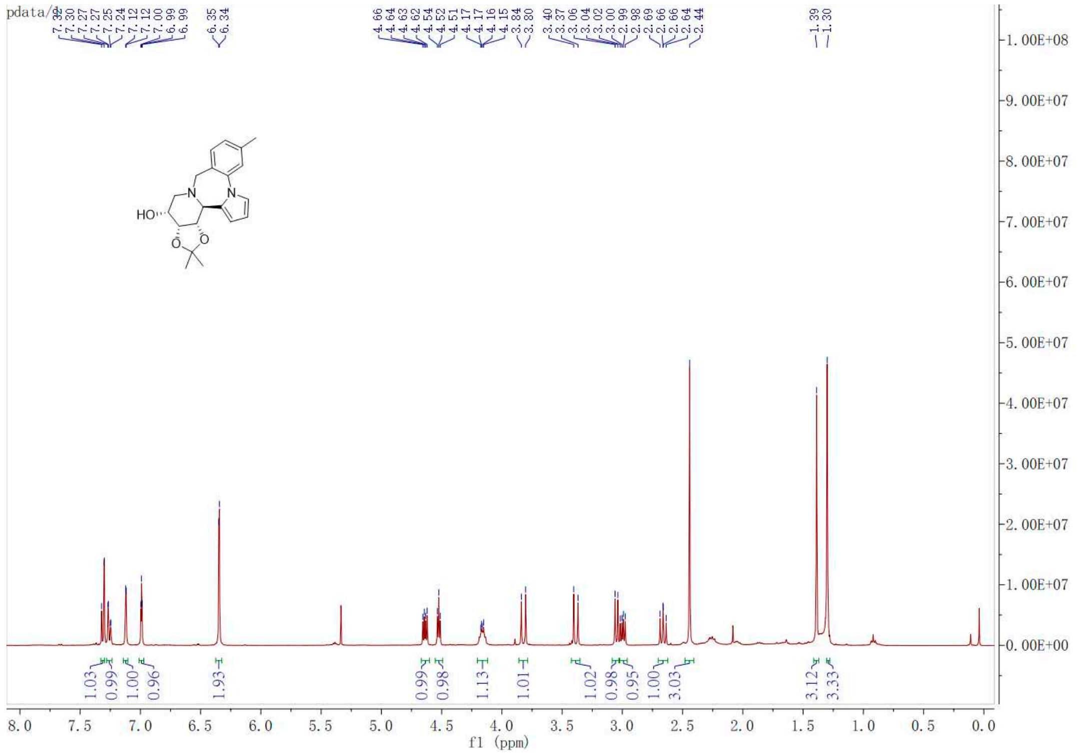

Figure. S47 ^{1}H NMR of compound **3b**

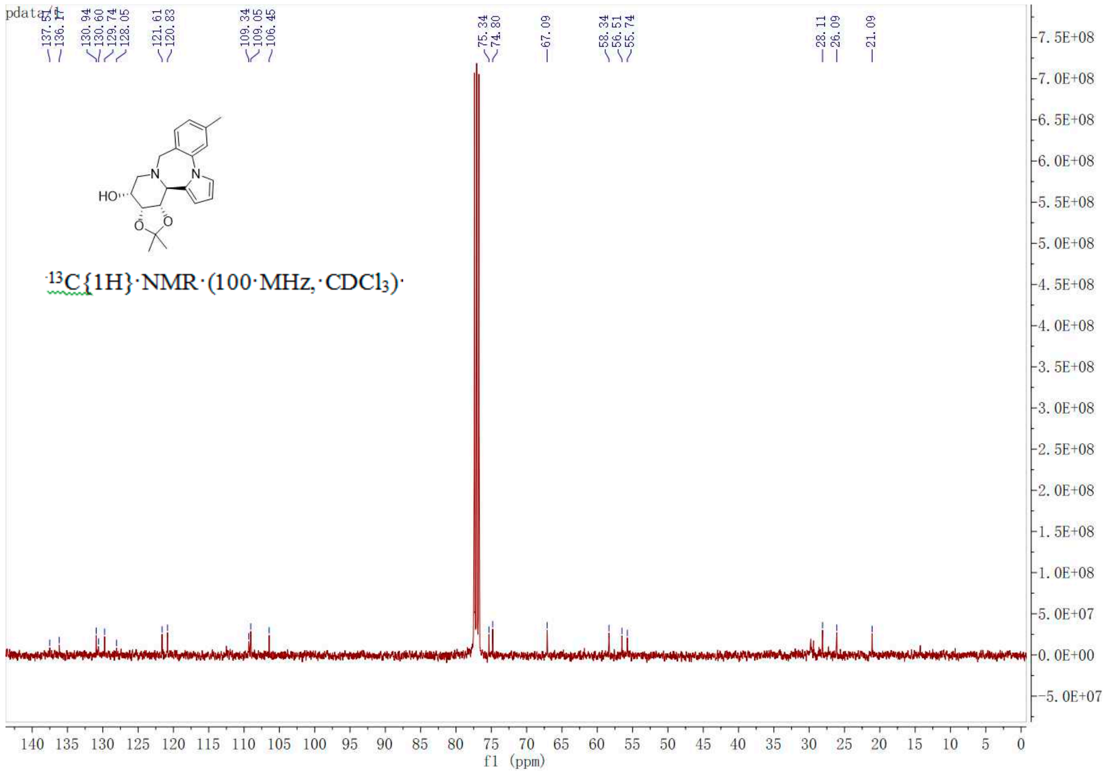

Figure. S48 ^{13}C{^{1}H} NMR of compound **3b**

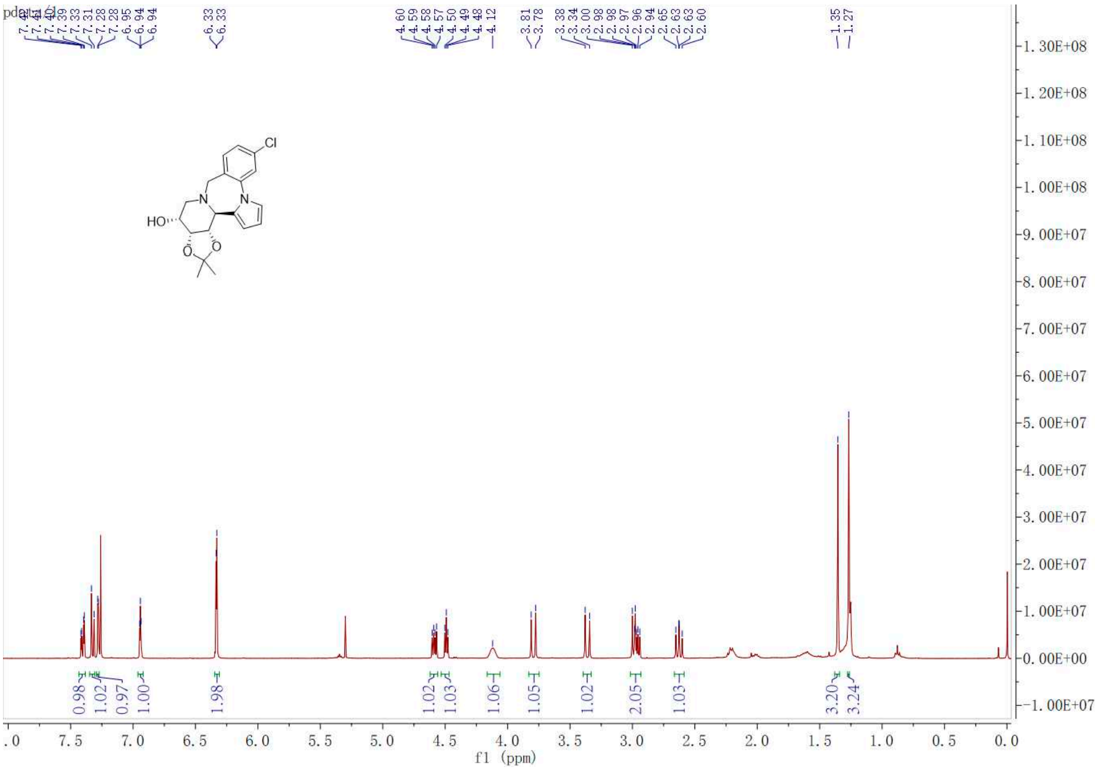

Figure. S49 ^{1}H NMR of compound **4b**

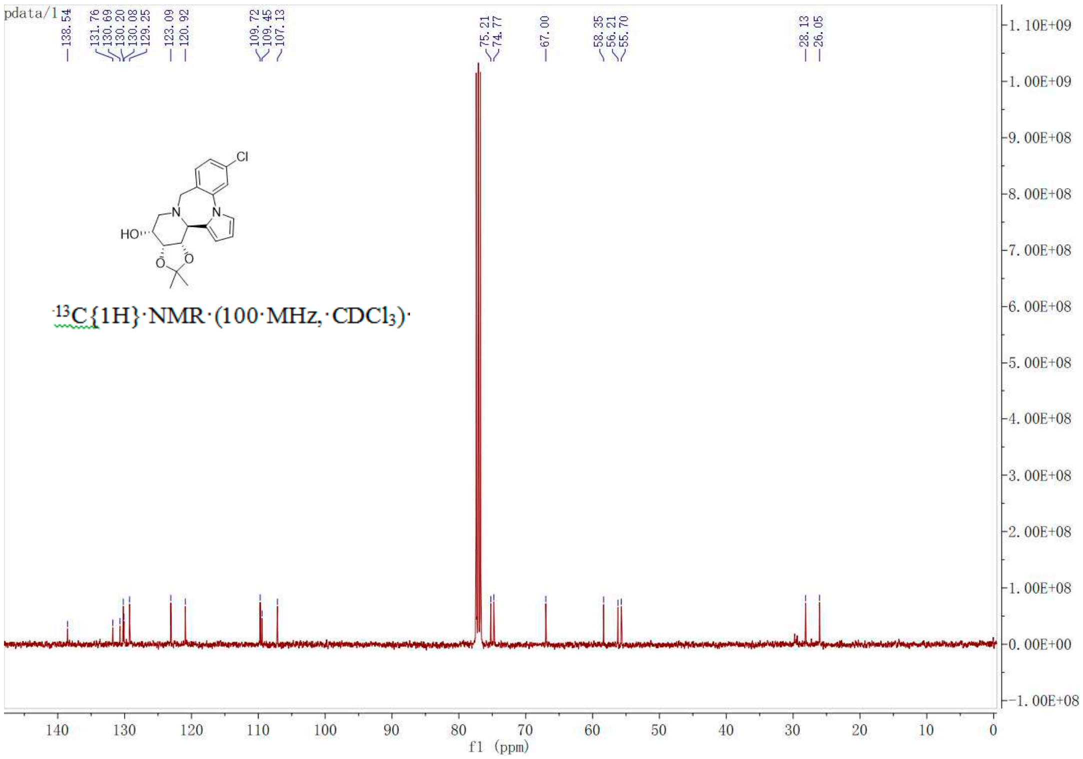

Figure. S50 $^{13}C\{^{1}H\}$ NMR of compound **4b**

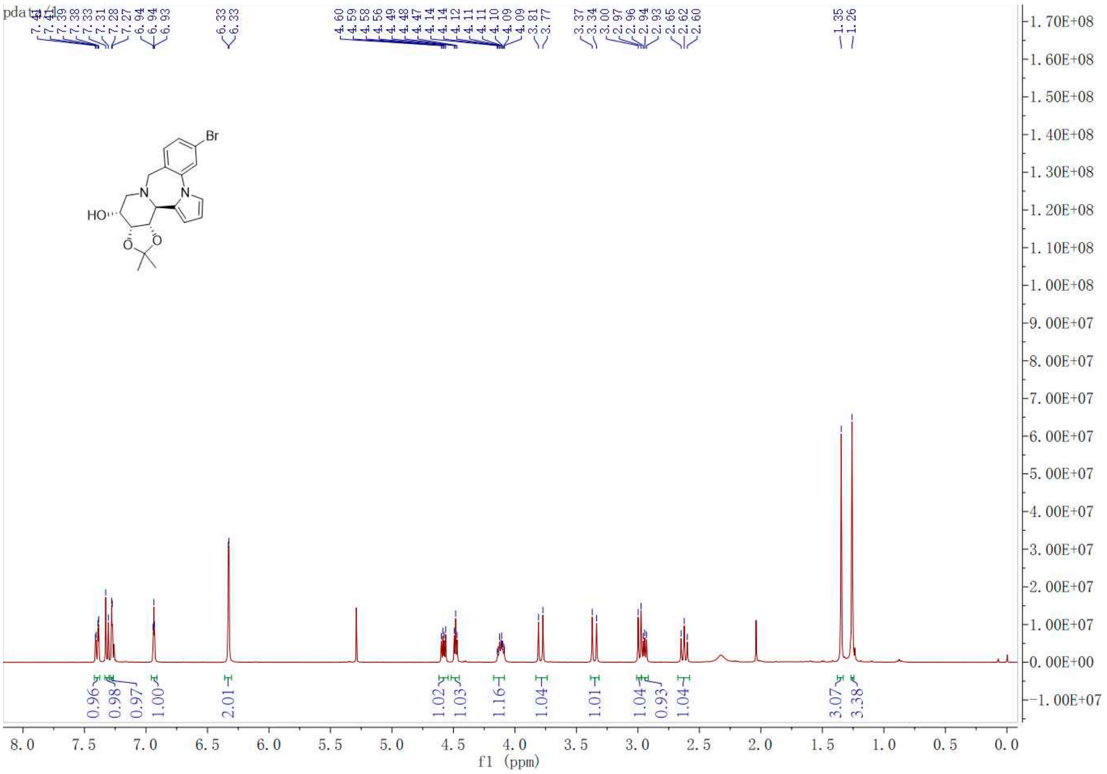

Figure. S51 ^{1}H NMR of compound **5b**

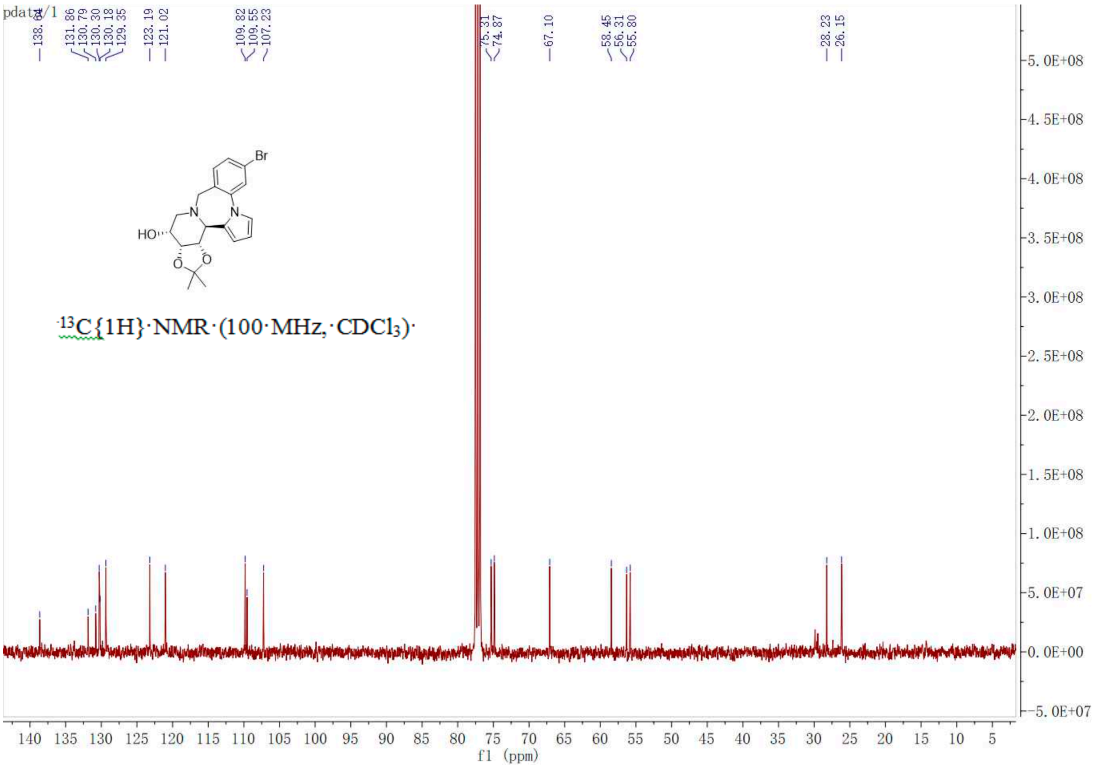

Figure. S52 ^{13}C{^{1}H} NMR of compound **5b**

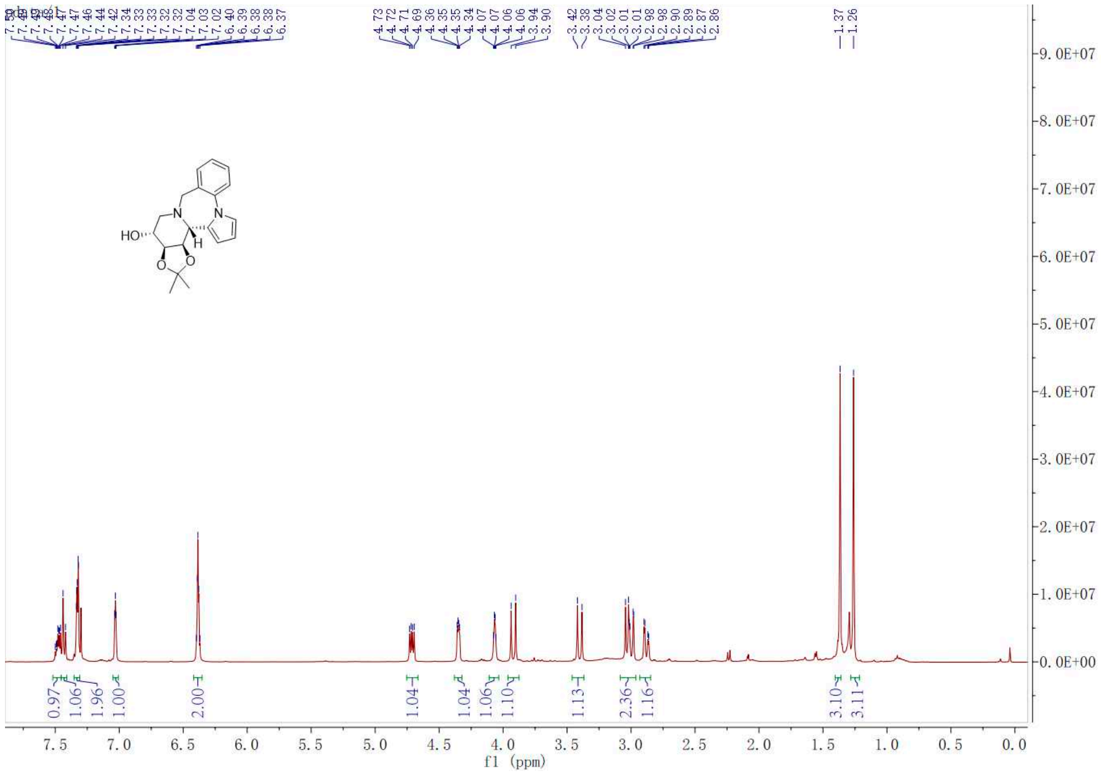

Figure. S53 ^{1}H NMR of compound **6b**

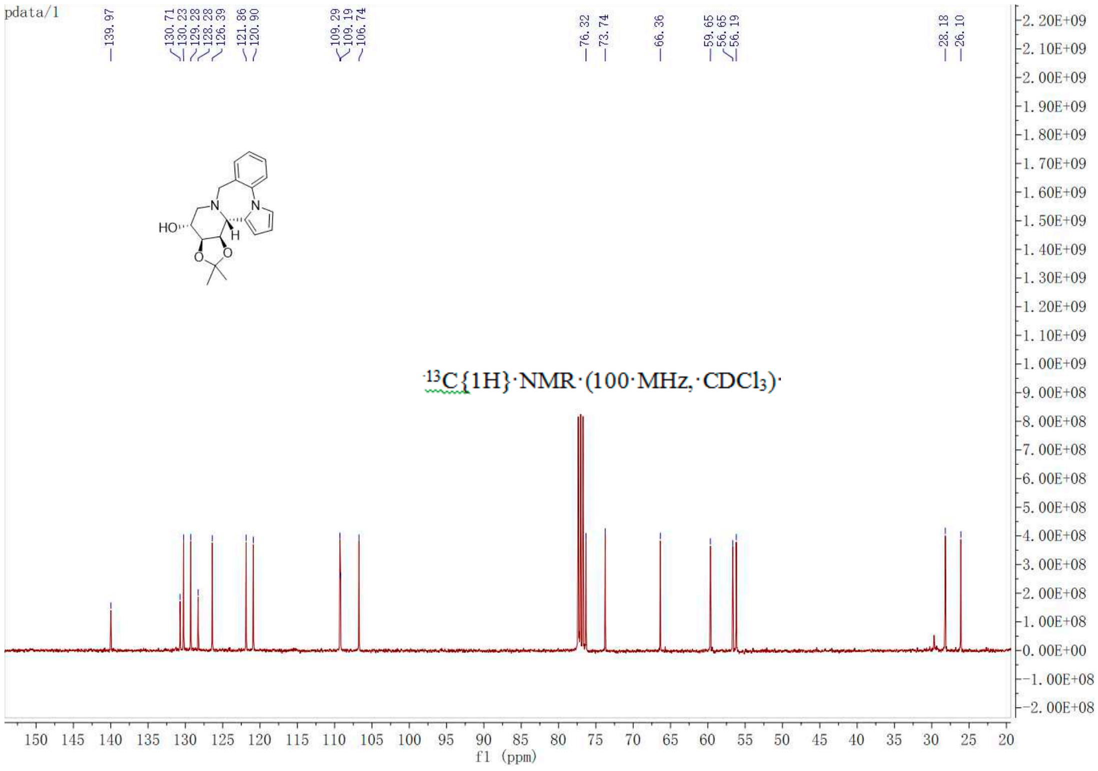

Figure. S54 ^{13}C{^{1}H} NMR of compound **6b**

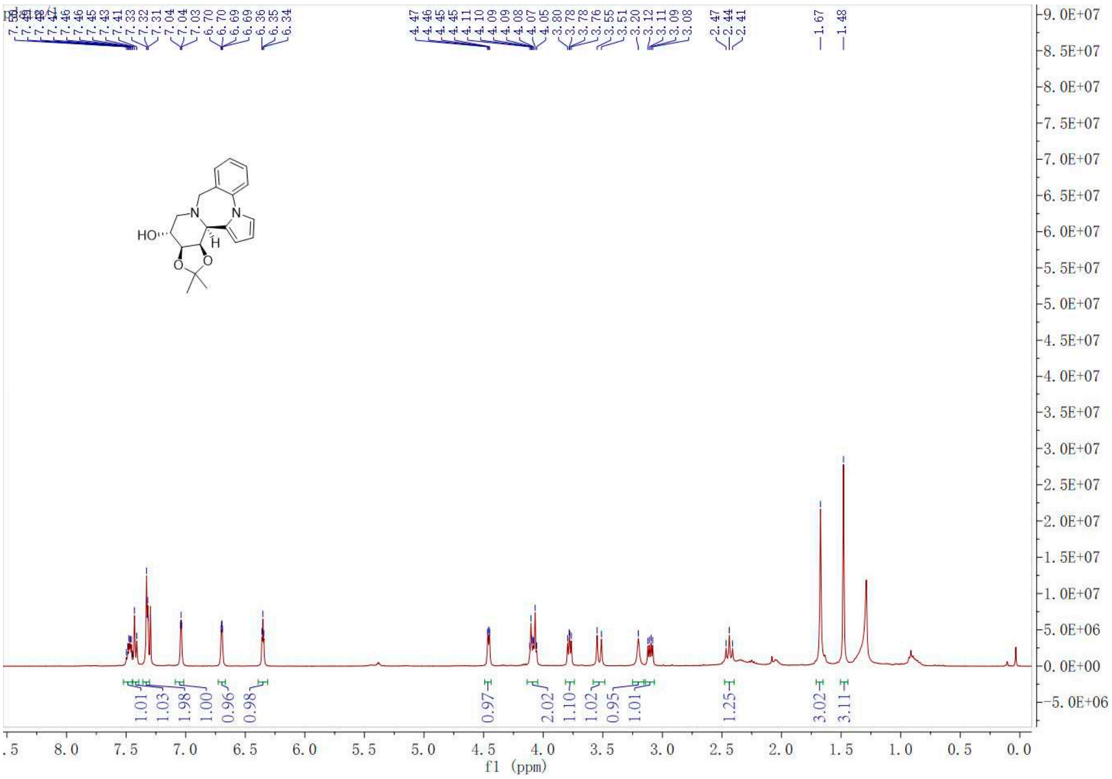

Figure. S55 ^{1}H NMR of compound **6b’**

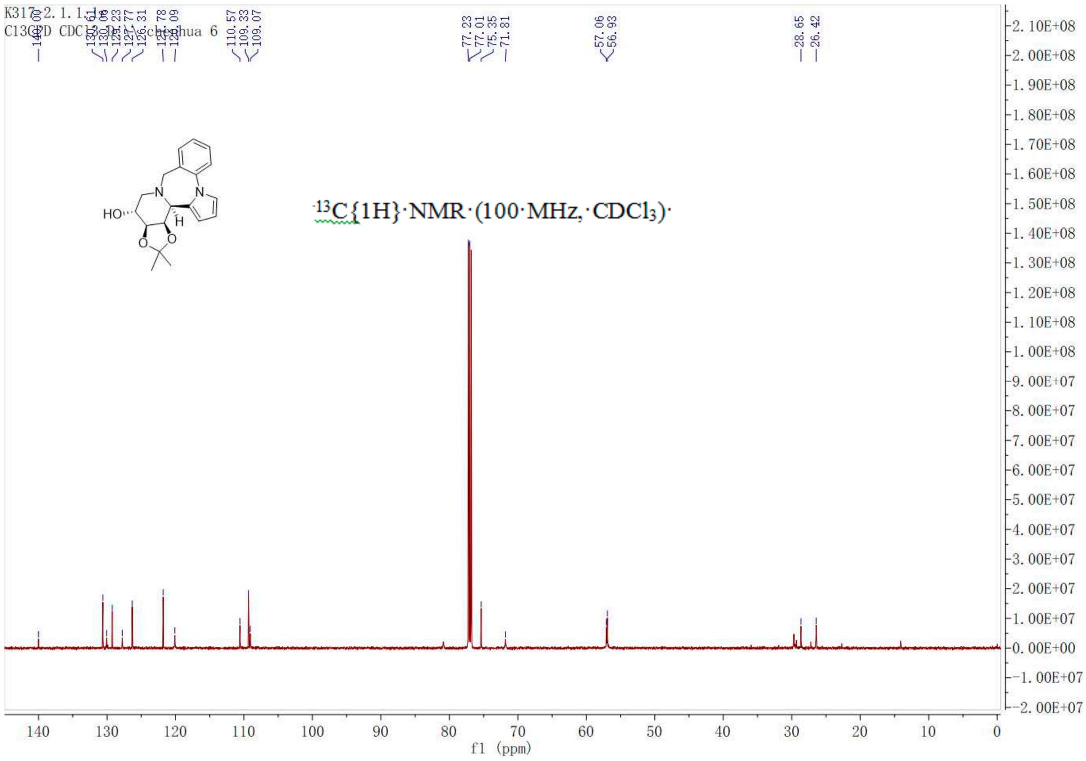

Figure. S56 $^{13}C\{^{1}H\}$ NMR of compound **6b’**

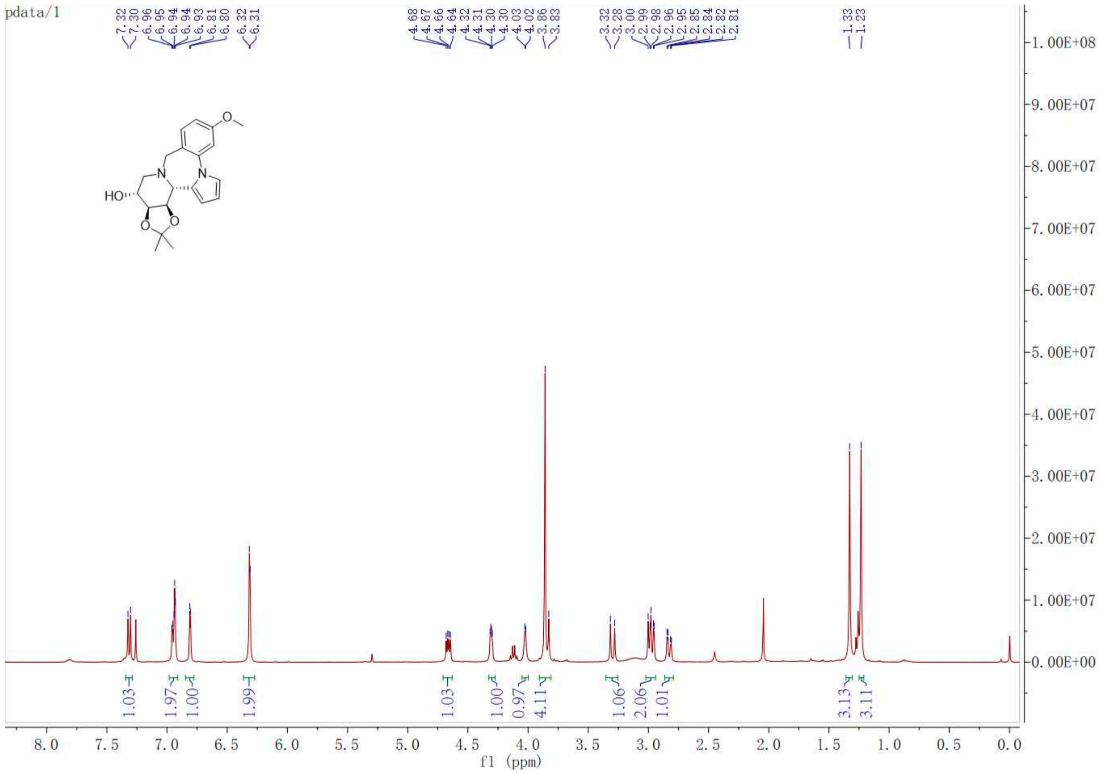

Figure. S57 ^{1}H NMR of compound **7b**

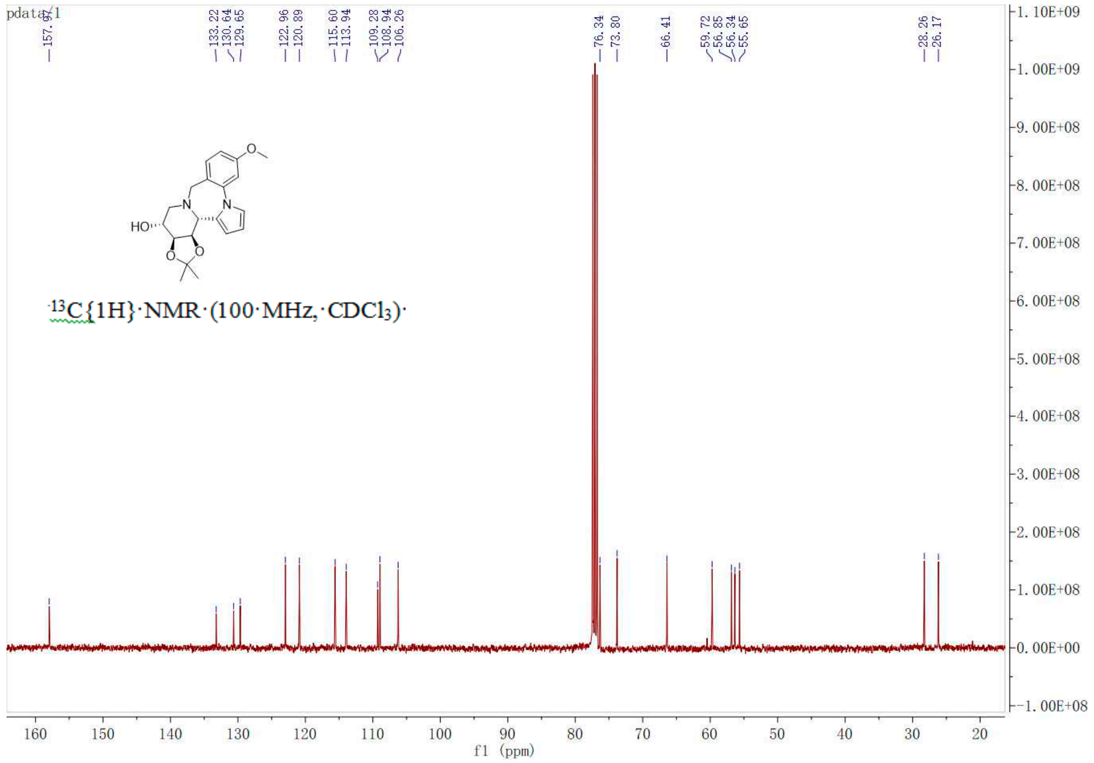

Figure. S58 $^{13}C\{^{1}H\}$ NMR of compound **7b**

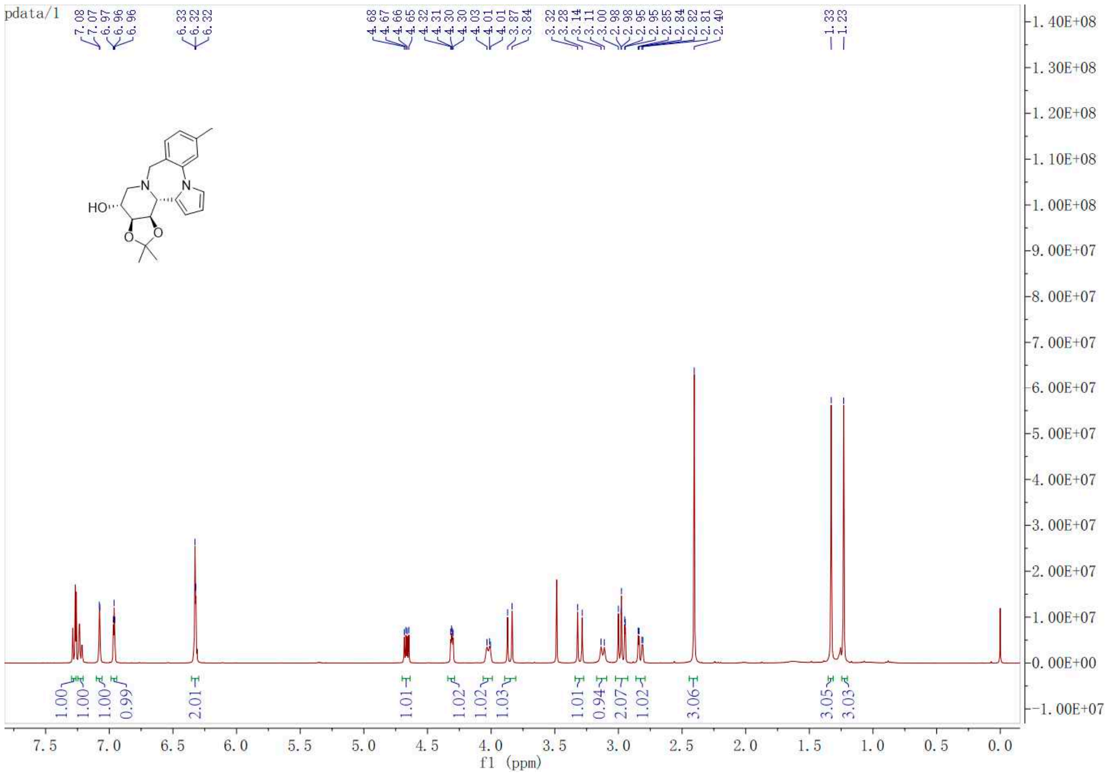

Figure. S59 ^{1}H NMR of compound **8b**

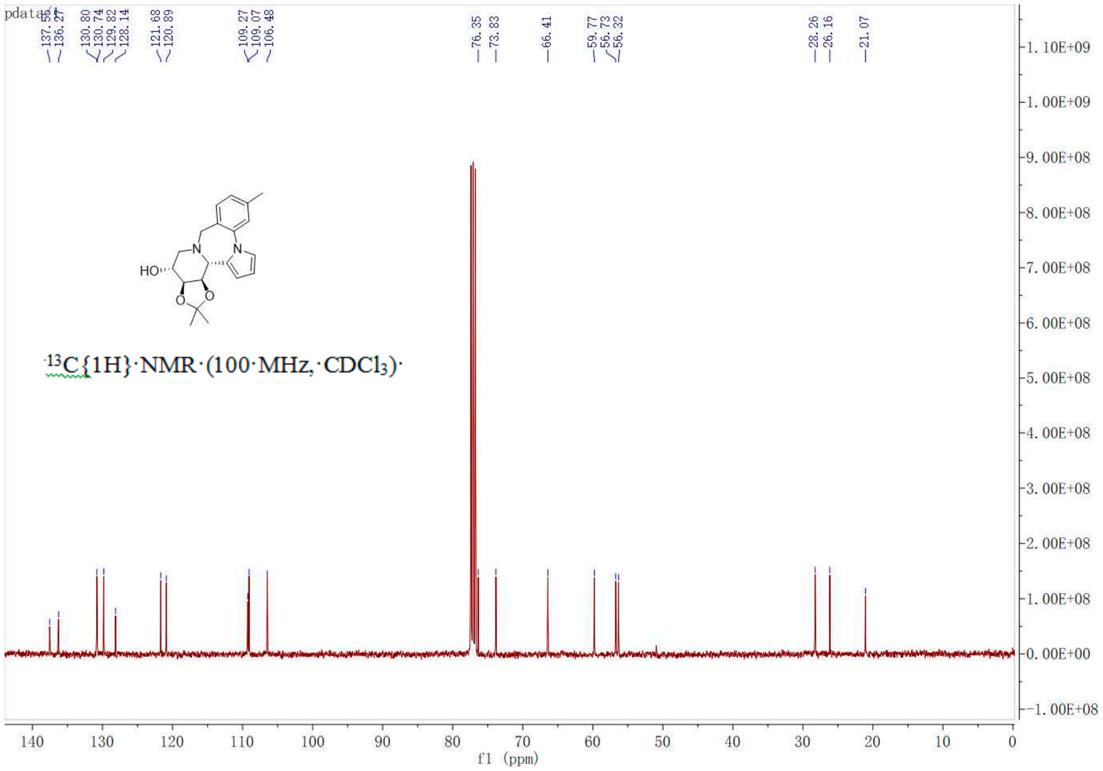

Figure. S60 ^{13}C{^{1}H} NMR of compound **8b**

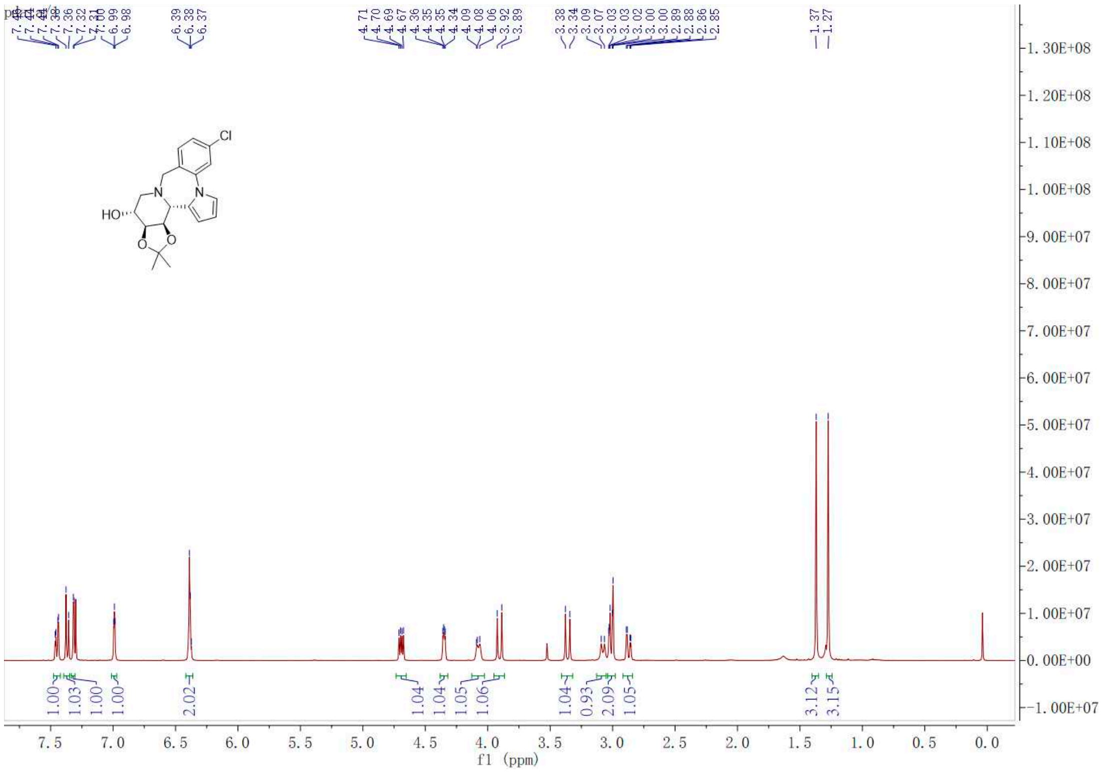

Figure. S61 ^{1}H NMR of compound **9b**

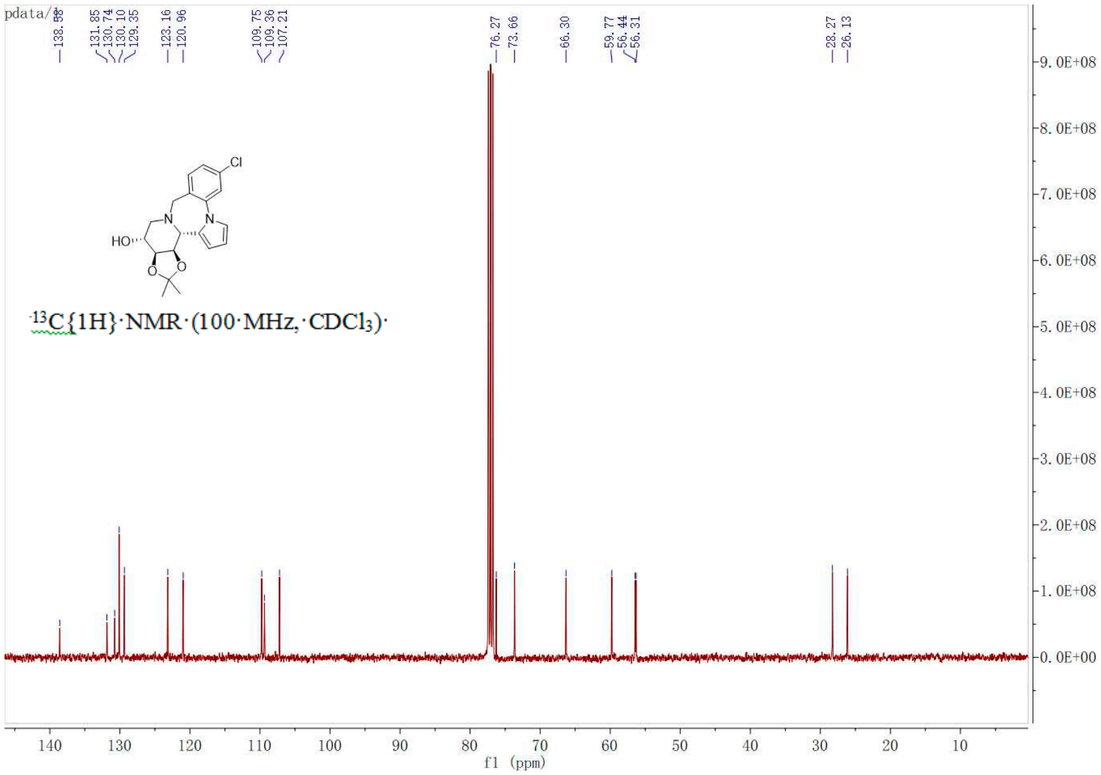

Figure. S62 ^{13}C{^{1}H} NMR of compound **9b**

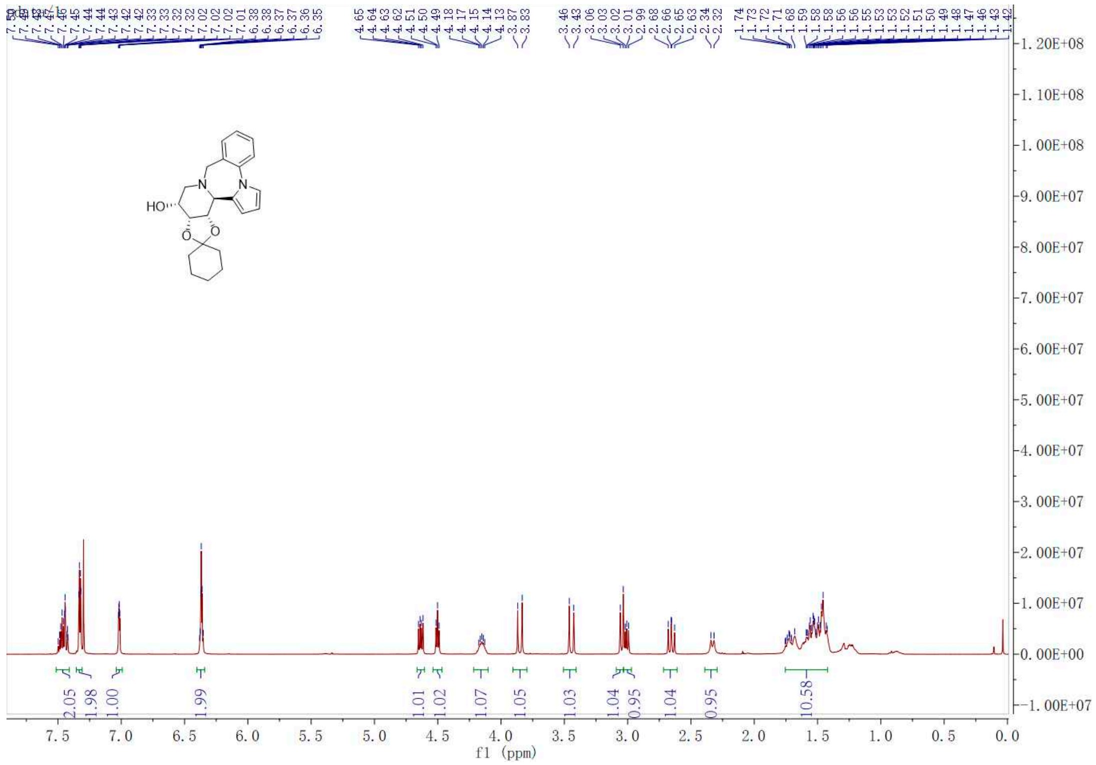

Figure. S63 ^{1}H NMR of compound **10b**

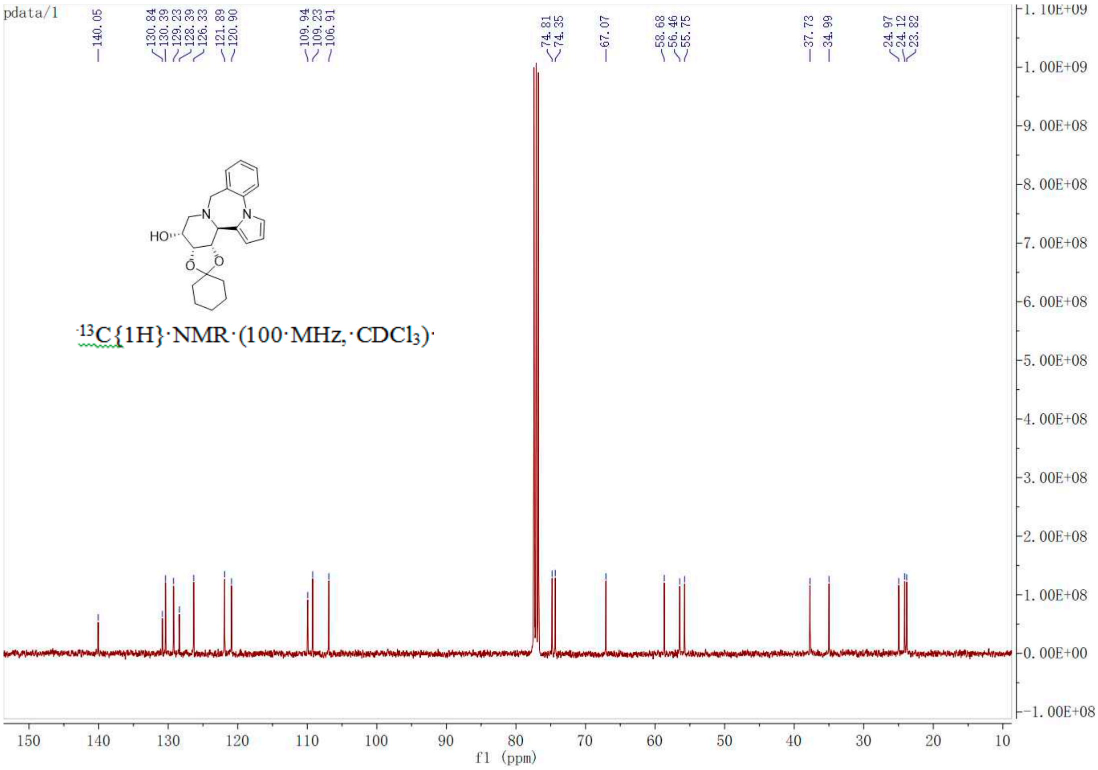

Figure. S64 $^{13}C\{^{1}H\}$ NMR of compound **10b**

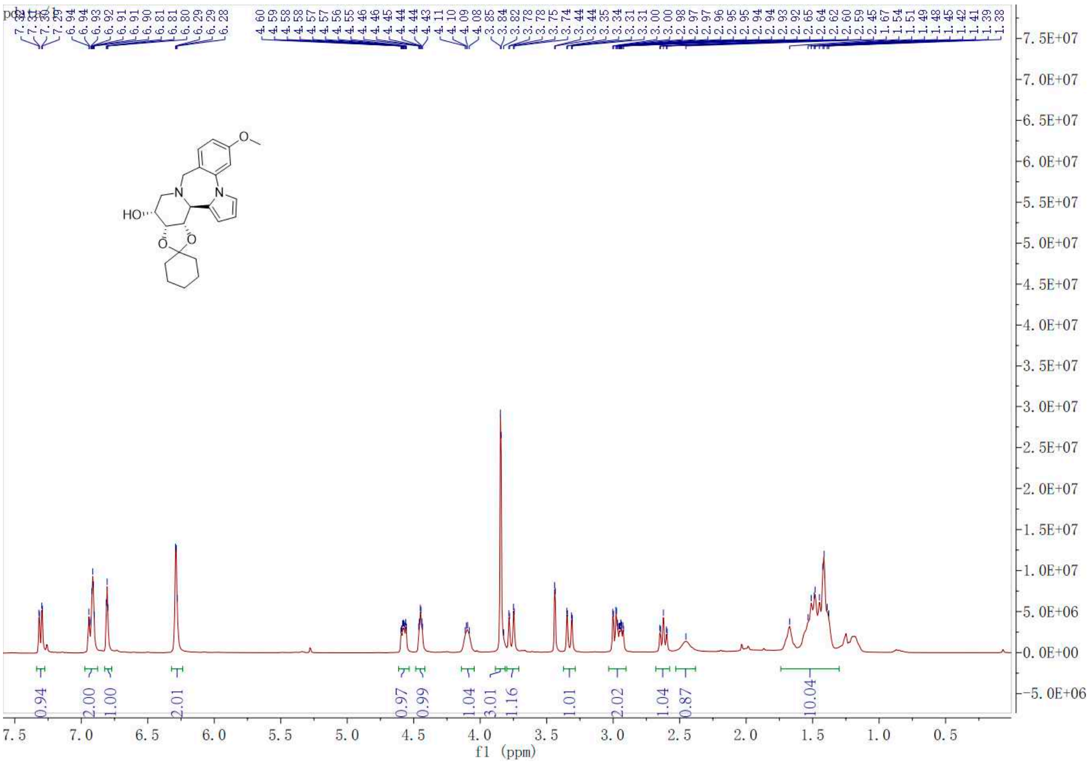

Figure. S65 ^{1}H NMR of compound **11b**

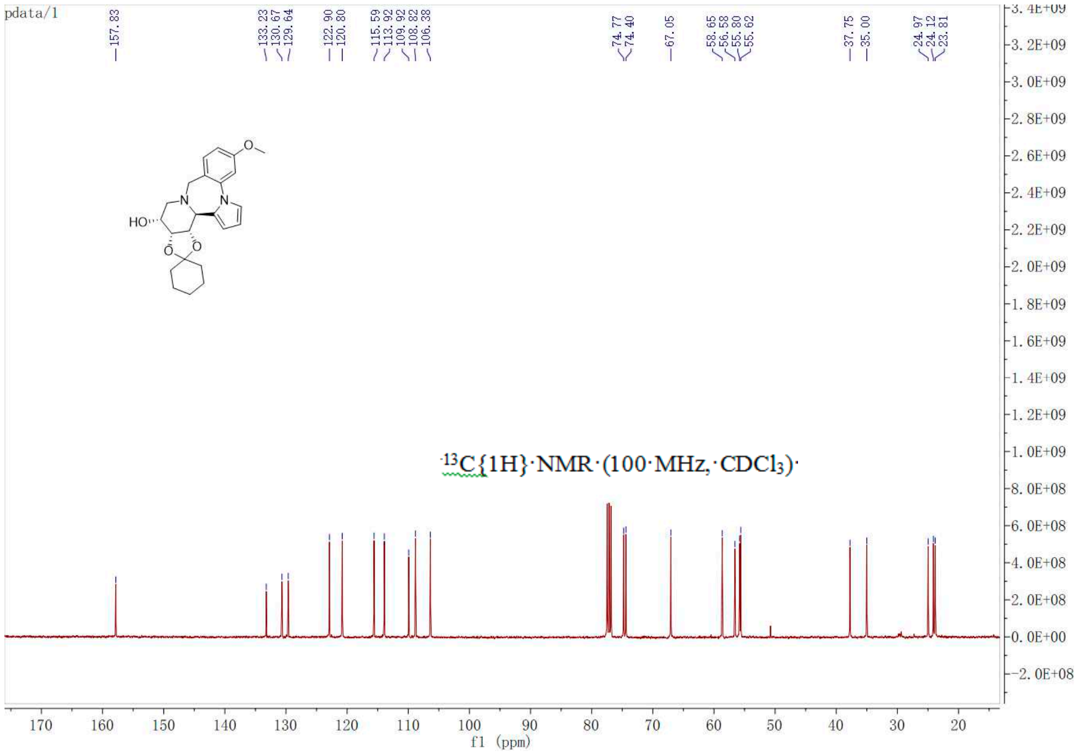

Figure. S66 ^{13}C{^{1}H} NMR of compound **11b**

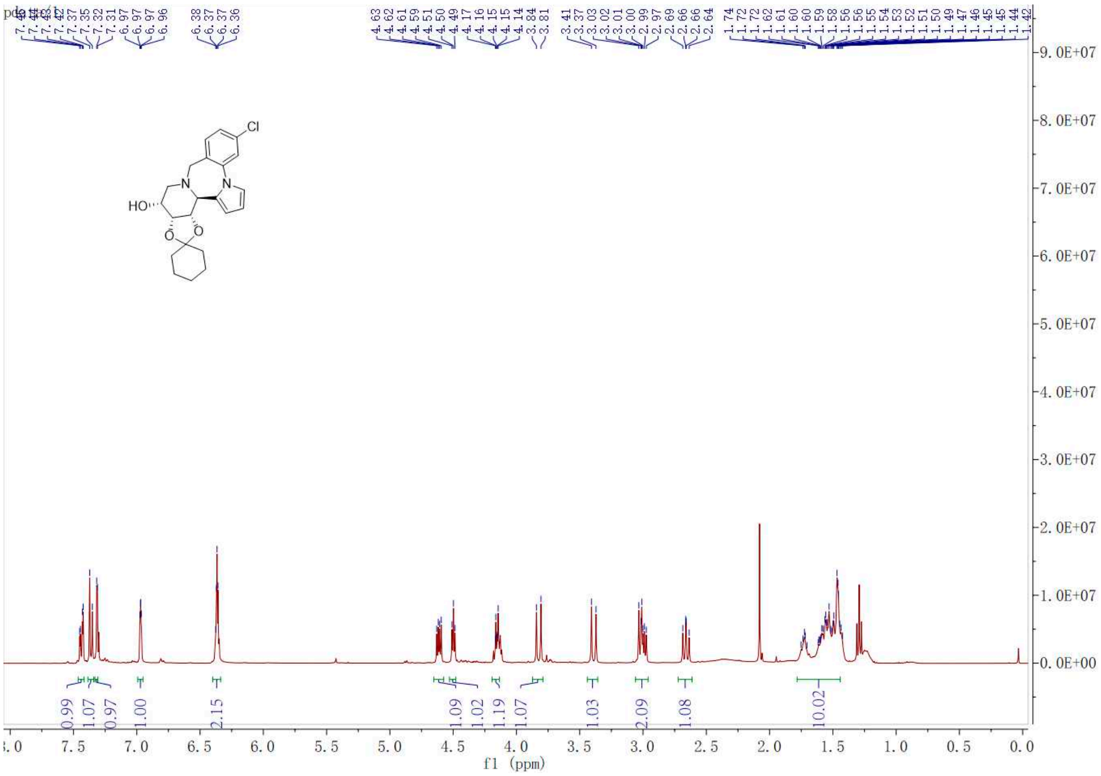

Figure. S67 ^{1}H NMR of compound **12b**

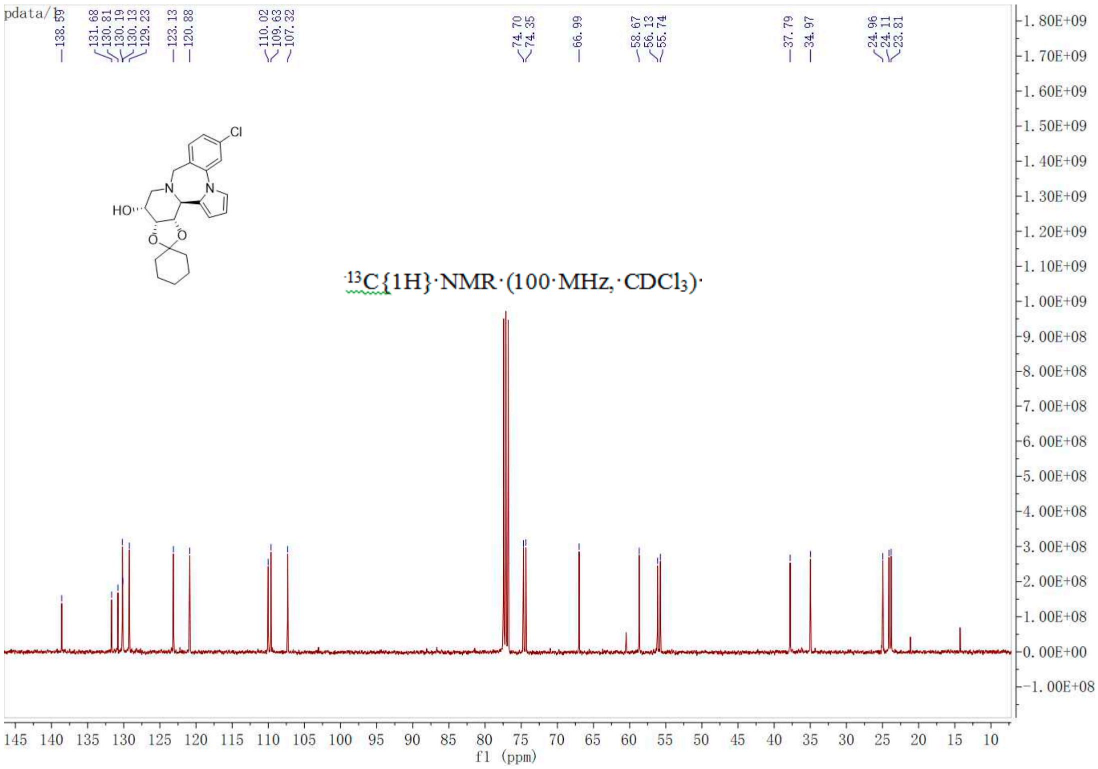

Figure. S68 $^{13}C\{^1H\}$ NMR of compound **12b**

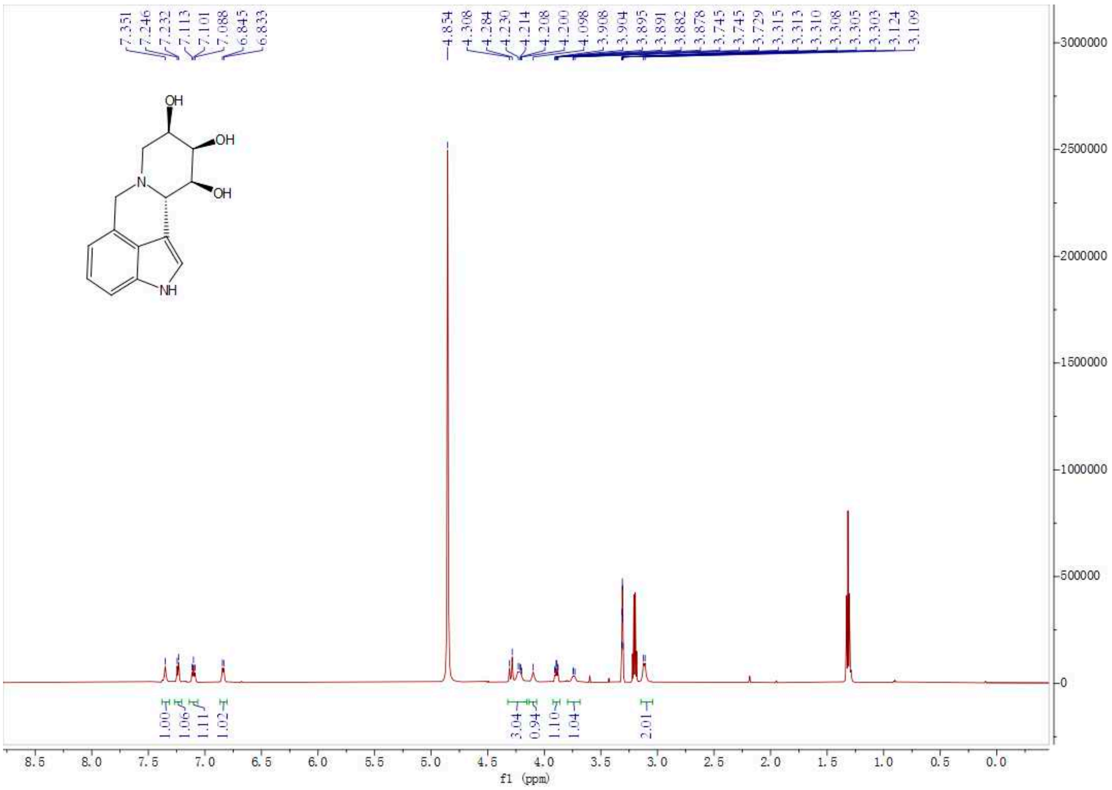

Figure. S69 ^{1}H NMR of compound **1c**

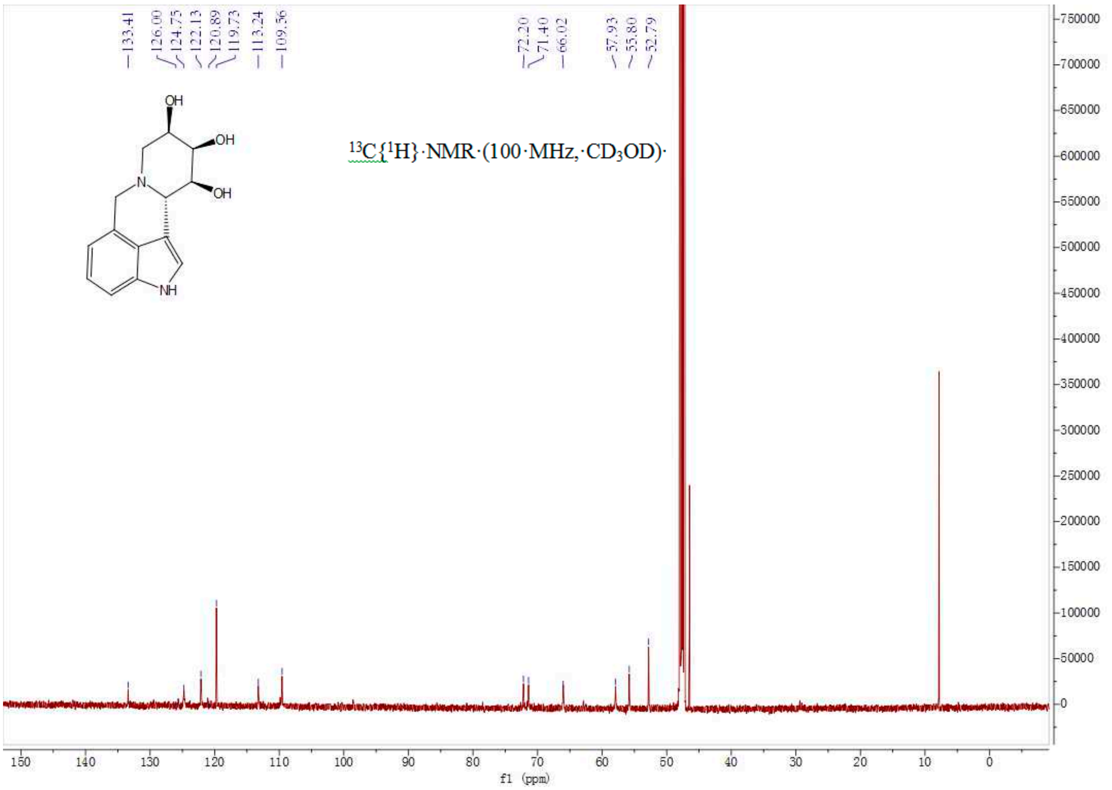

Figure. S70 ^{13}C{^{1}H} NMR of compound **1c**

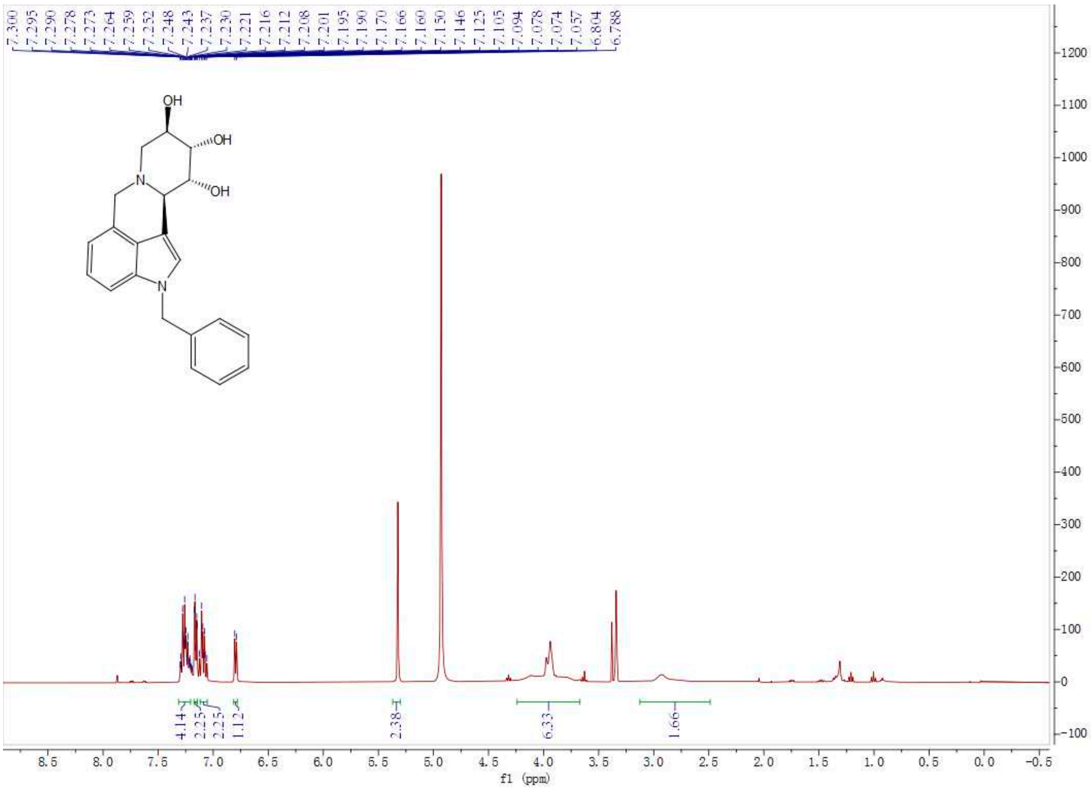

Figure. S71 ^{1}H NMR of compound **2c**

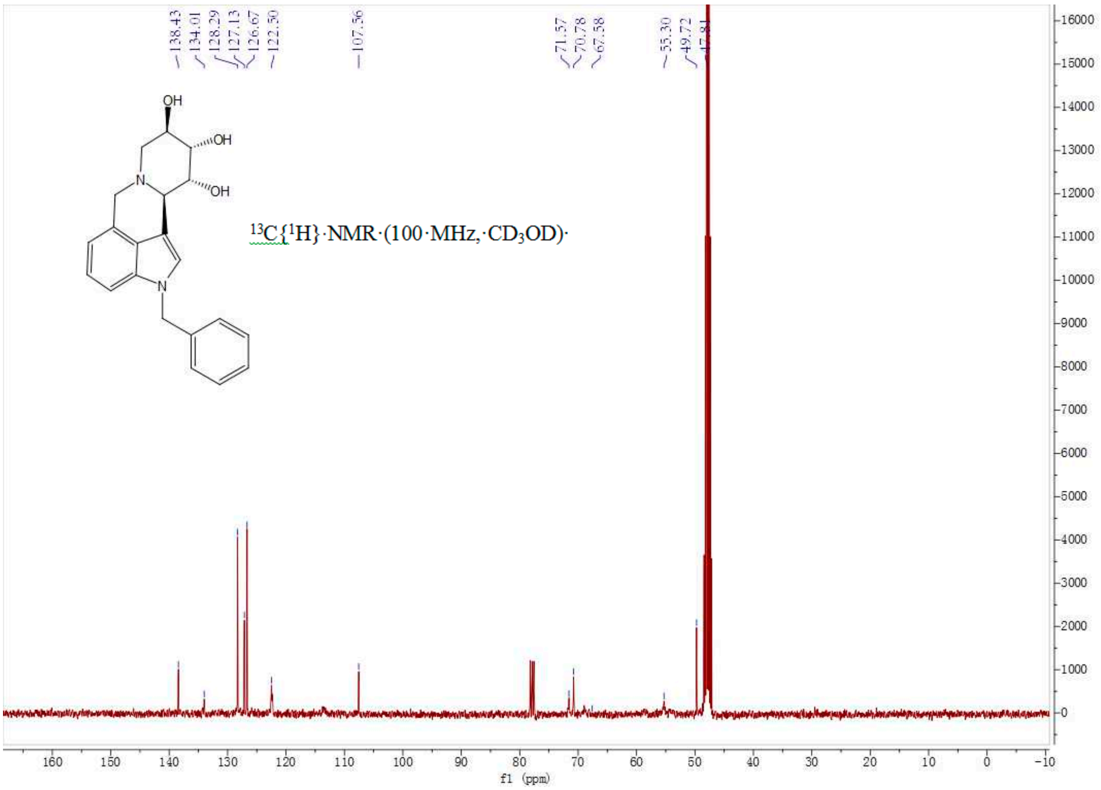

Figure. S72 $^{13}C\{^{1}H\}$ NMR of compound **2c**

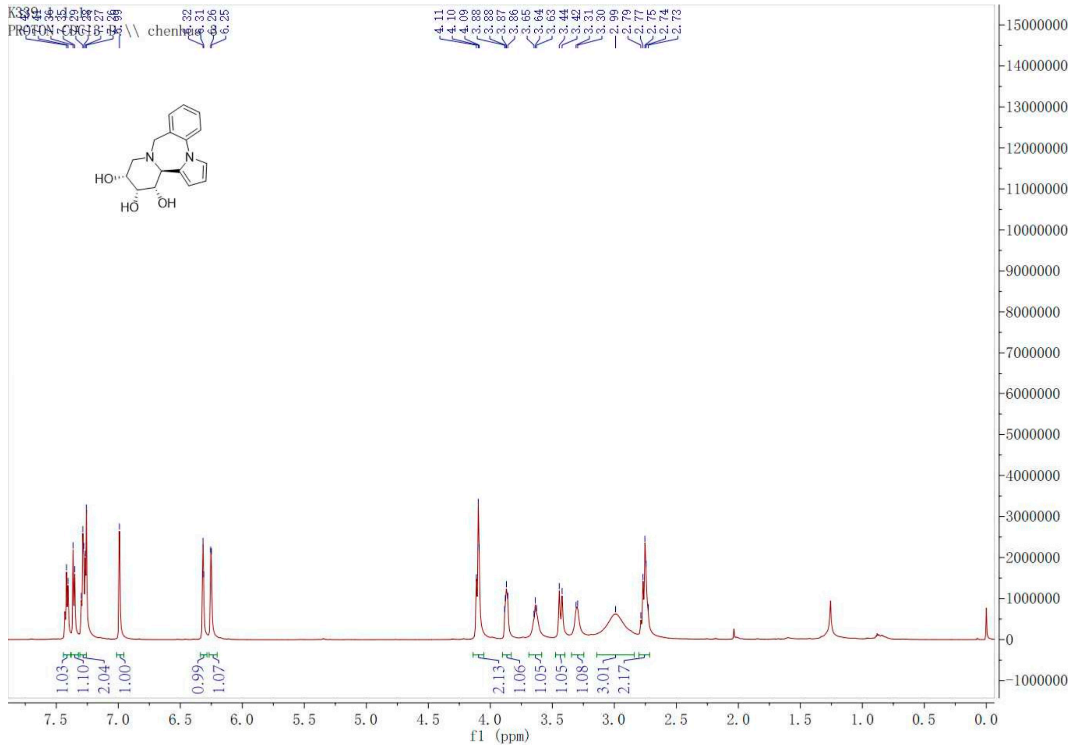

Figure. S73 ^{1}H NMR of compound **3c**

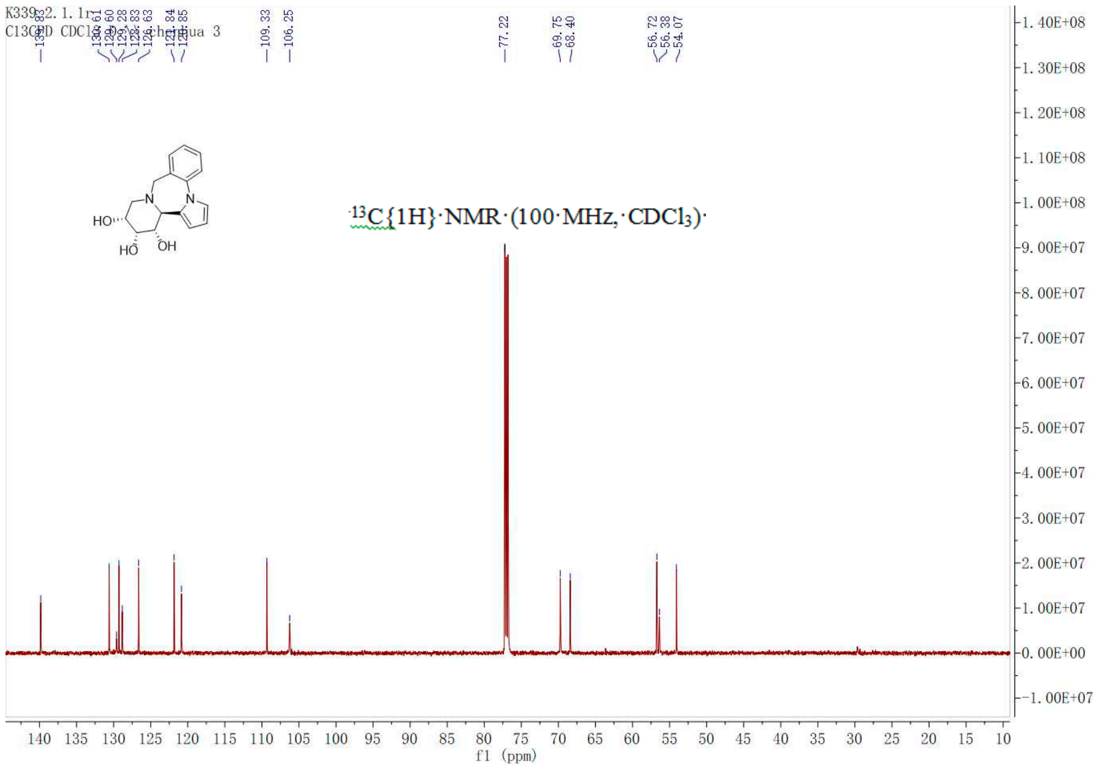

Figure. S74 $^{13}C\{^{1}H\}$ NMR of compound **3c**

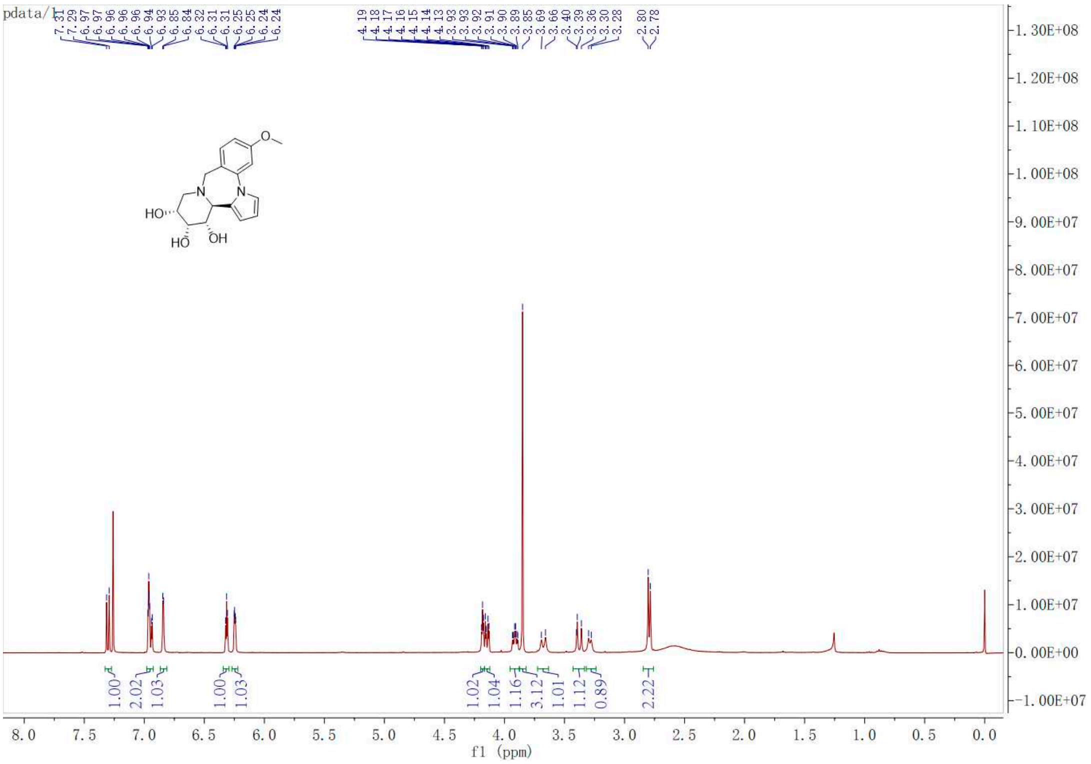

Figure. S75 ^{1}H NMR of compound **4c**

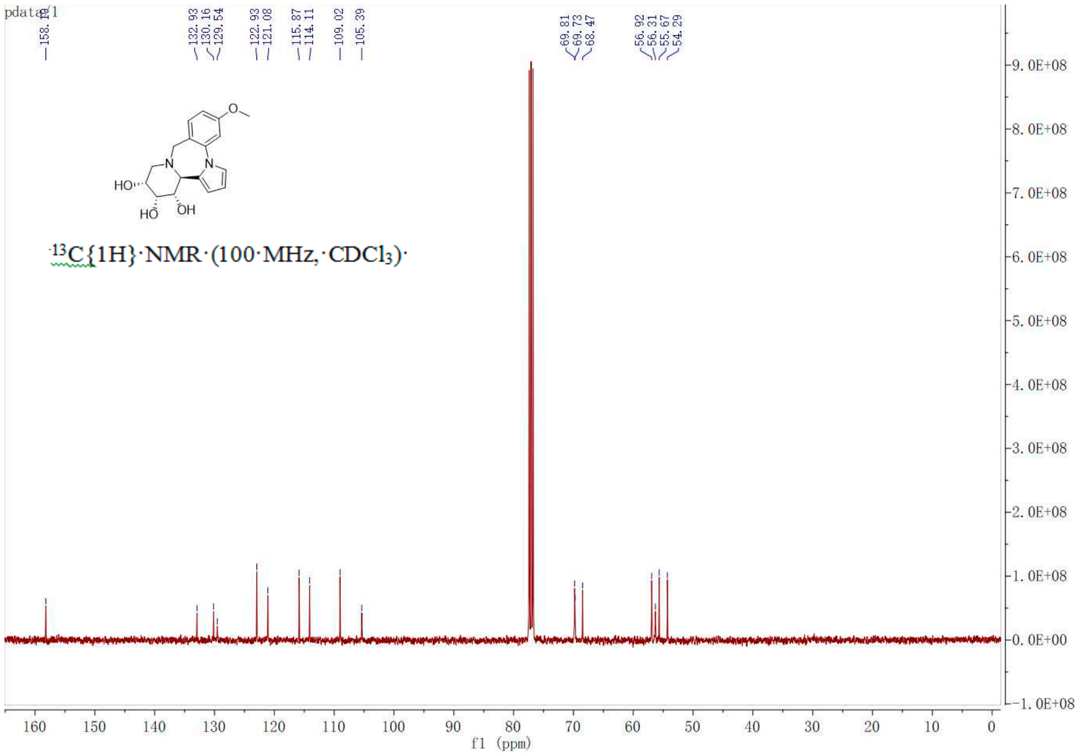

Figure. S76 $^{13}C\{^{1}H\}$ NMR of compound **4c**

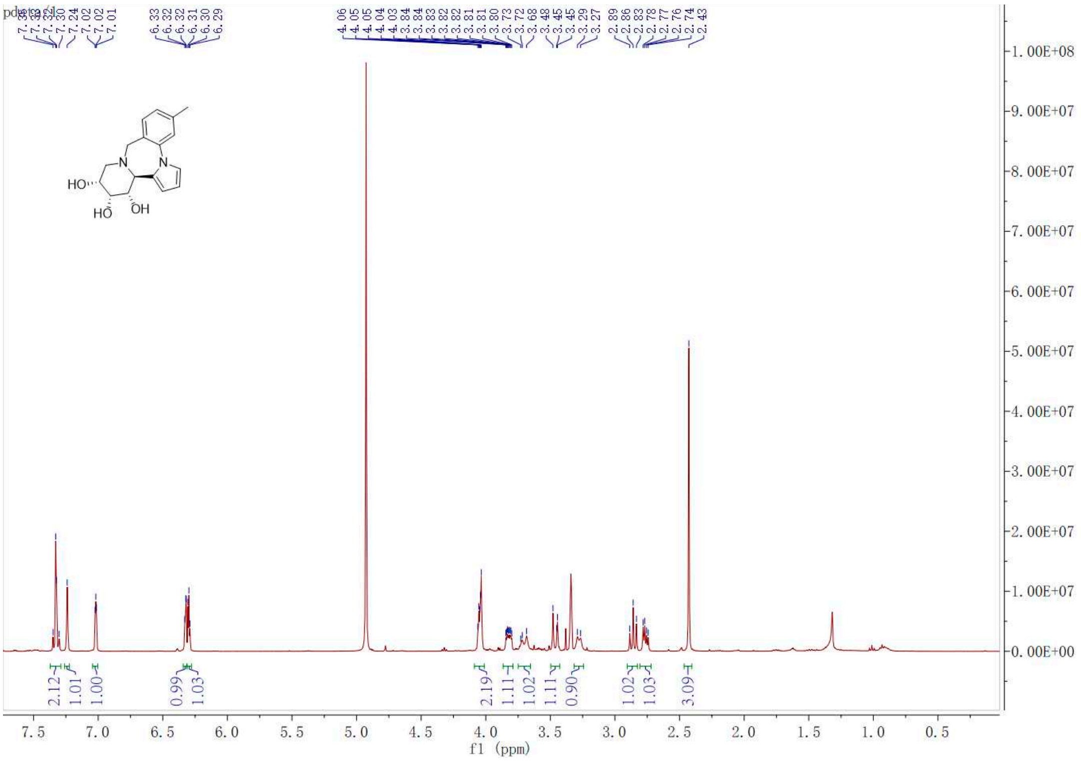

Figure. S77 ^{1}H NMR of compound **5c**

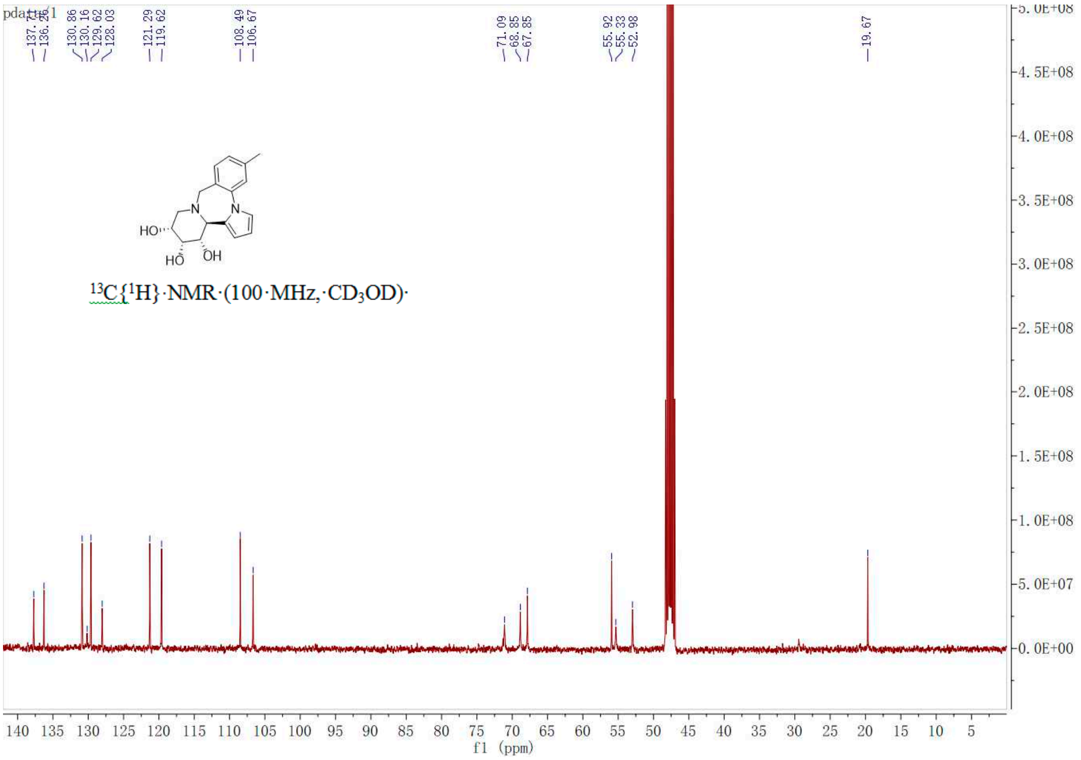

Figure. S78 ^{13}C{^{1}H} NMR of compound **5c**

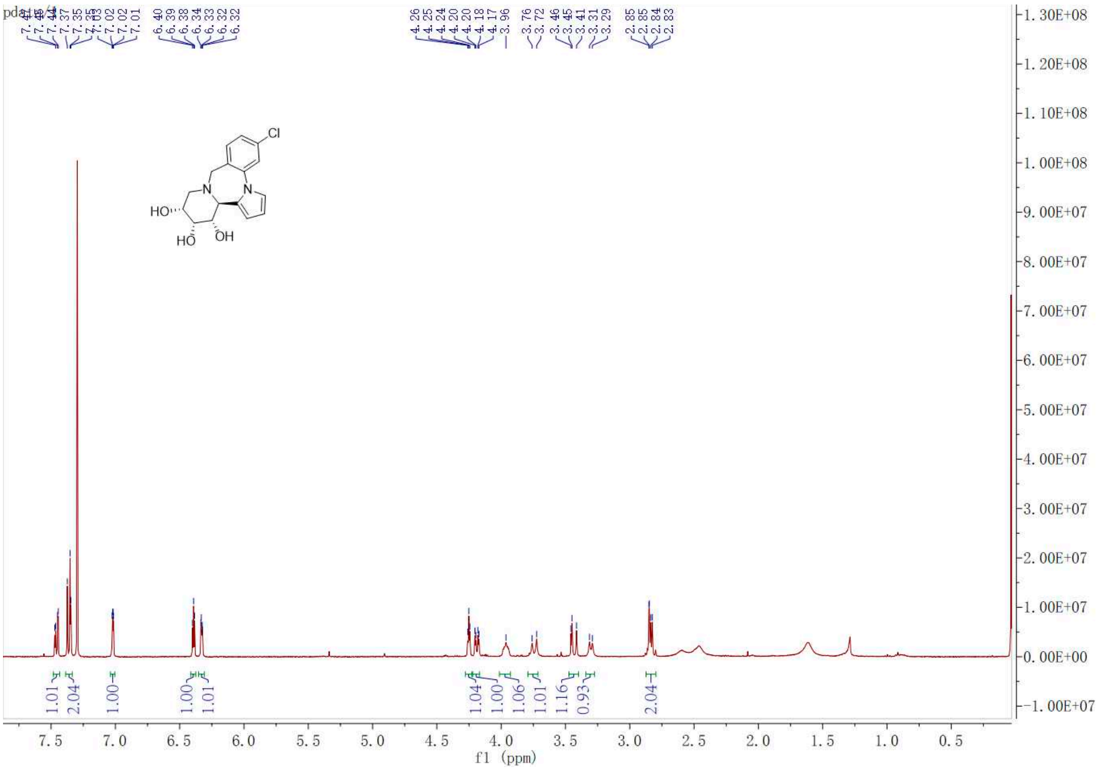

Figure. S79 ^{1}H NMR of compound **6c**

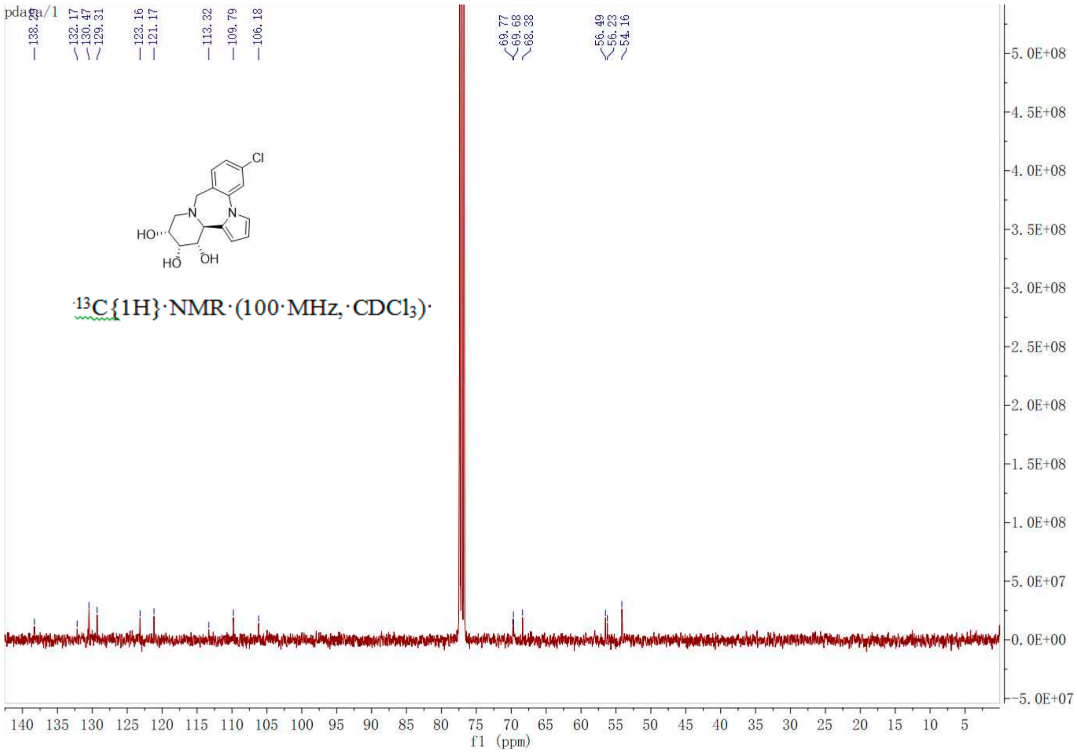

Figure. S80 ^{13}C{^{1}H} NMR of compound **6c**

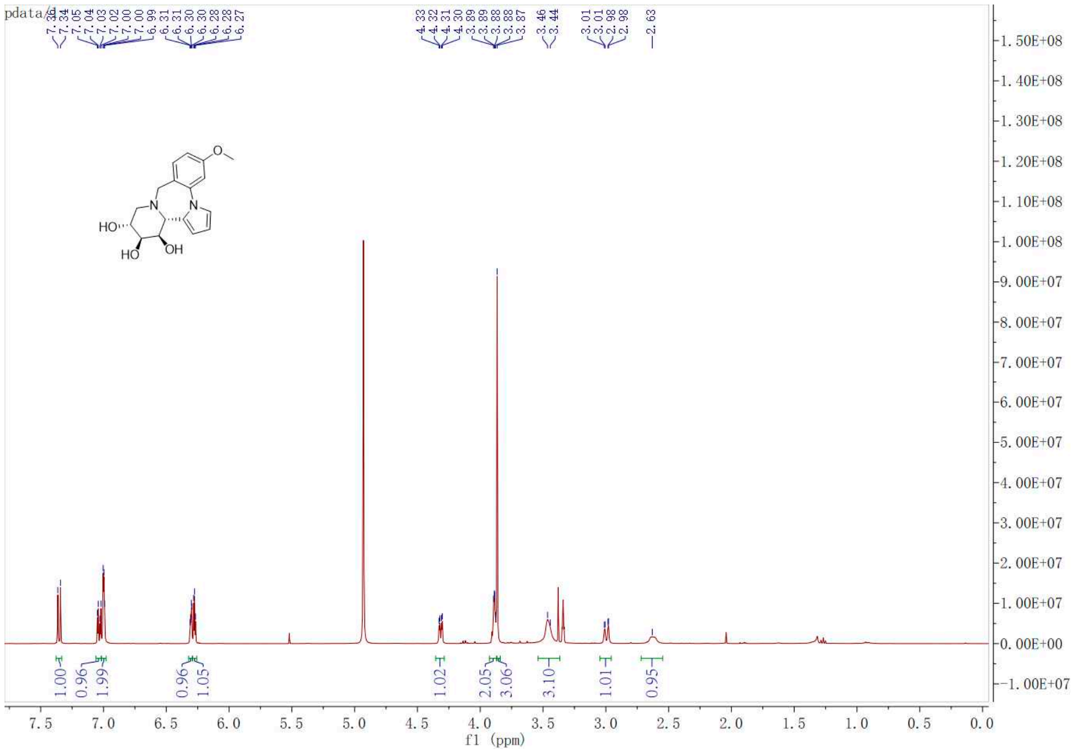

Figure. S81 ^{1}H NMR of compound **7c**

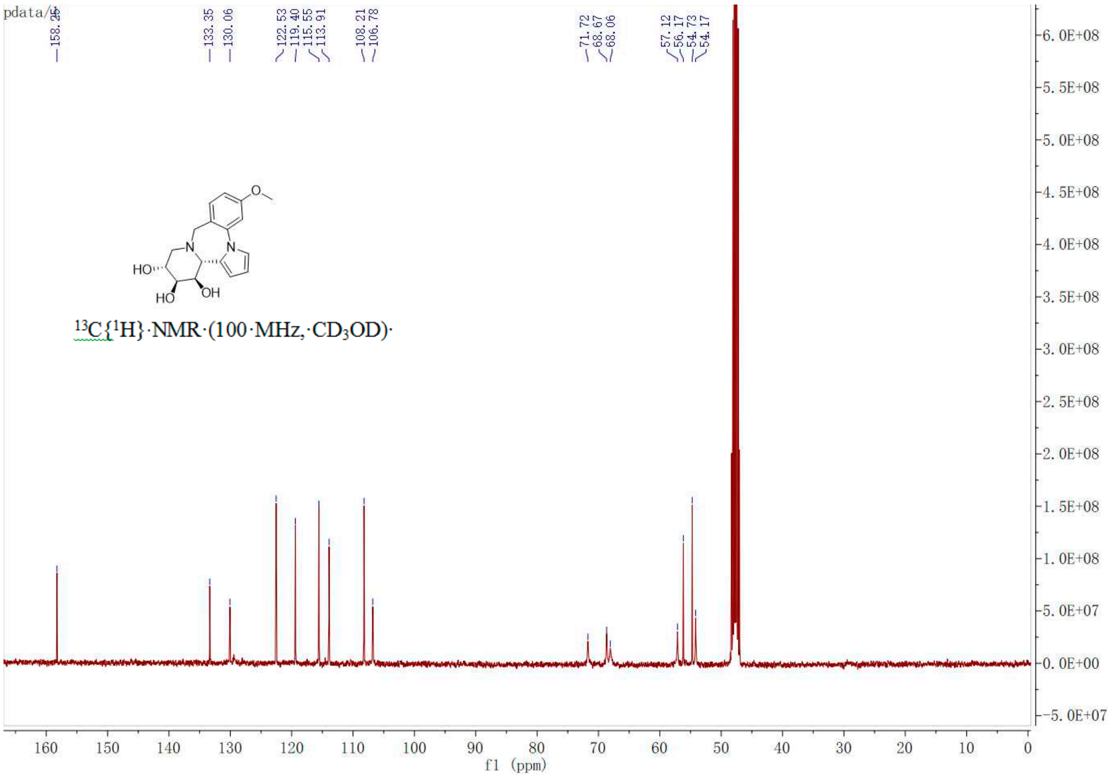

Figure. S82 $^{13}C\{^1H\}$ NMR of compound **7c**

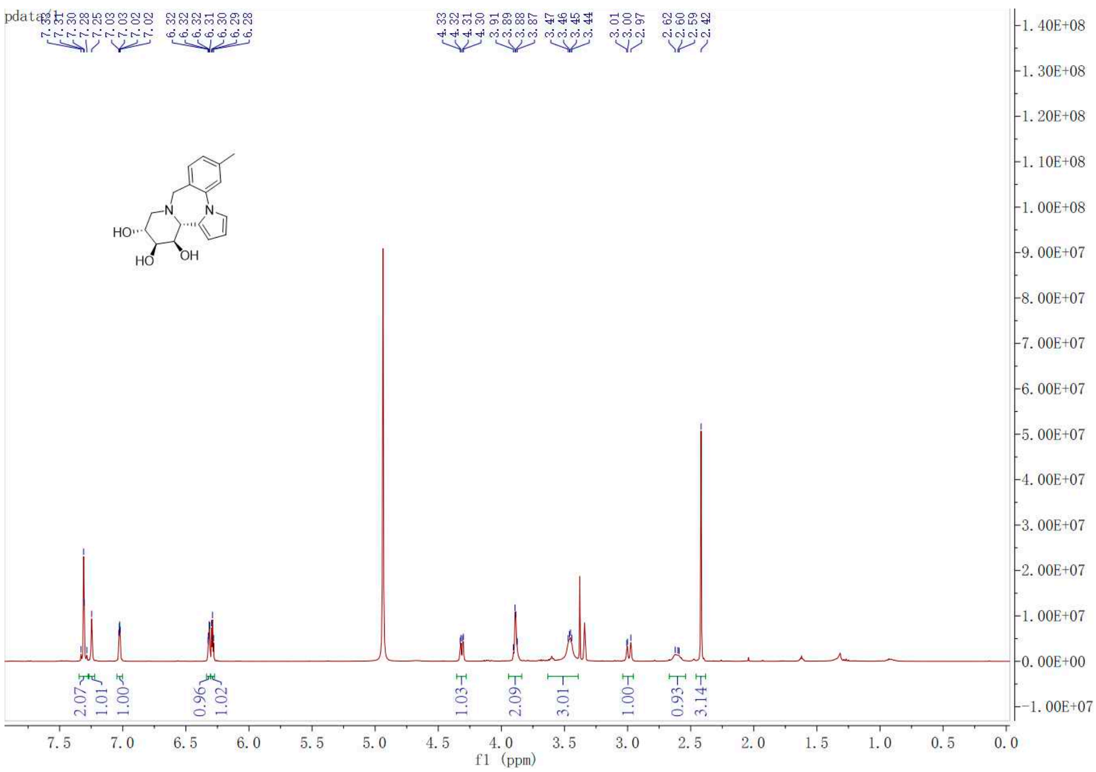

Figure. S83 ^{1}H NMR of compound **8c**

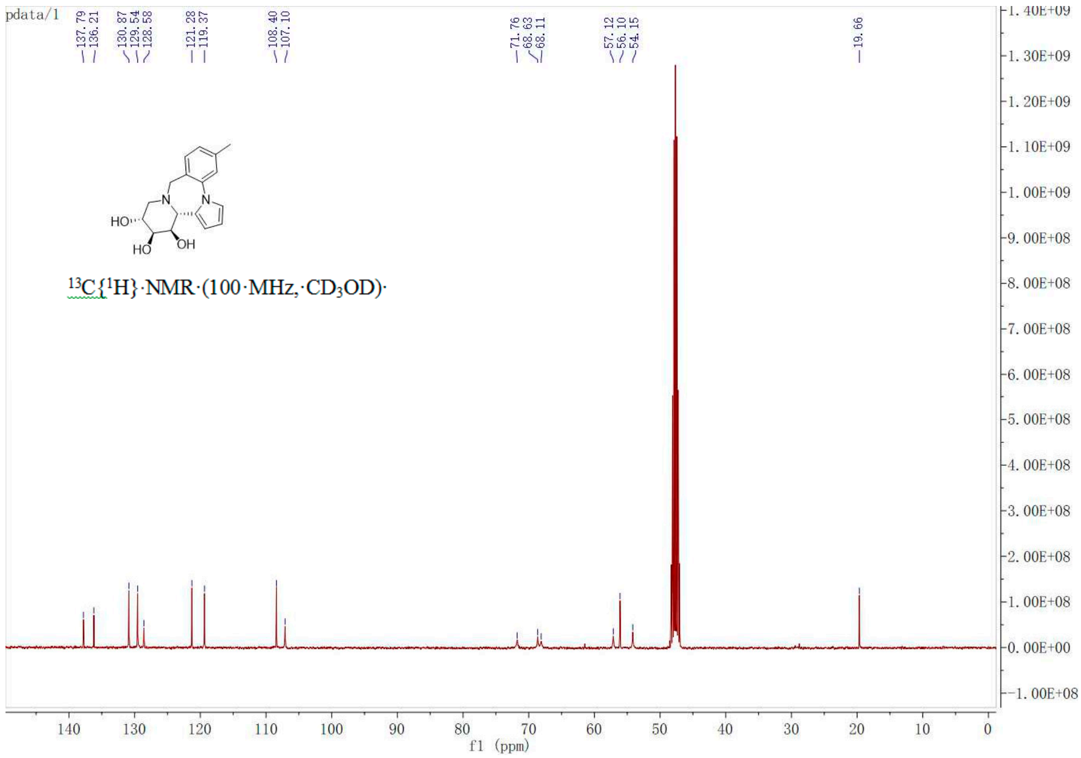

Figure. S84 ^{13}C{^{1}H} NMR of compound **8c**

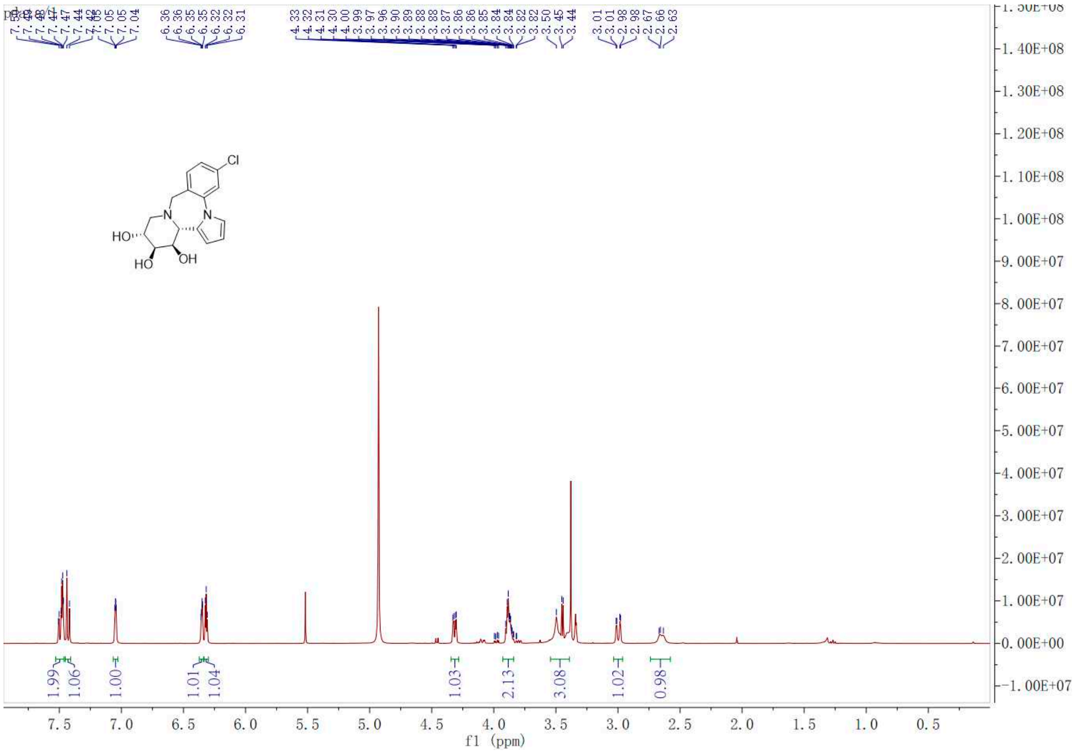

Figure. S85 ^{1}H NMR of compound **9c**

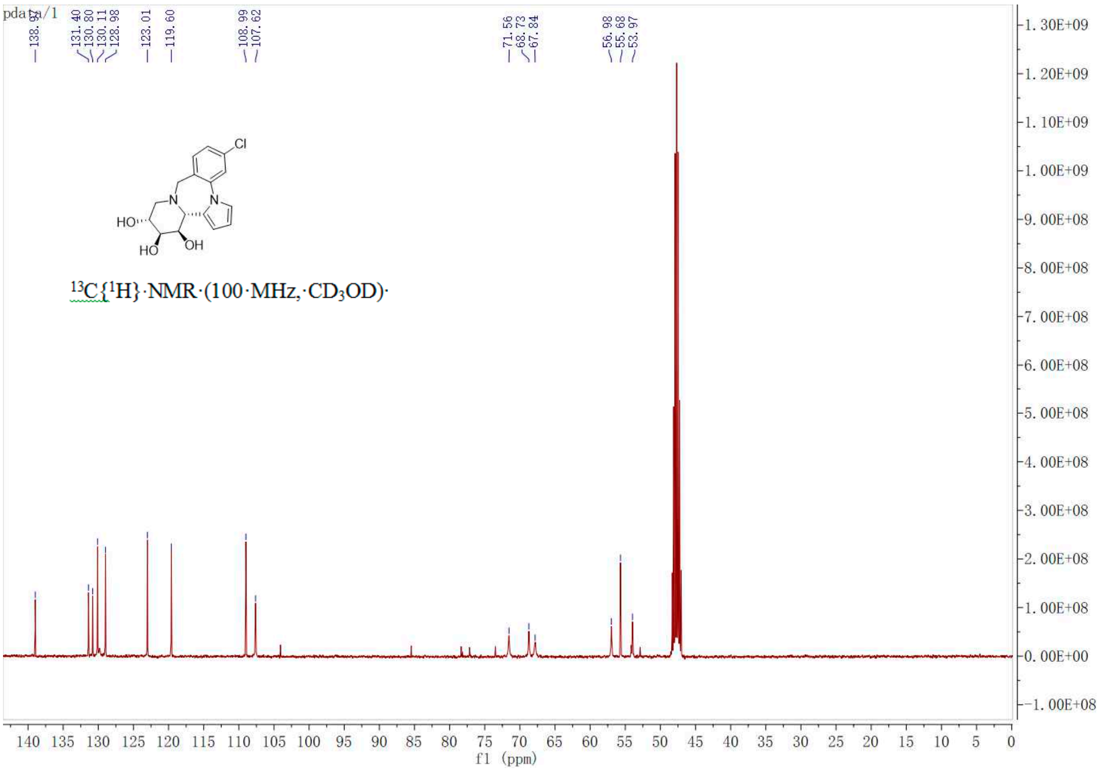

Figure. S86 $^{13}C\{^{1}H\}$ NMR of compound **9c**

2D-NMR spectra of 1b:

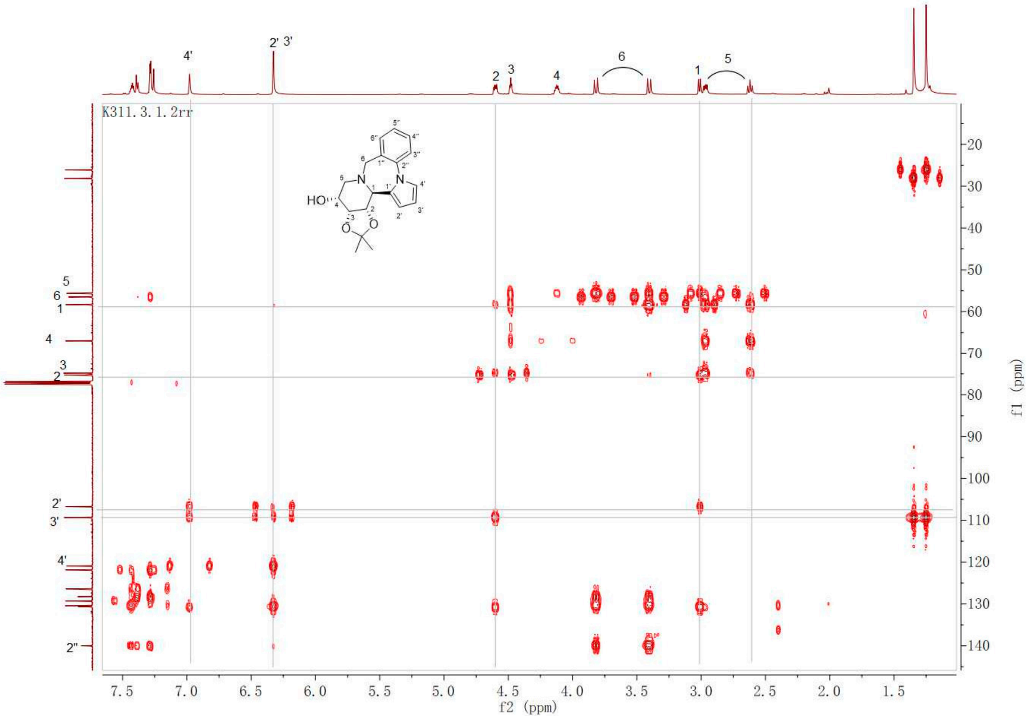

Figure. S87 ^{1}H-^{13}C HMBC NMR of compound **1b**

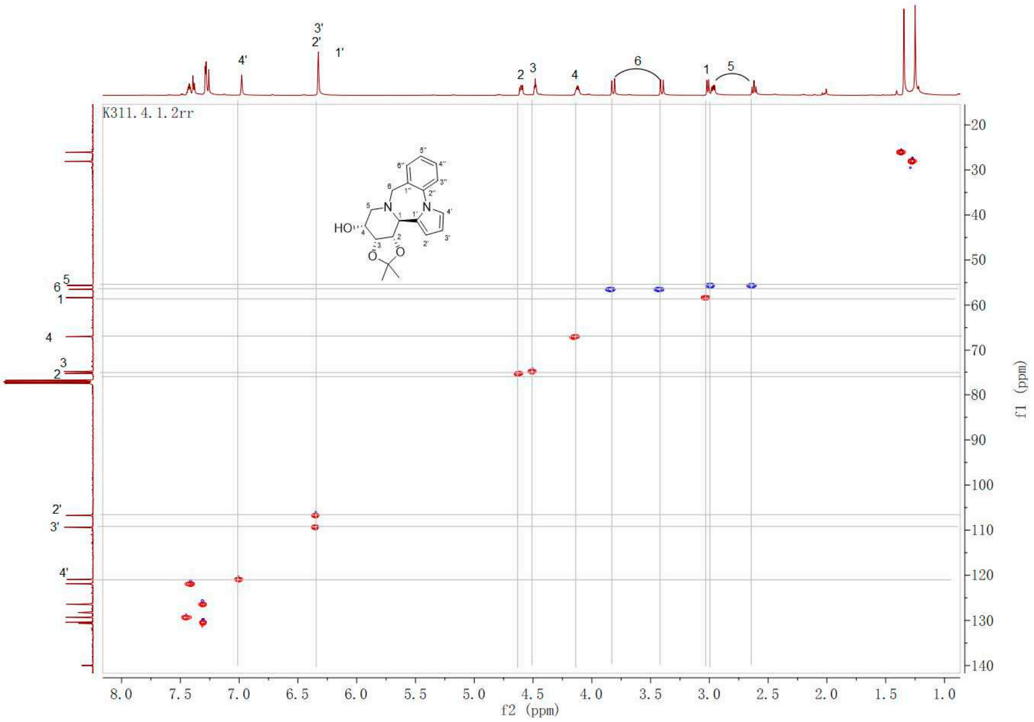

Figure. S88 ^{1}H-^{13}C HSQC NMR of compound **1b**

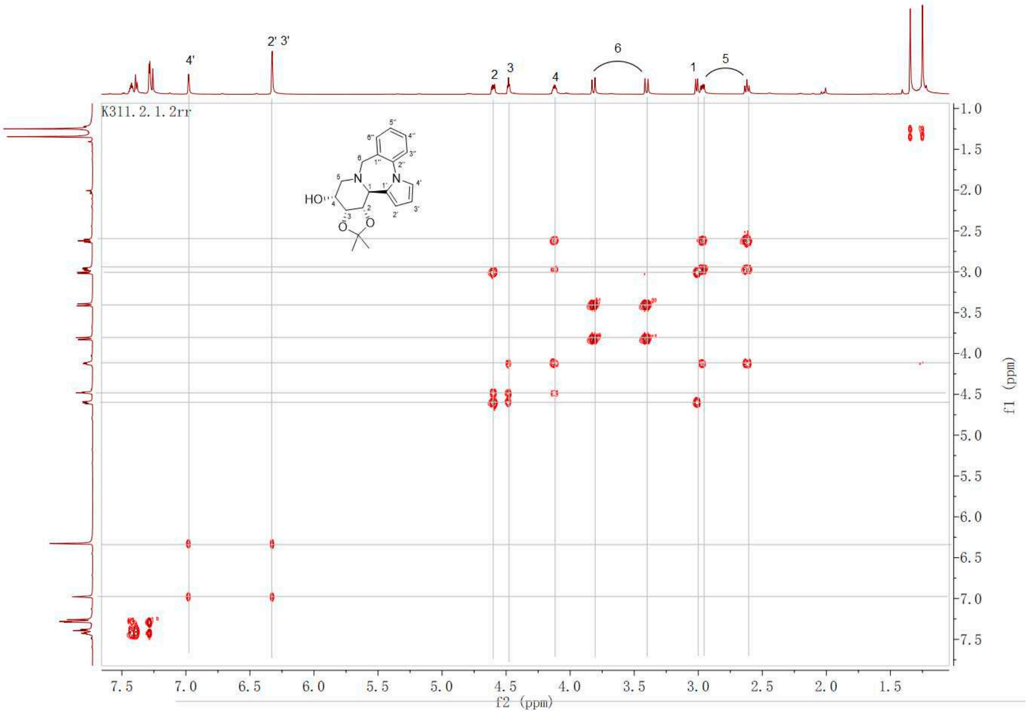

Figure S89 ^{1}H-^{1}H COSY NMR of compound **1b**

3. Crystal Information:

To determine the absolute configureuration of **14a**: Firstly, **14a** was recrystallized from dichloromethane/methabol. The solvents were slowly evaporated directly, and the single crystal was obtained after three days. The CCDC number is 2391570.

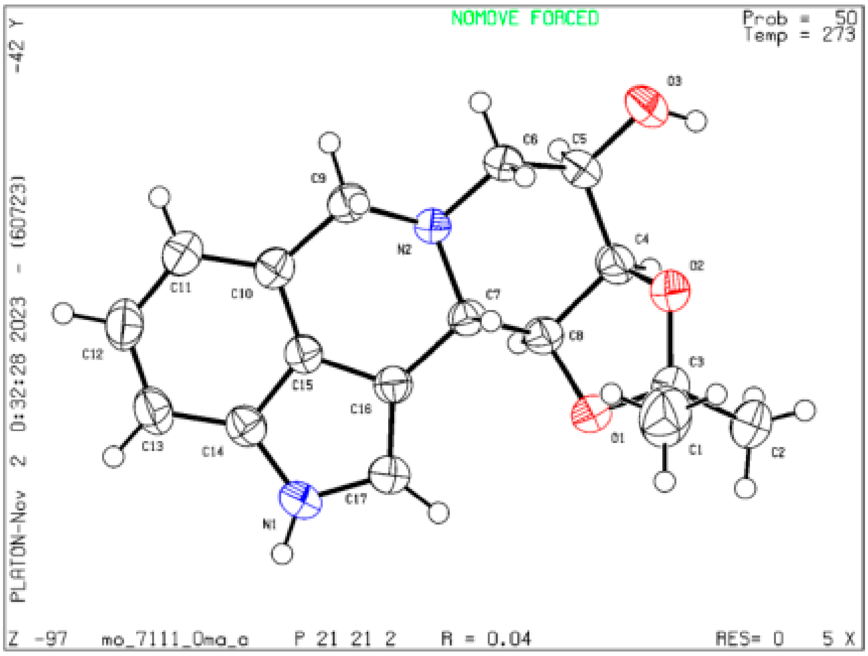

Bond precision: C-C = 0.0033 A Wavelength=0.71073

Cell:	a=9.3089(4)	b=26.3277(11)	c=6.1558(3)
	alpha=90	beta=90	gamma=90
Temperature:	273 K		

	Calculated	Reported
Volume	1508.68(12)	1508.68(12)
Space group	P 21 21 2	P 21 21 2
Hall group	P 2 2ab	P 2 2ab
Moiety formula	C17 H20 N2 O3	C17 H20 N2 O3
Sum formula	C17 H20 N2 O3	C17 H20 N2 O3
Mr	300.35	300.35
Dx,g cm-3	1.322	1.322
Z	4	4
Mu (mm-1)	0.091	0.091
F000	640.0	640.0
F000'	640.29	
h,k,lmax	12,34,7	12,34,7
Nref	3449[2018]	3445
Tmin,Tmax		0.669,0.746
Tmin'		

Correction method= # Reported T Limits: Tmin=0.669 Tmax=0.746
AbsCorr = MULTI-SCAN

Data completeness= 1.71/1.00 Theta(max)= 27.486

R(reflections)= 0.0389(3167) wR2(reflections)= 0.0972(3445)

S = 1.076 Npar= 203

Figure S90 Crystal information of **14a**

To determine the absolute configuration of **1b**: Firstly, **1b** was recrystallized from dichloromethane/methanol. The solvents were slowly evaporated directly, and the single crystal was obtained after three days. The CCDC number is 2391563.

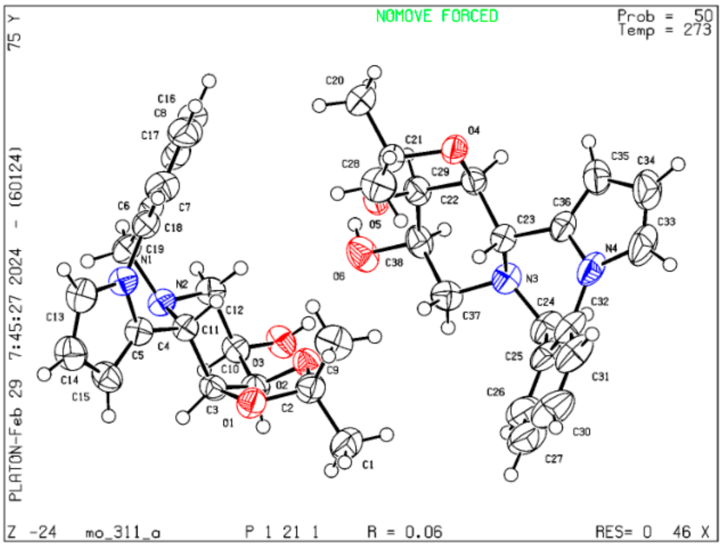

Bond precision: C-C = 0.0070 A Wavelength=0.71073

Cell:	a=12.437(3)	b=10.434(3)	c=14.017(3)
	alpha=90	beta=109.431(9)	gamma=90
Temperature:	273 K		

	Calculated	Reported
Volume	1715.4(7)	1715.3(7)
Space group	P 21	P 1 21 1
Hall group	P 2yb	P 2yb
Moiety formula	C19 H22 N2 O3	C19 H22 N2 O3
Sum formula	C19 H22 N2 O3	C19 H22 N2 O3
Mr	326.39	326.38
Dx,g cm-3	1.264	1.264
Z	4	4
Mu (mm-1)	0.086	0.086
F000	696.0	696.0
F000'	696.31	
h,k,lmax	16,13,18	16,13,18
Nref	8086[4266]	7250
Tmin,Tmax	0.997,0.998	0.997,0.998
Tmin'	0.997	

Correction method= # Reported T Limits: Tmin=0.997 Tmax=0.998
AbsCorr = MULTI-SCAN

Data completeness= 1.70/0.90 Theta(max)= 27.734

R(reflections)= 0.0576(5712) wR2(reflections)= 0.1379(7250)

S = 1.099 Npar= 441

Figure S91 Crystal information of **1b**
